# Synergistic ROS Reduction Through the Co-Inhibition of BRAF and p38 MAPK Ameliorates Senescence

**DOI:** 10.3390/antiox13121465

**Published:** 2024-11-28

**Authors:** Myeong Uk Kuk, Duyeol Kim, Yun Haeng Lee, Jee Hee Yoon, Ji Ho Park, Yoo Jin Lee, Byeong Hyeon So, Minseon Kim, Hyung Wook Kwon, Youngjoo Byun, Joon Tae Park

**Affiliations:** 1 Division of Life Sciences, College of Life Sciences and Bioengineering, Incheon National University, Incheon 22012, Republic of Korea; muk20@inu.ac.kr (M.U.K.); papaya1130@inu.ac.kr (D.K.); yh.lee@inu.ac.kr (Y.H.L.); yoojn0905@inu.ac.kr (J.H.Y.); 202428002@inu.ac.kr (J.H.P.); juli9709@inu.ac.kr (Y.J.L.); tundra@inu.ac.kr (B.H.S.); alstjs0323@inu.ac.kr (M.K.); hwkwon@inu.ac.kr (H.W.K.); 2 Convergence Research Center for Insect Vectors, Incheon National University, Incheon 22012, Republic of Korea; 3 College of Pharmacy, Korea University, Sejong 30019, Republic of Korea

**Keywords:** reactive oxygen species (ROS), BRAF inhibitor, p38 MAPK inhibitor, metallothionein 2A (MT2A)

## Abstract

Reactive oxygen species (ROS)-mediated damage to macromolecules and cellular organelles is one of the major causes of senescence. Therapeutic strategies that lower ROS levels have been proposed as important treatments for senescence, but effective mechanisms for reducing ROS levels have not been discovered. Here, we aimed to find a combination that has a synergistic effect on ROS reduction using senomorphics known to reduce ROS. Combination treatment with BRAF inhibitor SB590885 and p38 MAPK inhibitor SB203580 showed a synergistic effect on ROS reduction compared to treatment with either drug alone. The synergistic effect of ROS reduction through this combination led to a synergistic effect that restored mitochondrial function and ameliorated senescence-associated phenotypes. To elucidate the underlying mechanism by which the synergistic effect of the two drugs reverses senescence, we performed RNA sequencing and identified *metallothionein 2A* (*MT2A*) as a key gene. *MT2A* was upregulated in response to combination therapy, and overexpression of *MT2A* led to a decrease in ROS and subsequent recovery of senescence-associated phenotypes, similar to the effects of combination therapy. Taken together, we found a drug combination that showed synergistic effects on ROS reduction, which contributed to the recovery of senescence-associated phenotypes through *MT2A* gene regulation. This study opens up a new avenue in aging research by demonstrating that combination therapy with existing senomorphics can enhance the ability to reverse senescence and that similar reversal effects can be achieved through gene regulation regulated by combination therapy.

## 1. Introduction

Senescence is a biological process that occurs in all living organisms and is one of the inevitable phenomena. Changes in the structure and function of cellular organelles, especially the deterioration of mitochondria, are the hallmarks of senescence [1]. As senescence progresses, mitochondria undergo structural changes, which eventually lead to functional defects [2]. Damaged mitochondria release electrons in the electron transport complex (ETC) to produce ROS as byproducts [3]. Damaged mitochondria not only produce ROS but also become targets for damage caused by ROS [4]. Increased ROS levels further damage mitochondria, thereby upregulating mitochondrial ROS production. As ROS levels increase, the structure and function of other cellular organelles deteriorate, leading to senescence [5]. This causal relationship is corroborated by the result that ROS activate polyADP-ribose polymerase 1, an enzyme that consumes nicotinamide adenine dinucleotide (NAD^+^), thereby significantly reducing NAD^+^ levels [6]. Since NAD^+^ plays a key role in the response of cells to environmental changes such as oxidative stress [7], decreased NAD^+^ levels result in the inability of cells to respond appropriately to environmental changes, which impairs cellular function and ultimately worsens age-related diseases [8,9,10]. This causal relationship suggests that one of the most promising treatments for age-related diseases may be to modulate ROS-induced oxidative stress.

Senescent cells contribute to the deterioration of tissue function and cause inflammation [11]. Various senotherapeutics targeting senescent cells are known to alleviate the negative effects of senescent cells, extend lifespan, and treat aging [12]. Senotherapeutics include senolytics and senomorphics. For example, senolytics selectively induce apoptosis in senescent cells and remove them from tissues [13]. However, concerns have been reported about the side effects of senolytic drugs, such as tissue atrophy and impaired tissue regeneration due to stem cell depletion [14]. Senomorphics do not induce senescent cell death but target phenotypes specific to senescent cells [15]. Senomorphics target mammalian target of rapamycin (mTOR), nuclear factor kappa light-chain enhancer of activated B cells (NF-κB), information regulator 2-related enzyme 1 (SIRT1), p53, p21, ATM, BRAF, p38 MAPK, and AKT [16]. Senomorphics that target individual signaling pathways are effective in extending lifespan, but given the multifaceted nature of senescent cells due to alterations in multiple signaling pathways, there are inherent limitations to using a single senomorphics [17,18]. These inherent limitations have been addressed by combining individual senomorphics. For example, rapamycin is an mTOR signaling inhibitor that is effective in extending lifespan [19]. Acarbose activates adenosine monophosphate-activated protein kinase, which inhibits mTOR signaling, thereby delaying carbohydrate digestion [20]. Combination treatment with rapamycin and acarbose improved the lifespan of nematodes more than either drug alone [21]. Combinations of senomorphics targeting different signaling pathways may have a synergistic effect in inhibiting senescence and extending lifespan compared to individual drugs, and research is needed to find such combinations.

Senomorphics include GDC0068, SB590885, and SB203580, which inhibit the AKT, BRAF, or p38 MAPK signaling pathways [22,23,24]. Each senomorphic is known to uniquely reverse the senescence process. GDC0068, known as an AKT inhibitor, restores mitochondrial clearance and mitochondrial function by restoring lysosomal function in senescent cells [22]. SB590885, known as a BRAF inhibitor, promotes mitochondrial metabolic reprogramming and restores mitochondrial function [23]. The restoration of mitochondrial function by SB590885 induced cell proliferation and restored senescence-associated phenotypes. SB203580, a p38 MAPK inhibitor, ameliorated lysosomal function as evidenced by increased autophagy function [24]. The restoration of lysosomal function via SB203580 led to metabolic reprogramming through the removal of damaged mitochondria, ultimately reducing ROS levels. Although each senomorphic has been shown to be effective in reversing senescence by modulating each signaling pathway, the efficacy of combination therapy in reversing senescence has not been studied.

In this study, we aimed to find a combination that effectively reduces ROS using inhibitors of AKT, BRAF, and p38 MAPK pathways. The combination of BRAF and p38 MAPK inhibitors showed a synergistic effect on ROS reduction, and this effect led to a synergistic effect on senescence recovery. Through transcriptome analysis, it was found that the synergistic mechanism between the two drugs was achieved by regulating the *MT2A* gene. Here, we discovered a mechanism for ROS reduction and subsequent senescence recovery based on the combination of the two inhibitors or direct regulation of *MT2A* signaling.

## 2. Materials and Methods

### 2.1. Cell Culture

The cells used were human dermal fibroblasts (PCS-201-010; ATCC, Manassas, VA, USA) and HEK 293T (CRL-11268; ATCC). Cell culture was performed according to the method used in a previous study [25]. Cell viability and cell number were investigated using a Cedex HiRes Analyzer (05650216001; Roche, Basel, Switzerland). The doubling time of human dermal fibroblasts was used to classify cells as young or senescent cells. Young fibroblasts doubled every 2 days, and senescent fibroblasts doubled every 14 days.

### 2.2. Flow Cytometric Analysis of Reactive Oxygen Species (ROS)

GDC0068 (S2808; Selleckchem, Houston, TX, USA), SB590885 (SML0501; Sigma, St. Louis, MO, USA), SB203580 (ab120162; Abcam, Cambridge, UK), and SC79 (SML0749; Sigma) were diluted to a final concentration of 5 mM using dimethyl sulfoxide (DMSO, D8418; Sigma, St. Louis, MO, USA). To obtain a concentration of 0.5 μM, 10 mL of medium was mixed with 1 μL of 5 mM GDC0068, 5 mM SB590885, 5 mM SB203580, or 5 mM SC79. By diluting the DMSO in the medium to a concentration of 0.01%, a DMSO control was employed. Specifically, 10 mL of medium was mixed with 1 μL of DMSO. Senescent fibroblasts were treated with DMSO (0.01%), 0.5 μM GDC0068, 0.5 μM SB590885, 0.5 μM SB203580, 0.5 μM GDC0068 and SB590885, 0.5 μM SB590885 and SB203580, 0.5 μM GDC0068 and SB203580, or 0.5 μM GDC0068 and SB590885 and SB203580 for 12 days. For the AKT activation experiment, senescent fibroblasts were treated with DMSO (0.01%), 0.5 μM SC79, 0.5 μM SB590885 and SB203580, or 0.5 μM SB590885 and SB203580 and SC79 for 12 days. To maintain drug concentrations for 12 days, the medium was replaced every 4 days with a medium containing each drug diluted to the corresponding concentration. Following 12 days of drug treatment, cells were incubated for 30 min at 37 °C in media containing 30 μM DHR123 (10056-1; Biotium, Fremont, CA, USA). Cells were then prepared for flow cytometry analysis as described previously [26].

### 2.3. Flow Cytometric Analysis of Mitochondrial Membrane Potential (MMP), Mitochondrial Mass, and Autofluorescence

Senescent fibroblasts were treated with DMSO (0.01%), 0.5 μM SB590885, 0.5 μM SB203580, or 0.5 μM SB590885 and SB203580 for 12 days. To maintain drug concentrations for 12 days, the medium was replaced every 4 days with a medium containing each drug diluted to the corresponding concentration. After 12 days of drug treatment, MMPs were measured by treating senescent fibroblasts with a medium containing 0.6 µg/mL JC-10 (ENZ–52305; Enzo Life Sciences, Farmingdale, NY, USA) for 30 min at 37 °C. To assess mitochondrial mass, senescent fibroblasts were incubated in a medium containing 50 nM MitoTracker Deep Red (M22426; Thermo Fisher Scientific, Waltham, MA, USA) for 30 min at 37 °C. To measure autofluorescence, senescent fibroblasts were incubated in a medium without dye for 30 min at 37 °C. Subsequently, flow cytometry analysis was performed using the methodology of a previous study [26].

### 2.4. Measurement of the Extracellular Acidification Rate (ECAR)

ECAR was measured using the Seahorse XF Glycolysis Rate Assay Kit (103344–100; Aglient Technolongy). The manufacturer’s instructions were followed when using the Seahorse XFe96 analyzer (Aglient Technolongy, Santa Clara, CA, USA).

### 2.5. Senescent Associated–β–Galactosidase (SA–β–gal) Staining

The SA-β-gal staining procedure was performed according to the manufacturer’s instructions (9860; Cell Signaling Technology, Beverly, MA, USA).

### 2.6. Transcriptome Expression Profiling

Senescent fibroblasts were treated with DMSO (0.01%), 0.5 μM SB590885, 0.5 μM SB203580, or 0.5 μM SB590885 and SB203580 for 12 days. Then, the experiments were performed in triplicate for each group (DMSO (0.01%), SB590885, SB203580, and SB590885 and SB203580). To maintain drug concentrations for 12 days, the medium was replaced every 4 days with a medium containing each drug diluted to the corresponding concentration. The total RNA was produced using the RNase Mini Kit (74104; QIAGEN, Hilden, Germany) according to the manufacturer’s instructions. Then, RNA sequencing was performed in triplicate for each group (DMSO (0.01%), SB590885, SB203580, and SB590885 and SB203580). More specifically, the expression of transcripts generated using Illumina sequencing (paired-end) was examined. The analysis results of the 101 bp raw reads obtained from sequencing were improved by removing contamination artifacts. Aligned reads were generated from the sequences with contamination artifacts removed using HISAT2 (version 2.1.0; Johns Hopkins University Center for Computational Biology, Baltimore, MD, USA), which were then mapped to the Homo sapiens genome (GRCh38, NCBI_109.20200522). Transcriptome assembly was performed using String Tie (version 2.1.3b; Johns Hopkins University Center for Computational Biology) using the aligned reads. Gene set enrichment analysis was performed to identify differentially expressed genes.

### 2.7. Lenti–Viral Production and Infection

HEK 293T cells were transfected with 8 μg of plasmids (pLenti_control or pLenti_MT2A), 4 μg of PAX2 plasmid, and 4 μg of VSV.G plasmid using Lipofectamine 2000 (11668019; Invitrogen). Viral supernatants were collected 24 h after transfection. Viral supernatants were mixed with 8 μg/mL polybrene (TR–1003–G; Millipore, Burlington, Middlesex County, MA, USA). Senescent fibroblasts were infected with viruses as described previously [27].

### 2.8. Complementary DNA (cDNA) Preparation and Quantitative Polymerase Chain Reaction (qPCR)

cDNA was prepared as previously mentioned [25]. qPCR was carried out as previously mentioned [25]. qPCR was performed using the following primer (Table 1).

### 2.9. Statistical Analysis

Statistical analyses were performed using a statistical software package (SigmaPlot 12.5; Systat Software, San Jose, CA, USA). We assessed the significance of differences using Student’s *t*-test and two-way ANOVA followed by Bonferroni’s post-hoc test.

## 3. Results

### 3.1. The Combination of SB590885 and SB203580 Is Most Effective in Reducing ROS Levels

**Figure 1:**
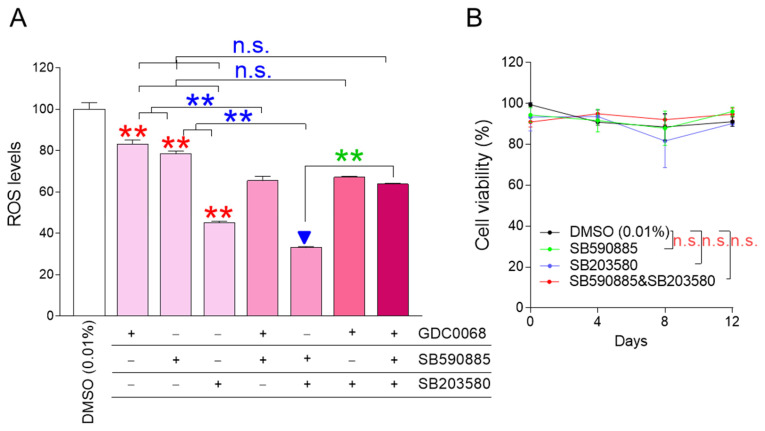
The combination of SB590885 and SB203580 is the most effective in reducing ROS levels. (**A**) GDC0068 (0.5 μM), SB590885 (0.5 μM), and SB203580 (0.5 μM) were used individually or in all possible dual and triple combinations. ROS levels were measured using DHR123 after 12 days of treatment. Red asterisks: GDC0068, SB590885, and SB203580 effectively reduced ROS levels when administered alone. Blue asterisks: co-treatment of senescent fibroblasts with GDC0068 and SB590885 significantly reduced ROS levels compared to treatment with either GDC0068 or SB590885. Blue asterisks: co-treatment of senescent fibroblasts with SB590885 and SB203580 significantly reduced the ROS levels compared to treatment with either SB590885 or SB203580. Green asterisks: AKT inhibition in addition to concurrent inhibition of BRAF and p38 MAPK (triple combination) increased the ROS levels. ** *p* < 0.01, n.s. (not significant), Student’s *t*-test. Mean ± SD, N = 3. The combination of SB590885 and SB203580 most effectively reduced ROS levels (blue arrowhead). (**B**) Cell viability was measured at 0, 4, 8, and 12 days after each treatment. n.s. (not significant), two-way ANOVA followed by Bonferroni’s post-hoc test. Mean ± S.D., N = 3.

ROS is known to decrease when certain cellular signaling pathways, such as AKT, BRAF, and p38 MAPK signaling pathways, are inhibited, and GDC0068, SB590885, and SB203580 were effective in inhibiting each cellular signaling pathway, respectively [22,23,24]. We aimed to identify combinations that could effectively reduce ROS levels when AKT, BRAF, and p38 MAPK were inhibited individually or in all possible dual and triple combinations. GDC0068, SB590885, and SB203580 effectively reduced ROS levels when administered alone, confirming previous results [22,23,24] (Figure 1A; red asterisks). Co-treatment of senescent fibroblasts with GDC0068 and SB590885 significantly reduced ROS levels compared to treatment with either GDC0068 or SB590885, suggesting that concurrent inhibition of AKT and BRAF signaling has a synergistic effect in reducing ROS (Figure 1A; blue asterisks). Furthermore, the co-treatment of senescent fibroblasts with SB590885 and SB203580 significantly reduced the ROS levels compared to treatment with either SB590885 or SB203580, suggesting that concurrent inhibition of BRAF and p38 MAPK signaling has a synergistic effect in reducing ROS (Figure 1A; blue asterisks). However, co-treatment of senescent fibroblasts with GDC0068 and SB203580 did not significantly reduce ROS levels compared to treatment with SB203580 (Figure 1A). Moreover, the triple combination of GDC0068, SB590885, and SB203580 did not significantly reduce ROS levels compared with SB203580 (Figure 1A). These results suggest that ROS-reducing efficacy is synergistic only when specific signal combinations are co-inhibited and antagonistic when other signal combinations are inhibited. In summary, combined treatment with SB590885 and SB203580 in senescent fibroblasts was most effective in reducing ROS.

The finding that co-treatment with SB590885 and SB203580 was effective in reducing ROS levels prompted us to explore additional signaling modulations that might further enhance ROS reduction. AKT inhibition in addition to concurrent inhibition of BRAF and p38 MAPK (triple combination) increased the ROS levels (Figure 1A; green asterisks). These results raised the question of how AKT activation in addition to concurrent inhibition of BRAF and p38 MAPK would affect ROS levels. Therefore, additional experiments were performed to activate AKT using the AKT activator SC79 [27]. The treatment of senescent fibroblasts with SC79 significantly increased ROS levels compared to DMSO (0.01%) (Figure S1). Co-treatment of senescent fibroblasts with SB590885 and SB203580 significantly reduced the ROS levels compared to DMSO (0.01%) (Figure S1). However, AKT activation in addition to concurrent inhibition of BRAF and p38 MAPK did not reduce ROS levels but rather slightly increased them (Figure S1). These results suggest that AKT activation in addition to concurrent inhibition of BRAF and p38 MAPK is not effective in reducing ROS levels.

Having found that co-treatment with SB590885 and SB203580 was the optimal condition for reducing ROS, we investigated how each treatment condition affected cell viability. During 12 days of drug treatment, senescent fibroblasts treated with SB590885 or SB203580 showed similar viability to senescent fibroblasts treated with DMSO (0.01%), suggesting that treatment with SB590885 or SB203580 did not affect cell viability (Figure 1B). Furthermore, senescent fibroblasts co-treated with SB590885 and SB203580 showed similar viability to senescent fibroblasts treated with DMSO (0.01%), suggesting that the co-treatment did not affect cell viability (Figure 1B).

The finding that co-treatment with SB590885 and SB203580 did not affect the viability finally led us to investigate whether ROS reduction by co-treatment with SB590885 and SB203580 was due to the drugs themselves. To demonstrate this, we investigated how dose-dependent co-treatment with SB590885 and SB203580 affected the ROS levels. Since the drug concentrations used in co-treatment with SB590885 and SB203580 were 0.5 μM, respectively, the co-treatment concentrations were serially diluted from 0.5 to 0.25, 0.125, 0.0625, and 0.03125 μM. Senescent fibroblasts were then treated with each concentration and the ROS levels were examined. Each co-treatment concentration significantly reduced the ROS levels compared with DMSO (0.01%) (Figure S2). Furthermore, the ROS levels decreased in a dose-dependent manner as the co-treatment concentration increased (Figure S2). These results suggest that ROS reduction in senescent fibroblasts was induced by co-treatment with SB590885 and SB203580.

### 3.2. Co-Treatment with SB590885 and SB20358 Synergistically Restores Mitochondrial Function

**Figure 2:**
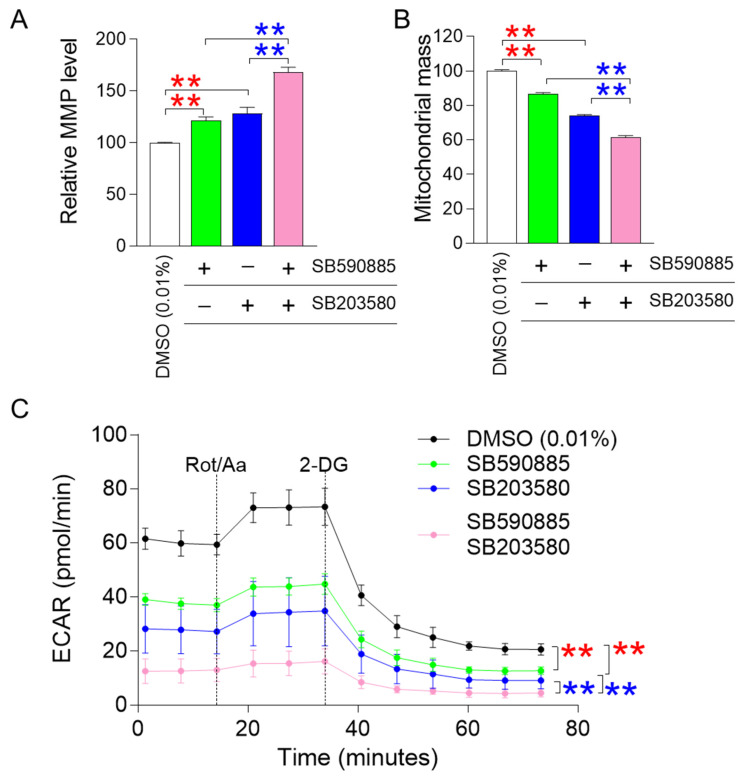
Co-treatment with SB590885 and SB20358 synergistically restores mitochondrial function. (**A**) Measurement of mitochondrial membrane potential (MMP) using JC-10 dye. Red asterisks: senescent fibroblasts treated with either SB590885 or SB203580 had a significant increase in MMP compared to DMSO (0.01%). Blue asterisks: senescent fibroblasts co-treated with SB590885 and SB203580 had a significant increase in MMP compared to either SB590885 or SB203580. ** *p* < 0.01, Student’s *t*-test. Mean ± SD, N = 3. (**B**) Measurement of mitochondrial mass using MitoTracker Deep Red. Red asterisks: senescent fibroblasts treated with either SB590885 or SB203580 had a significant decrease in mitochondrial mass compared to DMSO (0.01%). Blue asterisks: senescent fibroblasts co-treated with SB590885 and SB203580 showed a significant decrease in mitochondrial mass compared to either SB590885 or SB203580. ** *p* < 0.01, Student’s *t*-test. Mean ± SD, N = 3. (**C**) Measurement of extracellular acidification rate (ECAR). The black line represents the group treated with DMOS (0.01%), the green line represents the group treated with SB590885, the blue line represents the group treated with SB203580, and the pink line represents the group co-treated with SB590885 and SB203580. Red asterisks: senescent fibroblasts treated with either SB590885 or SB203580 showed a significant decrease in ECAR value compared to DMSO (0.01%). Blue asterisks: senescent fibroblasts co-treated with SB590885 and SB203580 showed a significant decrease in ECAR value compared to either SB590885 or SB203580. ** *p* < 0.01, two-way ANOVA followed by Bonferroni’s post-hoc test. Mean ± S.D., N = 6.

ROS-induced damage directly affects mitochondrial membrane potential (MMP), an electrical potential generated by protons moving from the mitochondrial matrix to the intermembrane space [28]. Since MMP is an electrical potential that promotes ATP production in mitochondria [29], an increase in MMP suggests mitochondrial functional recovery [30]. The finding that combined treatment with SB590885 and SB203580 had a synergistic effect in reducing ROS prompted us to investigate how co-treatment affected MMPs. Senescent fibroblasts treated with either SB590885 or SB203580 showed a significant increase in MMP compared to DMSO (0.01%) (Figure 2A). In addition, senescent fibroblasts co-treated with SB590885 and SB203580 showed a significant increase in MMP compared to those treated with either SB590885 or SB203580, indicating that the co-treatment with both drugs had a synergetic effect on the increase in MMP (Figure 2A).

Altered mitochondrial morphology, including increased mitochondrial mass, is a hallmark of senescent cells [31,32]. Specifically, a senescence-associated lysosomal dysfunction limits efficient mitochondrial clearance and leads to the accumulation of abnormally large and defective mitochondria [33]. Therefore, a decrease in mitochondrial mass conversely indicates restoration of mitochondrial function [31,32]. Since the increase in MMP is a representative indicator of mitochondrial function recovery, we investigated how the combined treatment with SB590885 and SB203580 affected mitochondrial mass. Senescent fibroblasts treated with either SB590885 or SB203580 exhibited a significant decrease in mitochondrial mass compared to DMSO (0.01%) (Figure 2B). In addition, senescent fibroblasts co-treated with SB590885 and SB203580 showed a significant decrease in mitochondrial mass compared to those treated with either SB590885 or SB203580, indicating that co-treatment with both drugs has a synergetic effect on the decrease in mitochondrial mass (Figure 2B).

A decrease in mitochondrial function increases reliance on glycolysis as an energy source, whereas recovery of mitochondrial function decreases reliance on glycolysis [34]. Given that co-treatment with SB590885 and SB203580 synergistically induced recovery of mitochondrial function, we investigated whether changes in reliance on glycolysis as an energy source also occurred synergistically. The glycolysis rate was measured using the extracellular acidification rate (ECAR) after sequential administration of rotenone/antimycin A (Rot/AA) and 2-deoxy-D-glucose (2-DG). The basal glycolysis rate (before Rot/AA administration), compensatory glycolysis rate (after Rot/AA administration), and post-2-DG acidification (after 2-DG administration) were all evaluated using the successively measured ECAR values [35]. Before the administration of mitochondrial inhibitors (Rot/AA) that inhibit oxidative phosphorylation and induce a compensatory shift toward glycolysis [36], senescent fibroblasts treated with SB590885 or SB203580 exhibited a significant decrease in ECAR values compared to DMSO (0.01%) (Figure 2C). These data indicate that senescent fibroblasts treated with either SB590885 or SB203580 had a lower basal glycolytic rate than DMSO (0.01%) (Figure 2C). Furthermore, before the administration of Rot/AA, senescent fibroblasts co-treated with SB590885 and SB203580 exhibited a significant decrease in ECAR values compared to senescent fibroblasts treated with either SB590885 or SB203580, indicating that co-treatment with SB590885 and SB203580 acted synergistically in reducing the basal glycolytic rate (Figure 2C). After Rot/AA administration, compensatory glycolysis was significantly lower in senescent fibroblasts treated with SB590885 or SB203580 compared to DMSO (0.01%), suggesting that treatment with either SB590885 or SB203580 reduced the need for glycolysis to meet energy demands (Figure 2C). Furthermore, after Rot/AA administration, senescent fibroblasts co-treated with SB590885 and SB203580 showed significantly reduced ECAR values compared to senescent fibroblasts treated with either SB590885 or SB203580, indicating that co-treatment with SB590885 and SB203580 synergistically reduced compensatory glycolysis (Figure 2C). When 2-DG, which inhibits glycolysis, was added [37], senescent fibroblasts treated with SB590885 or SB203580 showed significantly lower ECAR values compared to DMSO (0.01%) (Figure 2C). These data suggest that senescent fibroblasts treated with either SB590885 or SB203580 have low residual glycolysis that is not completely inhibited by 2-DG, or in other words, have low post-2-DG acidification [38] (Figure 2C). Furthermore, after 2-DG administration, senescent fibroblasts co-treated with SB590885 and SB203580 showed significantly decreased ECAR values compared to the SB590885 or SB203580 groups, indicating that co-treatment with SB590885 and SB203580 had a synergistic effect on reducing post-2-DG acidification (Figure 2C).

### 3.3. Co-Treatment of SB590885 and SB20358 Synergistically Restores Senescence-Associated Phenotypes

**Figure 3:**
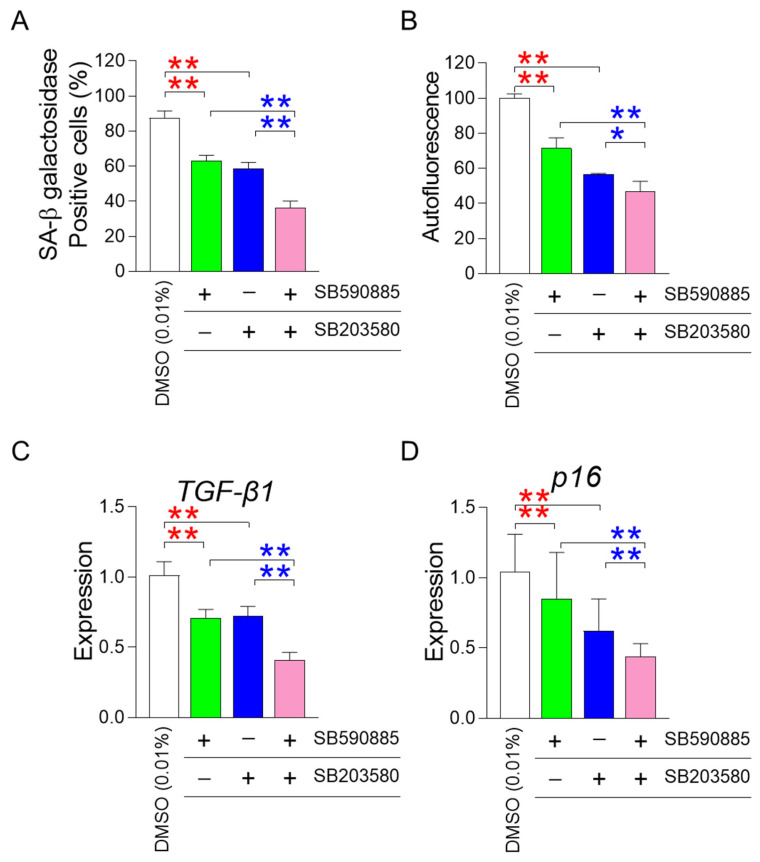
Co-treatment of SB590885 and SB20358 synergistically restores senescence-associated phenotypes. (**A**) Measurement of the percentage of senescence-associated-β-galactosidase (SA-β-gal) positive cells. Red asterisks: senescent fibroblasts treated with either SB590885 or SB203580 showed a significant decrease in the percentage of SA-β-gal positive cells compared to DMSO (0.01%). Blue asterisks: senescent fibroblasts co-treated with SB590885 and SB203580 showed a significant decrease in the percentage of SA-β-gal positive cells compared to either SB590885 or SB203580. ** *p* < 0.01, Student’s *t*-test. Mean ± SD, N = 3. (**B**) Measurement of autofluorescence. Red asterisks: senescent fibroblasts treated with either SB590885 or SB203580 showed a significant decrease in autofluorescence compared to DMSO (0.01%). Blue asterisks: senescent fibroblasts co-treated with SB590885 and SB203580 showed a significant decrease in autofluorescence compared with either SB590885 or SB203580. ** *p* < 0.01, * *p* < 0.05, Student’s *t*-test. Mean ± SD, N = 3. (**C**) Expression levels of the *TGF-β1* gene. Red asterisks: senescent fibroblasts treated with either SB590885 or SB203580 showed a significant decrease in the expression of *TGF-β1* compared to DMSO (0.01%). Blue asterisks: senescent fibroblasts co-treated with SB590885 and SB203580 showed a significant decrease in the expression of *TGF-β1* compared with either SB590885 or SB203580. ** *p* < 0.01, Student’s *t*-test. Mean ± SD, N = 3. (**D**) Expression levels of the *p16* gene. Red asterisks: senescent fibroblasts treated with either SB590885 or SB203580 showed a significant decrease in the expression of *p16* compared to DMSO (0.01%). Blue asterisks: senescent fibroblasts co-treated with SB590885 and SB203580 showed a significant decrease in the expression of *p16* compared with either SB590885 or SB203580. ** *p* < 0.01, Student’s *t*-test. Mean ± SD, N = 3.

One of the requisites for improving senescence is the restoration of mitochondrial function [22,23,24]. After finding that the co-treatment of SB590885 and SB203580 had a synergistic effect on restoring mitochondrial function, we investigated how the co-treatment affected senescence-associated phenotypes. First, we investigated the effect of co-treatment with SB590885 and SB203580 on senescence-associated β-galactosidase (SA-β-gal), a widely used senescence marker [39]. Compared with DMSO (0.01%), the treatment of senescent fibroblasts with either SB590885 or SB203580 significantly reduced the percentage of SA-β-gal positive cells (Figure 3A). Furthermore, senescent fibroblasts co-treated with SB590885 and SB203580 showed a significant decrease in the percentage of SA-β-gal positive cells compared to those treated with either SB590885 or SB203580, indicating that the co-treatment of SB590885 and SB203580 was synergistic in reducing the percentage of SA-β-gal positive cells (Figure 3A).

In addition to SA-β-gal, the effect of co-treatment on autofluorescence, which indicates the accumulation of senescence-related substances such as lipofuscin, was also evaluated [40]. Senescent fibroblasts treated with either SB590885 or SB203580 showed a significant decrease in autofluorescence compared to DMSO (0.01%) (Figure 3B). Furthermore, senescent fibroblasts co-treated with SB590885 and SB203580 showed significantly reduced autofluorescence compared to either SB590885 or SB203580, indicating that co-treatment with SB590885 and SB203580 was synergistic in reducing autofluorescence (Figure 3B).

After finding that co-treatment of SB590885 and SB203580 had a synergistic effect on restoring senescence-associated phenotypes, we investigated the effect of co-treatment on the senescence-associated secretory phenotype (SASP). As transforming growth factor-β1 (TGF-β1) is secreted as one of the SASP factors and can induce age-related pathological conditions [41], the effect of co-treatment on the expression level of *TGF-β1* was examined. Compared with DMSO (0.01%), treatment of senescent fibroblasts with either SB590885 or SB203580 significantly reduced the expression of *TGF-β1* (Figure 3C). Furthermore, senescent fibroblasts co-treated with SB590885 and SB203580 showed a significant decrease in the expression of *TGF-β1* compared to those treated with either SB590885 or SB203580, indicating that the co-treatment with SB590885 and SB203580 was synergistic in reducing the expression of *TGF-β1* (Figure 3C).

Finally, we investigated the effect of co-treatment on cell cycle arrest, one of the defining characteristics of senescence [42]. As p16 arrests the cell cycle at the G1 phase [43], the effect of co-treatment on the expression level of *p16* was examined. Compared with DMSO (0.01%), treatment of senescent fibroblasts with either SB590885 or SB203580 significantly reduced the expression of *p16* (Figure 3D). Furthermore, senescent fibroblasts co-treated with SB590885 and SB203580 showed a significant decrease in the expression of *p16* compared to those treated with either SB590885 or SB203580, indicating that the co-treatment with SB590885 and SB203580 was synergistic in reducing the expression of *p16* (Figure 3D).

### 3.4. RNA Sequencing Identifies MT2A as a Key Regulator in the Synergistic Mechanism Induced by Co-Treatment

**Figure 4:**
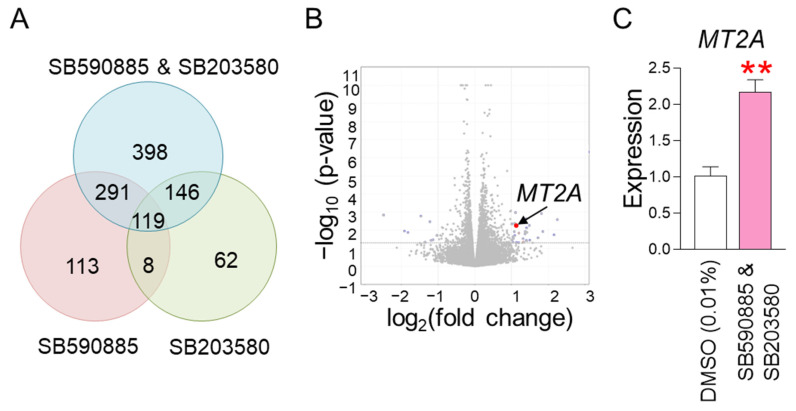
RNA sequencing identifies MT2A as a key regulator in the synergistic mechanism induced by co-treatment. (**A**) DEG analysis selected 1137 genes that were significantly altered more than 2-fold compared to DMSO (0.01%). Of the total 1137 genes, 398 genes that were specifically altered by co-treatment with SB590885 and SB203580 were focused on. (**B**) A candidate approach was performed to investigate which of the 398 genes are involved in ROS production or suppression. *Metallothionein 2A* (*MT2A*) was selected as a candidate because it is rich in cysteine and acts as an antioxidant by binding to divalent heavy metal ions. (**C**) Expression levels of MT2A gene. Co-treatment of senescent fibroblasts with SB590885 and SB203580 resulted in a more than 2-fold increase in MT2A expression compared to DMSO (0.01%). ** *p* < 0.01, Student’s *t*-test. Mean ± SD, N = 3.

The finding that co-treatment with SB590885 and SB203580 had a synergistic effect in restoring senescence-associated phenotypes raised the question of through what mechanism the co-treatment could synergistically restore senescence. To elucidate the underlying mechanism, RNA sequencing was performed. Senescent fibroblasts were treated with DMSO (0.01%), SB590885, SB203580, or SB590885 and SB203580. Data from transcriptome sequencing of human fibroblasts were used to analyze differentially expressed genes (DEG). DEG analysis selected 1137 genes that were significantly changed more than 2-fold compared to DMSO (0.01%) (Table S1 and Figure 4A). Of the total 1137 genes, we focused on 398 genes that were specifically altered by co-treatment with SB590885 and SB203580 (Table S2 and Figure 4A). These genes were not significantly altered by either SB590885 or SB203580 but were significantly altered by co-treatment with SB590885 and SB203580, making them suitable for elucidating the synergistic mechanism of SB590885 and SB203580.

We then performed a candidate approach to investigate which of the 398 genes are involved in ROS production or suppression. *Metallothionein 2A* (*MT2A*) was selected as a candidate because it is rich in cysteine and acts as an antioxidant by binding to divalent heavy metal ions [44] (Figure 4B). In addition, RNA sequencing analysis showed that the expression of *MT2A* increased more than 2-fold when co-treated with SB590885 and SB203580 (Table S2), which was confirmed by quantitative PCR as a more than 2-fold increase compared to DMSO (0.01%) (Figure 4C). Therefore, *MT2A* was selected as a key candidate that synergistically reduces ROS when co-treated with SB590885 and SB203580.

### 3.5. MT2A Overexpression Reduces ROS Levels and Restores Senescence-Associated Phenotypes

**Figure 5:**
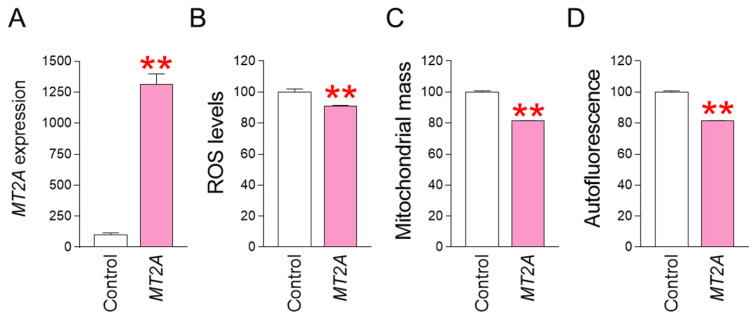
*MT2A* overexpression reduces ROS levels and restores senescence-associated phenotypes. (**A**) Upon infection of senescent fibroblasts with the lentivirus expressing *MT2A*, the expression of *MT2A* significantly increased compared to senescent fibroblasts infected with the control lentivirus. ** *p* < 0.01, Student’s *t*-test. Means ± S.D., N = 3. (**B**) Measurement of ROS levels using DHR123. Compared with senescent fibroblasts infected with the control lentivirus, senescent fibroblasts infected with the lentivirus expressing *MT2A* showed a significant decrease in ROS levels. ** *p* < 0.01, Student’s *t*-test. Means ± S.D., N = 3. (**C**) Measurement of mitochondrial mass using MitoTracker Deep Red. Compared with senescent fibroblasts infected with the control lentivirus, senescent fibroblasts infected with the lentivirus expressing *MT2A* showed a significant decrease in mitochondrial mass. ** *p* < 0.01, Student’s *t*-test. Means ± S.D., N = 3. (**D**) Measurement of autofluorescence. Compared with senescent fibroblasts infected with the control lentivirus, senescent fibroblasts infected with the lentivirus expressing *MT2A* showed a significant decrease in autofluorescence. ** *p* < 0.01, Student’s *t*-test. Means ± S.D., N = 3.

*MT2A* was identified as a key gene that acts when co-treatment with SB590885 and SB203580 synergistically reduces ROS. To determine whether regulating *MT2A* expression has the same effect on reducing ROS and reversing senescence, we investigated the effect of *MT2A* overexpression in senescent fibroblasts. Specifically, after establishing a lentiviral system for overexpressing *MT2A*, senescent fibroblasts were infected with *MT2A*-expressing lentivirus or a control lentivirus. Compared with senescent fibroblasts infected with a control lentivirus, senescent fibroblasts infected with a lentivirus expressing *MT2A* significantly increased *MT2A* expression, indicating efficient *MT2A* gene transfer by the lentivirus (Figure 5A). We then investigated how *MT2A* overexpression affected ROS levels. Compared with senescent fibroblasts infected with the control lentivirus, senescent fibroblasts infected with the lentivirus expressing *MT2A* showed a significant decrease in ROS levels, indicating that *MT2A* overexpression had the same ROS-reducing effect as that induced by the combination of SB590885 and SB203580 (Figure 5B). Given that ROS-induced mitochondrial damage increases mitochondrial mass as a compensatory mechanism [31,32], we investigated how *MT2A*-mediated ROS reduction affects mitochondrial mass. Compared with senescent fibroblasts infected with the control lentivirus, senescent fibroblasts infected with the lentivirus expressing *MT2A* showed a significant decrease in mitochondrial mass, indicating that *MT2A* overexpression had the same mitochondrial mass-reducing effect as that induced by the combination of SB590885 and SB203580 (Figure 5C). Finally, we investigated how *MT2A* overexpression affects autofluorescence, one of the senescence-associated phenotypes [40]. Compared with senescent fibroblasts infected with the control lentivirus, senescent fibroblasts infected with the lentivirus expressing *MT2A* showed a significant decrease in autofluorescence, indicating that *MT2A* overexpression had the same autofluorescence-reducing effect as that induced by the combination of SB590885 and SB203580 (Figure 5D).

## 4. Discussion

Senescence is a multifaceted process driven by various signaling pathways, including mTOR, NF-κB, SIRT1, p53, p21, ATM, BRAF, p38 MAPK, and AKT [22,23,24,45,46,47]. Senomorphics, such as rapamycin, metformin, and resveratrol, have been developed to target specific signaling pathways and provide a pharmacological approach to target cellular senescence [48]. Although individual Senomorphic agents are effective in ameliorating senescence, the multifaceted nature of senescence, which involves alterations in multiple signaling pathways, limits the ability of a single senomorphic agent to completely reverse senescence [17,18]. Therefore, it has been proposed that the simultaneous use of senomorphics targeting each signaling pathway may better modulate senescence [21]. In our previous study, we found that the BRAF inhibitor SB590885 ameliorated senescence by inducing mitochondrial function recovery and reducing ROS [23]. In addition, the p38 MAPK inhibitor SB203580 restored senescence by activating autophagy to remove damaged mitochondria, ultimately reducing ROS levels [24]. Although SB590885 and SB203580 inhibit different signaling pathways, their complementary mechanisms of action were expected to be more effective in reversing senescence. Here, we demonstrated that the combined treatment of SB590885 and SB203580 had a synergistic effect on senescence recovery. In particular, the underlying mechanism of the improvement in senescence was attributed to the finding that the co-treatment of SB203580 and SB590885 reduced ROS levels more significantly than either drug used individually. Extending these results, the synergistic effect on ROS reduction further improved mitochondrial function through MMP increase and mitochondrial metabolic reprogramming. The co-treatment-mediated restoration of mitochondrial function, which is considered a prerequisite for reversing senescence [22,23,24], also had a synergistic effect on the recovery of senescence-associated phenotypes. Thus, we propose that co-treatment with SB590885 and SB203580 may be effective in treating aging and age-related diseases in which the efficacy of single senomorphics has been limited.

Oxidative stress-induced damage to cellular organelles is one of the major causes of aging [49]. The major organelles that generate ROS in cells are mitochondria [50]. Mitochondria consume more than 90% of oxygen, and ETC complexes convert 1–5% of oxygen to O_2_^●−^ [51]. Specifically, complexes I and III within the mitochondrial matrix convert oxygen to O_2_^●−^. Complex III also generates O_2_^●−^ in the mitochondrial intermembrane space. Mitochondrial dysfunction associated with senescence reduces the activity of ETC complexes [52,53]. In particular, decreased activity of complex I leads to inefficient electron transport, which increases electron leak to oxygen and consequently increases O_2_^●−^ production [54]. ETC damage is further aggravated by increased ROS production in mitochondria, which in turn increases ROS production in mitochondria [55]. As a result of this harmful cycle, the structure and function of cellular organelles deteriorate, ultimately leading to senescence [56]. The causal relationship emphasizes that an important strategy to reverse senescence is to reduce ROS production in mitochondria [55]. However, an efficient approach to modulate ROS production in mitochondria has not been well demonstrated. Previous studies have shown that *MT2A* significantly reduces ROS production by protecting complex I activity [57]. In this study, we discovered a unique mechanism by which co-treatment with SB590885 and SB203580 can reduce mitochondrial ROS production through upregulating *MT2A*. Co-treatment with SB590885 and SB203580 may protect complex I activity by upregulating *MT2A*, which is indirectly supported by our finding that compensatory glycolysis mediated by complex I inhibitor Rot and complex III inhibitor AA was significantly reduced in senescent fibroblasts co-treated with SB590885 and SB203580. The reduction in compensatory glycolysis indicates that cells are becoming more reliant on oxidative phosphorylation (OXPHOS) to generate ATP as their intracellular energy source [58]. The metabolic shift from glycolysis to OXPHOS implies active OXPHOS based on increased activity of complex I, a component of the ETC [59]. Moreover, a co-treatment-mediated increase in MMP indicates increased proton transport from the mitochondrial matrix to the mitochondrial intermembrane space and subsequent activation of electron transport within the mitochondrial ETC [60], which also suggests protection of complex I activity. Subsequently, efficient electron transport induced by active complex I reduced electron leakage to oxygen, thereby reducing the production of ROS by-products [61]. Therefore, we propose a novel mechanism by which co-treatment with SB590885 and SB203580 protects complex I activity via upregulation of *MT2A*, enabling efficient electron transport and reducing ROS formation in mitochondria.

BRAF belongs to the serine/threonine protein kinase family that plays a related role in the mitogen-activated protein kinase (MAPK)/extracellular signal-regulated kinase [62]. BRAF has a wide range of downstream targets with diverse cellular functions. p38 MAPK belongs to the evolutionarily highly conserved MAPK family [63]. To maintain cellular homeostasis, p38 MAPK regulates the activity of downstream targets in response to various physiological stimuli. BRAF and p38 MAPK have unique downstream targets and co-regulated downstream target genes, but no studies have been conducted to identify these targets. In this study, we identified *MT2A* as a key gene that is co-regulated when BRAF and p38 MAPK are simultaneously inhibited. Since the regulation of co-regulated downstream genes is known to have similar effects as the individual regulation of each upstream gene [64,65,66], we tested whether the regulation of *MT2A* could achieve similar effects. Indeed, modulation of *MT2A* induced the same ROS-reducing effect as observed in the co-treatment of SB590885 and SB203580. The ROS reduction by *MT2A* modulation induced senescence improvement by decreasing mitochondrial mass and autofluorescence. These results indicate that modulation of *MT2A* ameliorates senescence as the combination treatment. Taken together, our findings reveal a novel role for *MT2A* as a key regulator of the synergistic effect induced by the co-treatment of SB590885 and SB203580.

MT2A is rich in cysteine and binds to divalent heavy metal ions, scavenging ROS free radicals through its cysteine residues [44]. The antioxidant capacity of MT2A is supported by studies showing that MT2A is 100 times more capable of scavenging free •OH and superoxide radicals than the antioxidant glutathione [67]. In this study, we discovered a novel mechanism by which co-treatment with SB590885 and SB203580 increased *MT2A* expression by more than 2-fold. The increased expression of *MT2A*, which acts as an antioxidant, significantly reduced ROS levels in senescent fibroblasts with high ROS levels. Extending the relevance of these results, overexpression of *MT2A* in senescent fibroblasts reduced ROS and restored senescence-related phenotypes, confirming that the overexpression of *MT2A* has similar effects as co-treatment with SB590885 and SB203580. Based on these results, we conclude that concurrent inhibition of BRAF and p38 MAPK might be one of the potential means to significantly reduce ROS levels in senescent fibroblasts by upregulating *MT2A* expression. However, we admit that further studies are needed to determine whether increased expression of *MT2A* activates the antioxidant capacity of *MT2A* to scavenge ROS free radicals via cysteine residues.

## 5. Conclusions

In conclusion, combination treatment with SB590885 and SB203580 showed a synergistic effect on ROS reduction compared to either treatment alone. The synergistic effect on ROS reduction resulted in synergistic restoration of mitochondrial function and senescence-associated phenotypes. Furthermore, the synergistic effect of the two drugs was *MT2A* upregulation, and *MT2A* overexpression also showed a similar effect as the combination treatment. Our findings provide a novel mechanism by which combination treatment with existing senomorphics can enhance the ability to reverse senescence. Future studies should focus on exploring the clinical applicability of this combination therapy in aging and age-related diseases.

## Figures and Tables

**Table 1:** Details of primers used in qPCR.

Target	Orientation	Sequence (5′-3′)	Size (bp)
*36B4* (Genbank accession number: M17885)	forward	CAGCAAGTGGGAAGGTGTAATCC	23
	reverse	CCCATTCTATCATCAACGGGTACAA	25
*TGF-β1* (Genbank accession number: NM_000660.7)	forward	TGACGTCACTGGAGTTGTACGG	22
	reverse	GGTTCATGTCATGGATGGTGC	21
*p16* (Genbank accession number: NM_000077)	forward	CCCAACGCCCCGAACT	16
	reverse	GCAGAAGAGCTGCTACGTGAA	21
*MT2A* (Genbank accession number: NM_005953)	forward	CGAACCCGCGTGCAAC	16
	reverse	TGCAGATGCAGCCCTGG	17

## Data Availability

The original contributions presented in the study are included in the article; further inquiries can be directed to the corresponding authors.

## Supplementary Materials

The following supporting information can be downloaded at https://www.mdpi.com/article/10.3390/antiox13121465/s1, Figure S1: The effect of AKT activation on ROS levels; Figure S2: ROS reduction by co-treatment with SB590885 and SB203580 is due to the drugs themselves; Table S1: List of 1137 genes significantly changed by more than 2-fold compared to DMSO (0.01%) after treatment with SB590885, SB203580, or a combination of SB590885 and SB203580; Table S2: List of 398 genes that were not significantly changed by SB590885 or SB203580 but were significantly changed by co-treatment with SB590885 and SB203580.
