# Supplementary material for: Synergistic ROS Reduction Through the Co-Inhibition of BRAF and p38 MAPK Ameliorates Senescence
Source: Antioxidants (Basel). 2024 Nov 28;13(12):1465. doi: 10.3390/antiox13121465 (PMC11672831; doi:10.3390/antiox13121465)
Supplement: Supplementary file 1 [file antioxidants-13-01465-s001.zip › antioxidants-3288126-supplementary.pdf]

Figure S1

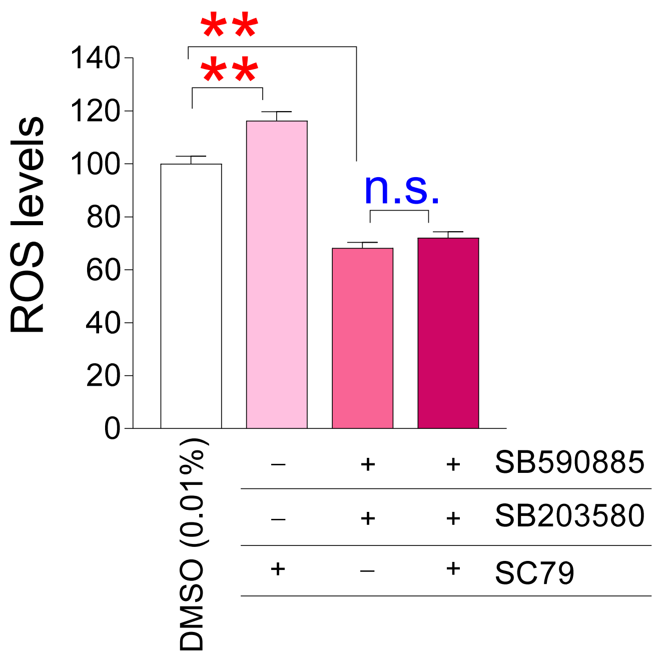

Figure S1. The effect of AKT activation on ROS levels. ROS levels were measured using DHR123 after 12 days of treatment. Treatment of senescent fibroblasts with SC79 significantly increased ROS levels compared to DMSO (0.01%). Co-treatment of senescent fibroblasts with SB590885 and SB203580 significantly reduced ROS levels compared to DMSO (0.01%). However, AKT activation (with AKT activator SC79) in addition to concurrent inhibition of BRAF and p38 MAPK is not effective in reducing ROS levels. $^{**}P < 0.01$, n.s. (not significant), Student's t-test. Mean ± SD, N = 3.

Figure S2

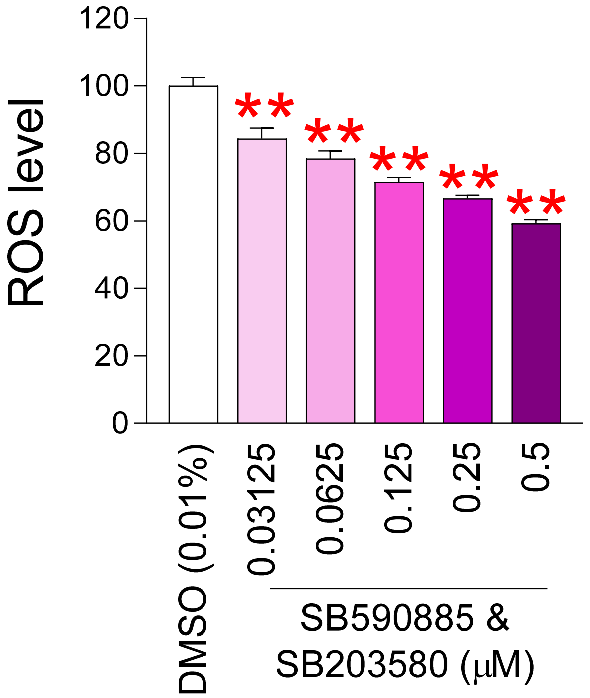

Figure S2. ROS reduction by co-treatment with SB590885 and SB203580 is due to the drugs themselves. ROS levels were measured using DHR123 after 12 days of treatment. Each co-treatment concentration significantly reduced the ROS levels compared with DMSO (0.01%). ROS levels decreased in a dose-dependent manner as the co-treatment concentration increased. $^{**}P < 0.01$, Student's t-test. Mean ± SD, N = 3.

Table S1. List of 1,137 genes significantly changed by more than 2-fold compared to DMSO (0.01%) after treatment with SB590885, SB203580, or a combination of SB590885 and SB203580.

Transcript_ID	Gene_Symbol	Description	Relative expression (SB590885/DMSO)	Relative expression (SB203580/DMSO)	Relative expression (SB590885 and SB203580//DMSO)
NR_037616	BLOC1S5-TXNDC5	BLOC1S5-TXNDC5 readthrough (NMD candidate)	85.893757	163.679350	91.644955
NM_024642,XM_006717287,XM_011519018,XM_011519020,XM_017015133,XM_024447673	GALNT12	polypeptide N-acetylgalactosaminyltransferase 12	4.502653	‘6.361260	12.006776
NM_001040272,NM_052866,XM_011518063,XM_011518064,XM_011518067,XM_011518068,XM_011518070,XM_017015310,XM_017015311,XM_017015312,XM_017015313,XM_017015314	ADAMTSL1	ADAMTS like 1	4.158224	4.955308	11.775178
NM_000759,NM_001178147,NM_172219,NM_172220,NR_033662,NR_168489,NR_168490,NR_168491	CSF3	colony stimulating factor 3	6.097428	3.206643	10.192476
gene-GAPDHP65	GAPDHP65	glyceraldehyde 3 phosphate dehydrogenase pseudogene 65	5.154641	5.174143	7.549555
NM_018905,NM_031495,NM_031496	PCDHA2	protocadherin alpha 2	1.146538	3.543588	7.129500
NM_001270927,NM_001270928,NM_001270929,NM_001270930,NM_001548	IFIT1	interferon induced protein with tetratricopeptide repeats 1	2.046078	4.137138	5.751834

NM_001317748,NM_002380,NM_030583,XM_005250920,XM_017013417,XM_017013418	MATN2	matrilin 2	2.625244	3.093454	5.522047
NR_104644,NR_104645	LINC01204	long intergenic non-protein coding RNA 1204	3.537964	2.577295	5.264127
NM_001185095,NM_001185096,NM_031426,NR_033701,XM_017015177	AIF1L	allograft inflammatory factor 1 like	2.505856	2.989489	5.106882
NM_018929,NM_032407	PCDHGC5	protocadherin gamma subfamily C, 5	2.817928	2.993922	5.083026
NM_030806	C1orf21	chromosome 1 open reading frame 21	2.790781	2.847534	5.034891
NM_002638	PI3	peptidase inhibitor 3	3.447220	2.648829	4.884378
XR_001742893	LOC107986451	uncharacterized LOC107986451	3.375449	2.219219	4.879706
NM_002986	CCL11	C-C motif chemokine ligand 11	1.887300	1.477677	4.531963
XR_001741442	LOC107986196	uncharacterized LOC107986196	2.208780	2.659161	4.525134
NM_001370464,NM_054031	MRGPRX3	MAS related GPR family member X3	3.755928	2.020562	4.330188
NM_001163446,NM_016352	CPA4	carboxypeptidase A4	2.913958	2.177168	4.316677
NR_147886	LOC105371855	uncharacterized LOC105371855	3.420930	2.364220	4.231311
XR_001746819	LOC107987091	uncharacterized LOC107987091	1.922312	2.990410	4.180186
NM_001190737,NM_001190738,NM_001282787,NM_001369458,N	NFIB	nuclear factor I B	1.692685	3.132861	4.115061

M_001369459,NM_001369460,NM_001369461,NM_001369462,NM_001369463,NM_001369464,NM_001369465,NM_001369466,NM_001369467,NM_001369468,NM_001369469,NM_001369470,NM_001369471,NM_001369472,NM_001369473,NM_001369474,NM_001369475,NM_001369476,NM_001369477,NM_001369478,NM_001369479,NM_001369480,NM_001369481,NM_005596,NR_161382,NR_161383,NR_161384,NR_161385,XM_005251467,XM_005251471,XM_006716773,XM_006716774,XM_006716775,XR_001746308,XR_001746309					
NM_015444	TMEM158	transmembrane protein 158	3.787759	1.987423	4.014501
XR_942763,XR_942765,XR_942766,XR_942768,XR_942769	LOC101929004	uncharacterized LOC101929004, transcript variant X4	1.926846	3.788712	3.996982
NR_109971,NR_109972	LINC01483	long intergenic non-protein coding RNA 1483	2.241412	2.112882	3.982501
NR_001458	MIR155HG	MIR155 host gene	2.710502	2.963753	3.930364
NM_033059	KRTAP4-11	keratin associated protein 4-11	2.518757	2.011503	3.920655

NM_001330065,NM_001369869,XM_006723946	KCNE1B	potassium voltage-gated channel subfamily E regulatory subunit 1B	2.273790	2.370708	3.864080
XR_001744422	LOC101928868	uncharacterized LOC101928868	2.081592	1.747554	3.852248
NR_001447	MT1L	metallothionein 1L, pseudogene	3.166209	1.794813	3.798287
NR_026998	LOC91450	uncharacterized LOC91450	1.256258	1.659605	3.781688
NM_001366112,NM_020386,XM_011513034,XM_011513035	PLAAT1	phospholipase A and acyltransferase 1	1.833676	4.595856	3.763826
NM_001040152,NM_001172437,NM_001172438,NM_001184961,NM_001184962,NM_015068	PEG10	paternally expressed 10	2.477521	2.721591	3.729847
gene-LOC100420464	LOC100420464	salvador family WW domain containing protein 1 pseudogene	2.505829	2.447710	3.703384
gene-LOC401218	LOC401218	cyclin Q pseudogene	2.917247	2.103247	3.678977
NM_001199640,NM_001199641,NM_001314044,NM_001314045,NM_001314046,NM_001314047,NM_001314048,NM_001353802,NM_033439,XM_017015285	IL33	interleukin 33	2.605393	1.915231	3.677071
NM_001193532,NM_152304,XM_017000226	RAB42	RAB42, member RAS oncogene family	1.701261	2.424656	3.660150
NM_003495	H4C9	H4 clustered histone 9	3.310443	2.181770	3.648679

NM_001113226,NM_001113228,NM_001312688,NM_001330665,NM_001372166,NM_001372167,NM_001372168,NM_001372169,NM_001372170,NM_001372171,NM_014917,XM_011541025,XM_017000683,XM_017000686	NTNG1	netrin G1	2.078225	2.170353	3.629657
NM_001135585,NM_001328619,NM_001328620,NM_001328621,NM_003039	SLC2A5	solute carrier family 2 member 5	2.810949	2.602905	3.614989
NM_001204824,NM_004519,XM_005250914,XM_006716555,XM_011517026,XM_017013400	KCNQ3	potassium voltage-gated channel subfamily Q member 3	2.267620	2.975473	3.583481
NM_001032409,NM_001320151,NM_002534,NM_016816,XM_006719434,XM_011538413,XM_017019361,XM_017019362,XR_944558	OAS1	2'-5'-oligoadenylate synthetase 1	1.303726	3.811818	3.562105
NM_001253861,NM_032117,NR_045605,XM_005263275,XM_017008688,XM_024454242	MND1	meiotic nuclear divisions 1	1.959177	2.778872	3.550740
NM_033064	ATCAY	ATCAY kinesin light chain interacting caytaxin	1.844921	2.425058	3.545069
NR_037639,NR_037640,NR_037641	MROH7-TTC4	MROH7-TTC4 readthrough (NMD candidate)	6.415432	1.935837	3.541176
NM_207645,XM_011542817,XM_011542818	C11orf87	chromosome 11 open reading frame 87	1.641793	3.664172	3.534198

XR_001738150	LOC102723436	uncharacterized LOC102723436	3.793643	3.219926	3.486984
NM_000930,NM_001319189,NM_033011	PLAT	plasminogen activator, tissue type	3.032321	1.792076	3.481900
NM_001161342,NM_173490,XM_011543156	TMEM171	transmembrane protein 171	2.695501	2.294030	3.470813
NM_002038,NM_022872,NM_022873,XM_024446207	IFI6	interferon alpha inducible protein 6	1.205042	2.711717	3.464878
XR_001739449	LOC107985880	uncharacterized LOC107985880	3.021174	1.261681	3.393275
NM_001870	CPA3	carboxypeptidase A3	1.918897	2.311482	3.390793
NM_054032	MRGPRX4	MAS related GPR family member X4	3.155156	1.489620	3.387989
NM_002463,XM_005260983,XM_005260984,XM_011529571,XM_011529572,XM_011529573,XM_011529574,XM_024452080	MX2	MX dynamin like GTPase 2	1.211801	3.974890	3.372870
NM_001351263,NM_012339,NR_147091,XM_005269666,XM_011539562,XM_011539563,XM_017016010,XR_001747072,XR_001747073,XR_001747074	TSPAN15	tetraspanin 15	1.380201	2.028276	3.334374
NR_134491	LASTR	lncRNA associated with SART3 regulation of splicing	1.744485	2.161675	3.329178
NM_000576,XM_017003988	IL1B	interleukin 1 beta	2.801291	2.358639	3.294660
NM_001142651,NM_001308177,NM_001308178	NEURL1B	neuralized E3 ubiquitin protein ligase 1B	1.726309	2.548620	3.286377

NM_004091,XM_005245748,XM_005245749,XM_011540868,XM_011540870,XM_011540871	E2F2	E2F transcription factor 2	2.491344	2.618777	3.277927
NM_001040624,NM_001040625,NM_001040626,NM_001040627,NM_001040628,NM_001040629,NM_001040630,NM_032041,XM_011517332,XM_011517333,XM_011517334,XM_011517335,XM_017013900,XM_017013901,XM_024447296,XM_024447297,XM_024447298,XM_024447299,XM_024447300,XM_024447301,XM_024447302,XM_024447303,XM_024447304,XR_001745607	NCALD	neurocalcin delta	1.720364	2.229683	3.269492
NM_001127392,NM_013279,XM_005274222,XM_005274223,XM_005274224,XM_005274225,XM_005274226,XM_005274227,XM_005274228,XM_011545234,XM_024448677	MYRF	myelin regulatory factor	2.067131	2.160627	3.245650
NM_001256339,NM_006167,NR_046072	NKX3-1	NK3 homeobox 1	1.886854	2.365169	3.245333
NM_001286673,NM_001286674,NM_001286675,NM_003702,NM_170587,NR_104578,NR_104579	RGS20	regulator of G protein signaling 20	1.841185	2.041989	3.229516

NM_152550,XM_005268380,XM_006714757,XM_011537567,XM_011537568,XM_011537569,XM_017009136	SH3RF2	SH3 domain containing ring finger 2	1.846482	3.226093	3.226010
NM_001270411,NM_004900	APOBEC3B	apolipoprotein B mRNA editing enzyme catalytic subunit 3B	1.948120	2.420516	3.206063
NM_001126181,NM_006176	NRGN	neurogranin	3.054411	2.154406	3.202809
NR_119376	FER1L4	fer-1 like family member 4 (pseudogene)	3.299261	1.608336	3.187249
NM_000676,XM_011523659,XM_011523661,XM_017024197,XR_001752428	ADORA2B	adenosine A2b receptor	2.427562	1.800802	3.182330
NM_001351306,NM_001365862,NM_001371909,NM_153714,XM_011519440,XM_011519441,XM_011519442,XM_011519443,XM_011519445,XM_011519446,XM_017016031,XR_001747077,XR_930487,XR_930488	C10orf67	chromosome 10 open reading frame 67	2.077665	2.463612	3.165072
NM_001034,NM_001165931,NR_164157	RRM2	ribonucleotide reductase regulatory subunit M2	2.431783	2.492638	3.132045
NM_015307,XM_011521407	FAM189A1	family with sequence similarity 189 member A1	1.661486	2.074877	3.131469
gene-CYCSP34	CYCSP34	CYCS pseudogene 34	2.019504	2.110773	3.115948
NM_022120	OXCT2	3-oxoacid CoA-transferase 2	1.463500	2.277779	3.104758

NM_004120	GBP2	guanylate binding protein 2	1.487274	2.765432	3.104511
NM_001184779,NM_001184780,NM_024505,NR_033671,NR_033672	NOX5	NADPH oxidase 5	2.478442	2.034371	3.103827
NM_001197097,NM_001197098,NM_002771,NM_007343,XM_011517965	PRSS3	serine protease 3	2.253468	1.651537	3.090502
NM_005101	ISG15	ISG15 ubiquitin like modifier	1.448540	2.347379	3.078246
NM_000221,NM_006488,XM_005264294,XM_005264296,XM_005264298,XM_006712008,XM_006712009,XM_006712010,XM_006712011,XM_006712012,XM_006712013,XM_006712014,XM_017004060,XM_017004061	KHK	ketohexokinase	1.450074	1.569384	3.074477
NM_002852	PTX3	pentraxin 3	2.677219	1.641172	3.065121
NM_001166293,NM_001166294,NM_001166295,NM_001166417,NM_014021,XM_005270427,XM_005270428,XM_005270429,XM_006710331,XM_011540612,XM_017000234,XM_017000235,XM_017000236,XM_017000237,XM_024453015,XM_024453018,XR_946540	SSX2IP	SSX family member 2 interacting protein	2.645333	1.584902	3.040146

NM_020820,XM_011528934	PREX1	phosphatidylinositol-3,4,5-trisphosphate dependent Rac exchange factor 1	2.206588	1.326855	3.034029
NM_005328	HAS2	hyaluronan synthase 2	2.407590	2.901627	3.016251
NM_133459,XM_017025556,XM_017025557,XM_017025558,XM_024451091,XR_001753142	CCBE1	collagen and calcium binding EGF domains 1	1.935219	2.746255	3.006551
NM_001142556,NM_001142557,NM_012484,NM_012485	HMMR	hyaluronan mediated motility receptor	1.977993	2.892523	2.999623
XR_001741746	LOC107986293	uncharacterized LOC107986293	3.345916	2.935375	2.998768
NM_001282225,NM_001282226,NM_001282227,NM_001282228,NM_001282229,NM_177405,XM_006724080,XM_011546133	ADA2	adenosine deaminase 2	3.083255	2.189537	2.993070
NM_001042403,NM_001278393,NM_001347936,NM_001347937,NM_032147,NR_125360,NR_144944,NR_144945,NR_144946,NR_144947,NR_144948,XM_005269172,XM_005269173,XM_005269174,XM_011538800,XM_011538801,XM_011538802,XM_011538803,XM_011538804,XM_011538805,XM_011538806,XM_017020010,XM_017020012,XR_001748887,XR_944757	USP44	ubiquitin specific peptidase 44	2.207622	2.277296	2.985421

NM_001204414,NM_001204415,NM_022164,XM_005271106,XM_005271107,XM_011541946	TINAGL1	tubulointerstitial nephritis antigen like 1	2.386724	1.850722	2.985188
NR_015384	LOC100126784	uncharacterized LOC100126784	1.799147	1.890088	2.964672
NM_001160354,NM_001160355,NM_017527,XM_005250989,XM_005250990	LY6K	lymphocyte antigen 6 family member K	2.333928	1.670856	2.963126
NM_001370351,NM_001370352,NM_001370353,NM_001370354,NM_006467,XM_011543101,XM_011543102,XM_011543105,XM_017008957	POLR3G	RNA polymerase III subunit G	1.626257	1.739158	2.962448
XR_929046	LOC105375901	uncharacterized LOC105375901	1.470572	2.805949	2.960005
NM_030622,XR_935812	CYP2S1	cytochrome P450 family 2 subfamily S member 1	3.231479	1.380176	2.951177
NM_006101	NDC80	NDC80 kinetochore complex component	1.839871	2.739066	2.946823
NR_038908	SENCR	smooth muscle and endothelial cell enriched migration/differentiation-associated lncRNA	2.356737	1.941834	2.941850
XR_001745706	LOC105379771	uncharacterized LOC105379771	2.812151	1.652413	2.936259
NR_027157	TMPO-AS1	TMPO antisense RNA 1	1.744907	2.758033	2.927153
NM_001195228,NM_019013,XM_017024778	PIMREG	PICALM interacting mitotic regulator	1.658505	2.106486	2.926121

NM_005672,NR_033343	PSCA	prostate stem cell antigen	2.144085	1.981853	2.920418
NM_001184866,NM_001184867,NM_001184870,NM_001184871,NM_001184872,NM_001184873,NM_001366195,NM_001366196,NM_032738,XM_006711581,XM_011510065	FCRLA	Fc receptor like A	1.449641	2.205384	2.917178
NM_001346880,XM_017004063,XM_017004064,XM_017004065,XM_017004066,XM_017004067,XM_017004068,XM_017004069,XM_017004070,XR_001738742	MFSD2B	major facilitator superfamily domain containing 2B	1.923348	1.461022	2.917080
NM_000956	PTGER2	prostaglandin E receptor 2	2.173315	2.275759	2.917041
NR_136175	LINC01960	long intergenic non-protein coding RNA 1960	1.880986	2.024455	2.912063
NR_037194	LINC00664	long intergenic non-protein coding RNA 664	1.922475	2.591488	2.902174
NM_001256063,NM_001256064,NM_001843,NM_175038,XM_005268651,XM_006719241,XM_011537926,XM_011537927,XM_017018826,XM_017018827,XM_024448843,XR_002957288,XR_002957289,XR_002957290,XR_002957291	CNTN1	contactin 1	1.147169	2.893252	2.898127

XR_001753347,XR_935139	LOC105371986	uncharacterized LOC105371986, transcript variant X2	3.255272	2.088703	2.890737
NR_131958,NR_131959	LINC02475	long intergenic non-protein coding RNA 2475	1.581663	2.795059	2.885506
XR_934657	LOC105371722	uncharacterized LOC105371722	2.696636	1.808064	2.877488
NM_004419	DUSP5	dual specificity phosphatase 5	2.497946	1.550316	2.868505
NM_004585	PLAAT4	phospholipase A and acyltransferase 4	1.302977	2.466878	2.860564
NM_001278945,NM_001363040,NM_018492	PBK	PDZ binding kinase	1.669855	2.550015	2.859083
NM_002872,XM_006724286	RAC2	Rac family small GTPase 2	2.044349	1.694379	2.856177
NM_001281443,NM_007000	UPK1A	uroplakin 1A	1.879841	2.427208	2.843367
NM_001100875,NM_001288779,NM_001288780,NM_152598,XM_005257095,XM_005257096,XM_005257097,XM_005257098,XM_005257100,XM_005257102,XM_005257103,XM_005257106,XM_011524435,XM_011524436,XM_011524437,XR_934406	MARCHF10	membrane associated ring-CH-type finger 10	2.338011	1.860584	2.840131
NM_001161575,NM_018296,XM_005256025,XM_005256026,XM_005256027,XM_005256028,XM_011523200,XM_011523201,XM_011523202,XM_011523	LRRC36	leucine rich repeat containing 36	1.656021	1.452856	2.839515

203,XM_011523204,XM_011523205,XM_011523206,XM_017023400,XM_017023401,XM_017023402,XM_024450338,XR_243416,XR_429723					
NM_001248,NM_001291960,NM_001291961,XM_011534266	ENTPD3	ectonucleoside triphosphate diphosphohydrolase 3	1.286965	1.432031	2.834599
NM_001346663,NM_001363848,NM_003258	TK1	thymidine kinase 1	1.770743	2.120053	2.826670
NM_021992	TMSB15A	thymosin beta 15a	2.186411	2.783276	2.822482
NM_001287489,NM_004802,NM_194248,NM_194322,NM_194323	OTOF	otoferlin	1.955884	1.156656	2.816913
id-TRBV12-4	TRBV12-4	T cell receptor beta variable 12-4	2.907440	1.478118	2.808899
NR_135286	LINC00517	long intergenic non-protein coding RNA 517	2.160051	2.230593	2.795453
NM_001146015,NM_014750,XM_017021840	DLGAP5	DLG associated protein 5	1.620987	2.467702	2.784253
XR_951230	FAM247D	family with sequence similarity 247 member D	1.626247	2.725724	2.782108
NM_001317906,NM_001317907,NM_152562,XM_006716294,XM_011544415,XM_011544416,XM_011544417,XM_011544418,XM_024447073,XR_001745479,XR_949377	CDCA2	cell division cycle associated 2	1.803248	2.364010	2.780626

NM_018248,XM_017008360	NEIL3	nei like DNA glycosylase 3	1.992708	2.790680	2.779780
NM_018476	BEX1	brain expressed X-linked 1	1.629883	1.997639	2.774684
NM_001371936,NM_001371938,NM_006072	CCL26	C-C motif chemokine ligand 26	2.140126	1.822917	2.772764
XR_001745011,XR_927202	LOC105375249	uncharacterized LOC105375249, transcript variant X2	1.488199	3.278258	2.769609
NM_015653,XM_005261524,XM_011530126,XM_017028766	RIBC2	RIB43A domain with coiled-coils 2	2.770413	2.262765	2.766962
NM_001199771,NM_002905	RDH5	retinol dehydrogenase 5	1.635087	1.142229	2.756407
id-TRBV12-3	TRBV12-3	T cell receptor beta variable 12-3	2.440508	1.671727	2.754377
NM_001142703,NM_001142704,NM_198947	FAM111B	family with sequence similarity 111 member B	2.480514	2.290559	2.737391
NM_001289987,NM_001300866,NM_015687,NR_110608,XM_005248713,XM_005248715,XM_011535756	FILIP1	filamin A interacting protein 1	1.917174	2.263015	2.732588
NR_033995	LOC389247	uncharacterized LOC389247	2.139968	1.867508	2.732412
NM_001040280,NM_001251901,NM_004233	CD83	CD83 molecule	2.149109	2.023114	2.724306
NM_001017420,XM_011544421,XM_011544422,XR_949378	ESCO2	establishment of sister chromatid cohesion N-acetyltransferase 2	1.951270	2.578749	2.716770
NM_000055,NR_137635,NR_137636	BCHE	butyrylcholinesterase	-1.157101	3.296530	2.716198
NM_001324318,NM_001324319,NM_024745	SHCBP1	SHC binding and spindle associated 1	1.890564	2.467683	2.702185

,NR_136738,XM_011523335					
NR_038285	MELTF-AS1	MELTF antisense RNA 1	1.857753	1.890542	2.700558
NM_031423,NM_145697,XM_011510036,XM_024450112,XM_024450113	NUF2	NUF2 component of NDC80 kinetochore complex	1.881838	2.227086	2.697535
NM_001278208,NM_001363017,NM_007068,NR_103474,XM_011529834,XM_011529835,XM_011529837,XM_011529838,XM_017028542	DMC1	DNA meiotic recombinase 1	2.244208	1.583697	2.693373
NM_001130851,NM_001330173,NM_005192,XM_024449458	CDKN3	cyclin dependent kinase inhibitor 3	1.652259	2.052995	2.685007
NM_001284301,NM_001284302,NM_018685,XM_006715746,XM_006715747,XM_017012354,XM_017012355,XM_017012356	ANLN	anillin actin binding protein	1.783537	2.698333	2.681042
NM_001319077,NM_001319078,NM_001319079,NM_001319080,NM_001319081,NM_001319082,NM_002131,NM_145899,NM_145901,NM_145902,NM_145903,NM_145905	HMGA1	high mobility group AT-hook 1	1.991370	1.561563	2.665987
NM_001363481,NM_145018,XM_011544836,XM_011544837,XM_024448400	DDIAS	DNA damage induced apoptosis suppressor	1.913541	2.790049	2.665098
NM_001255	CDC20	cell division cycle 20	1.634606	2.216634	2.664963

NR_126496,NR_126497,NR_126498,NR_126499,NR_126500	LINC01588	long intergenic non-protein coding RNA 1588	2.151554	1.474194	2.648380
NM_024629,NR_104593,XM_005263218	CENPU	centromere protein U	1.904898	2.099381	2.648092
NM_002178	IGFBP6	insulin like growth factor binding protein 6	1.888745	1.523350	2.645364
NM_031960	KRTAP4-8	keratin associated protein 4-8	1.800535	1.709438	2.645034
NM_006187,XM_005253889,XM_017019363	OAS3	2'-5'-oligoadenylate synthetase 3	1.393402	2.457763	2.639405
NM_001066,XM_011542060,XM_011542063,XM_017002211,XM_017002214,XM_017002215	TNFRSF1B	TNF receptor superfamily member 1B	1.802892	1.701209	2.638649
NM_001285389,NM_001285391,NM_001285392,NM_001285393,NM_001331016,NM_004414,NM_203417,NM_203418,XM_005260929,XM_017028282,XM_017028283	RCAN1	regulator of calcineurin 1	2.193113	1.844347	2.637769
XR_244188	LOC101927486	uncharacterized LOC101927486	1.196210	-1.011767	2.632523
NM_001166691,NM_003318,XM_011536099,XM_011536100,XM_017011242	TTK	TTK protein kinase	1.827532	2.551541	2.613961
NM_001282382,NM_001282383,NM_004219,XM_024446260	PTTG1	PTTG1 regulator of sister chromatid separation, securin	1.683128	2.087775	2.606432
NM_032413,NM_197955	C15orf48	chromosome 15 open reading frame 48	1.948592	1.665068	2.605077

NR_033931	LINC01085	long intergenic non-protein coding RNA 1085	1.459392	2.333184	2.601262
gene-HMGA1P3	HMGA1P3	high mobility group AT-hook 1 pseudogene 3	1.951869	1.498826	2.600648
NM_001244959,NM_001244960,NM_001244961,NM_001244962,NM_001375487,NM_174938,XM_017014588,XM_017014589,XM_024447487,XM_024447488,XM_024447489	FRMD3	FERM domain containing 3	2.423321	1.818053	2.598123
gene-LOC100420528	LOC100420528	CTD nuclear envelope phosphatase 1 pseudogene	3.197325	2.412731	2.597941
NM_001080457,NM_001348568,XM_005259429,XM_006723506,XM_011527520	LRRC4B	leucine rich repeat containing 4B	1.907432	1.878288	2.590372
NM_001204813,NM_002526	NT5E	5'-nucleotidase ecto	2.282294	1.556131	2.589729
NM_001127182,NM_018131,XM_011539918,XM_011539919,XM_011539920,XM_017016372,XM_017016373	CEP55	centrosomal protein 55	1.796081	2.254375	2.588885
NM_000663,NM_001127448,NM_020686,XM_011522400,XM_011522401	ABAT	4-aminobutyrate aminotransferase	1.651724	1.647743	2.588352
XR_924265	LOC105374003	uncharacterized LOC105374003	2.025771	1.478435	2.587992
NM_001009954,NM_017669	ERCC6L	ERCC excision repair 6 like, spindle assembly checkpoint helicase	1.993933	1.935428	2.585356

NM_052941	GBP4	guanylate binding protein 4	1.228924	2.604394	2.581942
NM_001317406,NM_014229,XM_011534033,XM_017007073	SLC6A11	solute carrier family 6 member 11	1.181729	3.431804	2.576925
NM_001376887,NM_003807,NM_172014,XM_017027418,XR_001753777,XR_936212	TNFSF14	TNF superfamily member 14	1.944848	1.302893	2.574964
NM_001114133,NM_024875,XM_005270158,XM_005270159	SYNPO2L	synaptopodin 2 like	1.982985	2.029611	2.573216
NM_001256088,NM_001369160,NM_145013,NR_045767,XM_011542654,XM_011542655	C11orf45	chromosome 11 open reading frame 45	1.564012	2.041781	2.569376
XR_001741759,XR_001741760,XR_001741761,XR_001741762,XR_427567,XR_938965,XR_938966,XR_938969,XR_938970,XR_938971,XR_938972,XR_938974,XR_938975,XR_938976	LOC102723458	uncharacterized LOC102723458, transcript variant X12	1.719874	2.551890	2.564540
XR_950269	LOC105369370	uncharacterized LOC105369370	1.780451	1.520026	2.551970
NM_001304361,NM_152515,NR_130712,XM_011510666	CKAP2L	cytoskeleton associated protein 2 like	1.836012	2.404824	2.550197
NM_002888,NM_206963,XM_005247686,XR_001740228	RARRES1	retinoic acid receptor responder 1	1.809167	2.589138	2.544458
gene-PTGES3P1	PTGES3P1	prostaglandin E synthase 3 pseudogene 1	2.451492	2.036672	2.535824
NM_001134486,NM_052942	GBP5	guanylate binding protein 5	1.912409	2.524232	2.532321

NR_027408	LOC653653	adaptor related protein complex 1 sigma 2 subunit pseudogene	2.129631	5.869134	2.532006
NM_002256	KISS1	KiSS-1 metastasis suppressor	1.805618	2.047998	2.530181
NM_001100620,NM_001278324,NM_005480,XM_006719181,XM_011537723,XM_011537724,XR_944445,XR_944446	TROAP	trophinin associated protein	1.708884	1.904362	2.527258
NM_001039535,NM_145060	SKA1	spindle and kinetochore associated complex subunit 1	1.760587	2.241001	2.525985
gene-NUDCP2	NUDCP2	nuclear distribution C pseudogene 2	4.478792	2.482859	2.524822
XR_001738225,XR_922117	LOC105371427	uncharacterized LOC105371427, transcript variant X2	2.427894	1.944909	2.523977
NM_001077511,NM_001318908,NM_007109	TCF19	transcription factor 19	1.934400	2.243952	2.520324
NM_032270,XM_006710960,XM_011542282,XM_011542283,XM_017002503,XM_017002504	LRRC8C	leucine rich repeat containing 8 VRAC subunit C	2.206976	1.952903	2.518297
NM_001264573,NM_001265577,XM_011524385,XM_011524386,XM_011524387,XM_011524388,XM_011524389,XM_011524390,XM_011524391	KIF18B	kinesin family member 18B	1.707635	2.211433	2.510334
NM_001206942,NM_001206943,NM_001206944,NM_001206945,NM_006574	CSPG5	chondroitin sulfate proteoglycan 5	2.435461	2.650697	2.509379

NM_001310220,NM_001501,NM_178331,NM_178332,XM_017027823	GNRH2	gonadotropin releasing hormone 2	1.068574	1.300366	2.504201
NM_001302826,NM_001302827,NM_016229,NR_126508,XM_005252975,XM_006718251,XM_011520184,XM_011520185,XM_017017920,XM_017017921,XM_017017922,XM_024448580,XM_024448581,XM_024448582,XR_002957151	CYB5R2	cytochrome b5 reductase 2	2.120096	1.724015	2.503759
NM_021649	TICAM2	toll like receptor adaptor molecule 2	1.979686	3.123791	2.501338
XR_001747585	LOC105378482	uncharacterized LOC105378482	2.973845	1.245340	2.500143
gene-MT2P1	MT2P1	metallothionein 2 pseudogene 1	1.846016	1.567771	2.498544
NM_001012270,NM_001012271,NM_001168,XR_243654,XR_934452	BIRC5	baculoviral IAP repeat containing 5	1.552337	2.175857	2.497113
NM_000757,NM_172210,NM_172211,NM_172212,XM_017000369	CSF1	colony stimulating factor 1	1.806844	1.945320	2.495574
NM_001363555,NM_175617	MT1E	metallothionein 1E	1.947694	1.421773	2.494610
NM_001220494,NM_001220496,NM_001354699,NM_080661,XM_011545354,XM_011545355,XM_011545356,XM_011545357,XM_011545358,XM_011545359,XM_024448769	GLYATL1	glycine-N-acyltransferase like 1	2.074572	1.894952	2.493069

NM_144683	DHRS13	dehydrogenase/reductase 13	1.966658	1.901748	2.489621
NM_031942,NM_145810,XR_923034	CDCA7	cell division cycle associated 7	1.922348	2.175299	2.488803
NM_001170406,NM_001170407,NM_001320918,NM_001786,NM_033379,XM_005270303	CDK1	cyclin dependent kinase 1	1.639373	2.419117	2.488058
NM_001330181,NM_001363834,NM_001363835,NM_001363836,NM_138328,XM_006721734,XM_017024273,XM_017024274,XM_017024275,XM_017024276,XM_017024277,XM_017024278,XM_017024279,XM_017024280,XR_001752439	RHBDL3	rhomboid like 3	2.375284	1.550810	2.488021
NM_001114120,NM_017779	DEPDC1	DEP domain containing 1	1.760614	2.457523	2.476981
NR_026964	DKFZP434A062	uncharacterized LOC26102	1.886851	1.824127	2.475646
NM_001281741,NM_001281742,NM_007019,NM_181799,NM_181800,NM_181801,NR_104036,NR_104037	UBE2C	ubiquitin conjugating enzyme E2 C	1.837971	2.108801	2.472458
NM_001321299,NM_032387,XM_017024962,XM_017024966	WNK4	WNK lysine deficient protein kinase 4	2.199425	1.439804	2.472312
NM_005408	CCL13	C-C motif chemokine ligand 13	2.222793	1.714019	2.469198
NM_001195683,NM_001195684,NM_003243	TGFBR3	transforming growth factor beta receptor 3	2.105919	1.497648	2.467912

,NR_036634,XM_006710867					
NM_152565	ATP6V0D2	ATPase H+ transporting V0 subunit d2	2.552938	2.265404	2.467231
NM_001258450,NM_001258451,NM_004203,NM_182687,XM_011522734,XM_011522735,XM_011522736,XM_024450490	PKMYT1	protein kinase, membrane associated tyrosine/threonine 1	1.755742	1.817697	2.463895
NM_001095,NM_001256830,NM_020039,NR_046389,XM_011538350,XM_011538351,XM_011538352	ASIC1	acid sensing ion channel subunit 1	1.346139	1.695580	2.462221
NM_006829	ADIRF	adipogenesis regulatory factor	1.726460	1.069731	2.461520
NR_103553,NR_103554	LIFR-AS1	LIFR antisense RNA 1	1.625019	1.681666	2.458911
NM_004878	PTGES	prostaglandin E synthase	1.729859	1.644680	2.452794
NM_001166017,NM_145061,XM_005266288,XM_011534994	SKA3	spindle and kinetochore associated complex subunit 3	1.923832	2.271398	2.451419
NM_001276451,XM_011540089	DRGX	dorsal root ganglia homeobox	1.915482	1.556954	2.449093
NM_001302123,NM_005409	CXCL11	C-X-C motif chemokine ligand 11	-1.395644	1.618929	2.446400
NM_007248,NM_033629	TREX1	three prime repair exonuclease 1	2.024184	1.998364	2.444919
NM_018176,XM_011513850,XM_017008356	LGI2	leucine rich repeat LGI family member 2	1.604200	2.098383	2.444849
NR_121620,NR_121621	LINC01605	long intergenic non-protein coding RNA 1605	2.026931	1.266657	2.444764

NM_000872,NM_019859,NM_019860,XM_024447973	HTR7	5-hydroxytryptamine receptor 7	1.830256	1.594103	2.443911
NM_001160130,NM_001160132,NM_001160133,NM_001160134,NM_019842,XM_017011058,XM_024446492,XM_024446493	KCNQ5	potassium voltage-gated channel subfamily Q member 5	1.651176	1.613095	2.441495
NM_002531,XM_011528827	NTSR1	neurotensin receptor 1	1.725701	-1.293265	2.439811
NM_130386,XM_011525741	COLEC12	collectin subfamily member 12	2.801552	1.402478	2.439680
NM_001301161,NM_152750,XM_005250224,XM_005250225,XM_006715905,XM_011515955,XM_011515956,XM_011515957,XM_011515958,XM_011515959,XM_017011862,XM_017011863,XM_017011864,XM_017011865,XM_017011866,XM_017011867,XM_024446689,XM_024446690,XR_001744598,XR_001744600,XR_001744601,XR_001744602,XR_001744603,XR_001744604,XR_001744605,XR_001744606,XR_001744607,XR_927414	CDHR3	cadherin related family member 3	1.711390	1.981239	2.434751
NM_001256834,NM_001284526,NM_001313950,NM_001313951,NM_001313952,NM_001313953,NM_0013139	AURKB	aurora kinase B	1.546323	1.933575	2.432148

54,NM_001313955,NM_004217,NR_132730,NR_132731,XM_011524072,XM_017025307,XM_017025308,XM_017025309,XM_017025310,XM_017025311					
NM_014334,XM_011518454	FRRS1L	ferric chelate reductase 1 like	2.033925	2.178276	2.430046
NR_038865	CACTIN-AS1	CACTIN antisense RNA 1	2.843433	1.793168	2.429585
NM_145244	DDIT4L	DNA damage inducible transcript 4 like	2.433240	1.627466	2.428421
NM_001317031,NM_001317032,NM_001317033,NM_182513,XM_005259753,XM_011527702	SPC24	SPC24 component of NDC80 kinetochore complex	1.492596	2.068192	2.426599
XR_001754051,XR_936418,XR_936420,XR_936421,XR_936422,XR_936423,XR_936424	LOC105372321	uncharacterized LOC105372321, transcript variant X5	1.627525	2.182792	2.425273
NR_026825	RPSAP52	ribosomal protein SA pseudogene 52	2.166965	1.648274	2.425002
NM_207341,XM_011544852,XM_011544853	ZP1	zona pellucida glycoprotein 1	2.060579	1.278104	2.424861
NM_000493,XM_006715333,XM_011535432,XM_011535433,XM_017010248	COL10A1	collagen type X alpha 1 chain	2.501163	1.654896	2.424777
NM_001281987,NM_001281988,NM_144569,XM_011542413,XM_011542414,XM_017002779,XM_017002780,XM_017002781,XM_0	SPOCD1	SPOC domain containing 1	1.415518	1.869476	2.422645

17002782,XR_001737533,XR_946797					
NM_001256371,NM_001256372,NM_024680,XM_011520367,XR_930907	E2F8	E2F transcription factor 8	1.873400	2.087402	2.419994
NM_001318521,NM_001318523,NM_006733,XM_005262111,XM_011530894,XM_011530895,XM_011530897,XM_011530899,XM_017029383	CENPI	centromere protein I	1.878589	2.351674	2.417609
NR_073447	LINC00595	long intergenic non-protein coding RNA 595	1.147131	1.923649	2.409576
NM_000015,XM_017012938	NAT2	N-acetyltransferase 2	1.295223	-1.024568	2.409389
NM_001291880,NM_004185,NM_024494	WNT2B	Wnt family member 2B	2.984982	1.461567	2.408822
NM_030928	CDT1	chromatin licensing and DNA replication factor 1	1.705742	1.531050	2.407940
NM_002090	CXCL3	C-X-C motif chemokine ligand 3	1.963512	1.860453	2.406418
NM_001098500,NM_001282767,NM_001282768,NM_001282769,NM_001282770,NM_001321681,NM_019590,XM_005252516,XM_011519552,XM_011519555,XM_011519558,XM_011519559,XM_011519562,XM_011519564,XM_011519565,XM_011519566,XM_017016416,XM_0170164	KIAA1217	KIAA1217	1.740529	1.719297	2.405469

17,XM_017016418,XM_017016419,XM_017016420,XM_017016421,XM_017016422,XM_017016423,XM_017016424,XM_017016425,XM_017016426,XM_017016427,XM_017016429,XM_024448082,XM_024448083,XM_024448084,XM_024448085,XM_024448086					
gene-SUDS3P1	SUDS3P1	SDS3 homolog, SIN3A corepressor complex component pseudogene 1	2.250100	1.768813	2.402044
NM_017414,XM_006724074	USP18	ubiquitin specific peptidase 18	1.323051	2.208229	2.398425
NM_001029860,NR_036491,XM_011516987,XM_011516989,XM_011516990,XM_011516992,XM_017013358,XR_001745524,XR_001745525	FBXO43	F-box protein 43	1.899073	2.943448	2.397104
NM_014373,XM_005247346,XM_005247347,XM_017006161,XM_024453449,XM_024453450,XM_024453451	GPR160	G protein-coupled receptor 160	1.277186	2.516576	2.396265
NM_024734,XM_011537158,XM_011537159,XM_017021646,XM_017021647,XR_001750558,XR_245721,XR_429330,XR_429332	CLMN	calmin	1.312664	1.975769	2.392802
NR_002929	ACTG1P25	actin gamma 1 pseudogene 25	2.341349	2.021731	2.391701

NM_001142548,NM_001370766,NM_003579,XM_011542299,XM_011542300	RAD54L	RAD54 like	1.810764	2.066812	2.388251
NM_012310	KIF4A	kinesin family member 4A	1.550518	2.234055	2.387143
NR_120519,NR_120520	MUC12-AS1	MUC12 antisense RNA 1	1.794071	1.459731	2.385981
NM_001193431,NM_001308297,NM_012411,NM_015967,XM_011541221,XM_011541222,XM_011541223,XM_011541225,XM_017001004,XM_017001005,XM_017001006	PTPN22	protein tyrosine phosphatase non-receptor type 22	1.690605	2.224730	2.384236
NM_022346,NR_073124,XM_011513876,XM_017008543,XM_017008544	NCAPG	non-SMC condensin I complex subunit G	1.835041	2.324731	2.383110
NM_001323517,NM_001323518,NM_006744	RBP4	retinol binding protein 4	1.340173	1.297951	2.381851
NM_001190481,NM_001330490,NM_022111,XM_011541938,XM_011541940,XM_011541941,XR_001737361	CLSPN	claspin	2.101183	2.193370	2.379389
NM_022909	CENPH	centromere protein H	1.590470	2.208366	2.376329
NM_015564	LRRTM2	leucine rich repeat transmembrane neuronal 2	1.222335	2.790783	2.375791
NR_040018,NR_040019	C1QTNF1-AS1	C1QTNF1 antisense RNA 1	2.306054	1.740944	2.374139
NM_001002876,NM_001110215,NM_001304370,NM_001304371,N	CENPM	centromere protein M	1.656303	2.216332	2.368821

M_001304372,NM_001304373,NM_024053,XM_011530368					
NM_001286974,NM_003798,XM_005252291,XM_017015250,XM_017015251,XR_001746410,XR_002956816	CTNNAL1	catenin alpha like 1	2.099745	1.417711	2.368782
NM_001243773,NM_001243774,NM_053064,XM_006720173,XM_011536846,XM_017021376,XM_017021377,XM_024449629,XM_024449630,XM_024449631,XM_024449632,XM_024449633,XM_024449634	GNG2	G protein subunit gamma 2	1.679333	1.753900	2.368045
NM_173564	NYAP1	neuronal tyrosine phosphorylated phosphoinositide-3-kinase adaptor 1	1.453838	1.643395	2.366615
NR_125997	RNF207-AS1	RNF207 antisense RNA 1	1.185337	-1.112552	2.363118
NM_001031850,NM_002782	PSG6	pregnancy specific beta-1-glycoprotein 6	2.031742	1.570305	2.363008
NM_001130862,NM_006479	RAD51AP1	RAD51 associated protein 1	1.891723	2.365233	2.359199
NM_005450	NOG	noggin	2.166301	1.689806	2.356884
NM_018304,XM_024450828	PRR11	proline rich 11	1.718671	2.351335	2.355340
NM_015123,XM_005264722,XM_005264723,XM_017005989,XM_017005990,XM_017005991,XM_017005992,	FRMD4B	FERM domain containing 4B	1.737550	1.655571	2.354960

XM_017005993,XM_017005994,XM_017005995,XM_017005996,XM_017005997					
NM_001145966,NM_002417,XM_006717864,XM_011539818	MKI67	marker of proliferation Ki-67	1.883136	2.413068	2.353780
NM_001143783,NM_001143784,NM_001143785,NM_002005,XM_005254880,XM_005254882,XM_017022005,XM_017022006,XM_017022007,XM_017022008,XM_017022009,XM_017022010,XR_001751142,XR_001751143,XR_001751144,XR_243206	FES	FES proto-oncogene, tyrosine kinase	1.173195	1.424349	2.348494
NM_018154	ASF1B	anti-silencing function 1B histone chaperone	1.957100	1.769432	2.345096
NM_005879,XM_011533264,XM_017005526,XR_001739979	TRAIP	TRAF interacting protein	1.673754	1.935909	2.340623
NM_199420	POLQ	DNA polymerase theta	1.797714	2.252395	2.339589
NM_057749,XM_011517366,XM_017013958,XM_017013959	CCNE2	cyclin E2	2.045811	1.909186	2.335001
NM_004887	CXCL14	C-X-C motif chemokine ligand 14	1.324857	2.276212	2.333065
gene-PEBP1P2	PEBP1P2	phosphatidylethanolamine binding protein 1 pseudogene 2	1.253359	1.704301	2.327786
NM_178229,XM_011509198,XM_011509200,XM_011509201,XM_024453273,XM_02445	IQGAP3	IQ motif containing GTPase activating protein 3	1.525751	2.167420	2.320595

3274,XM_024453275, XM_024453276					
NM_005733	KIF20A	kinesin family member 20A	1.381197	2.511981	2.315231
NR_040582	STAG3L3	stromal antigen 3-like 3 (pseudogene)	1.006200	2.532457	2.311520
NM_030919,XM_017028088	FAM83D	family with sequence similarity 83 member D	1.463889	2.115442	2.310079
NM_001303422,NM_004490,XM_011511022,XM_017003899,XM_024452815,XM_024452816,XR_427085	GRB14	growth factor receptor bound protein 14	1.230695	2.095311	2.309105
XR_001749165	LOC105369783	uncharacterized LOC105369783	1.332520	2.340568	2.304675
NM_005923,XM_011535839,XM_017010870,XM_017010871,XM_017010872,XM_017010873,XM_017010874,XM_017010875,XM_017010876,XM_017010877	MAP3K5	mitogen-activated protein kinase kinase kinase 5	1.855591	1.593382	2.301082
NM_001281710,NM_001281711,NM_001281712,NM_015341,XM_005263908,XM_006712388	NCAPH	non-SMC condensin I complex subunit H	1.563563	1.906267	2.299493
NM_001286229,NM_001286230,NM_016448,XM_011509614	DTL	denticleless E3 ubiquitin protein ligase homolog	1.854827	2.036825	2.298556
NM_001071,NM_001354867,NM_001354868,XM_024451242	TYMS	thymidylate synthetase	1.684928	2.138210	2.297155
NM_001127671,NM_001364297,NM_001364298,NM_002310,XM_	LIFR	LIF receptor subunit alpha	2.118709	1.903212	2.295727

011514042,XM_017009462,XM_017009463					
NM_001281768,NM_021116,XM_005249584,XM_005249585	ADCY1	adenylate cyclase 1	1.633007	1.790363	2.291831
NM_001190799,NM_001190801,NM_014264,XM_005262701,XM_017007662,XM_017007663	PLK4	polo like kinase 4	1.589882	2.504861	2.290010
NM_001032731,NM_002535,NM_016817,XR_001748725	OAS2	2'-5'-oligoadenylate synthetase 2	1.168767	2.713675	2.288620
NM_001024844,NM_002231,XM_006718223,XM_011520067	CD82	CD82 molecule	1.636898	1.558967	2.287552
NM_001254,XM_011525541,XM_011525542	CDC6	cell division cycle 6	1.913011	1.761494	2.284447
NM_001012409,NM_001012410,NM_001012411,NM_001012412,NM_001012413,NM_001199251,NM_001199252,NM_001199253,NM_001199254,NM_001199255,NM_001199256,NM_001199257,NM_138484,NR_131179,NR_131180,XM_011533373,XM_011533375,XM_011533376,XM_011533377	SGO1	shugoshin 1	1.823763	2.436202	2.284090
NM_001039844	ACBD7	acyl-CoA binding domain containing 7	1.421082	1.488058	2.283444
NM_001204182,NM_001204183,NM_002497,XM_005273147	NEK2	NIMA related kinase 2	1.575460	2.519160	2.282551

NM_001010898,XM_006710643	SLC6A17	solute carrier family 6 member 17	1.475062	1.610506	2.280864
NM_006342,XM_005247929,XM_005247930,XM_011513386,XM_017007653	TACC3	transforming acidic coiled-coil containing protein 3	1.548743	1.856055	2.280853
NM_000591,NM_001040021,NM_001174104,NM_001174105	CD14	CD14 molecule	1.427602	1.890990	2.270460
NM_001362884,NM_021020,XM_011544384,XM_011544385,XM_011544386	LZTS1	leucine zipper tumor suppressor 1	1.316106	2.146878	2.270183
NR_135595	ZNF687-AS1	ZNF687 antisense RNA 1	1.057578	-1.280286	2.268943
NM_005946	MT1A	metallothionein 1A	2.131594	1.147876	2.265233
NM_005030	PLK1	polo like kinase 1	1.485739	1.909168	2.264322
NM_001144996,NM_001144997,NM_001367993,NM_001367994,NM_001374465,NM_002206,XM_005268839,XM_005268840,XM_005268841,XM_005268844,XM_005268846,XM_005268848,XM_005268849,XM_005268850,XM_017019265	ITGA7	integrin subunit alpha 7	1.686655	1.411431	2.259942
NM_001308025,NM_152259	TICRR	TOPBP1 interacting checkpoint and replication regulator	1.905167	2.113022	2.257434
XR_937989	FAM247C	family with sequence similarity 247 member C	2.091170	2.942080	2.256771

NR_146531	LNCOG	lncRNA osteogenesis associated	1.989765	1.474368	2.256732
NM_000899,NM_003994	KITLG	KIT ligand	1.898017	1.852147	2.254082
NM_002994	CXCL5	C-X-C motif chemokine ligand 5	1.745581	1.777900	2.253701
NR_102735	DBH-AS1	DBH antisense RNA 1	-1.299408	-1.088207	2.252213
NM_001297655,NM_001297656,NM_001297657,NM_006845,XM_011540540,XM_011540541	KIF2C	kinesin family member 2C	1.465258	2.007647	2.251689
NM_001197330,NM_001197331,NM_001348384,NM_001348385,NM_002692,XM_011536842,XM_011536843,XM_011536844,XM_017021374,XR_001750377,XR_001750378,XR_943479,XR_943480	POLE2	DNA polymerase epsilon 2, accessory subunit	1.419503	1.840615	2.251175
NM_020675,XM_011511516	SPC25	SPC25 component of NDC80 kinetochore complex	1.572258	2.078980	2.251052
NM_001256875,NM_018101	CDCA8	cell division cycle associated 8	1.416266	2.005304	2.250924
NM_001083961,NM_173636,XM_005258809,XM_011526837,XM_011526838,XM_011526839,XM_011526840,XM_011526841,XM_011526842,XM_011526843,XM_011526844,XM_017026665,XR_001	WDR62	WD repeat domain 62	1.653516	1.570711	2.248970

753671,XR_001753672					
NM_005952	MT1X	metallothionein 1X	2.003602	1.271840	2.246468
NM_001211	BUB1B	BUB1 mitotic checkpoint serine/threonine kinase B	1.621361	2.362221	2.244559
XR_001746881,XR_001746882	LOC105376214	uncharacterized LOC105376214, transcript variant X1	1.868578	1.990900	2.241885
NM_012291,XM_006719705,XM_011539024,XM_011539025,XM_017020253,XR_001748927	ESPL1	extra spindle pole bodies like 1, separase	1.523860	1.980444	2.240088
NM_001098484,NM_001134742,NM_003759,XM_011532390,XM_017008792,XM_017008793,XM_024454267,XM_024454268,XM_024454269,XM_024454270,XM_024454271,XM_024454272	SLC4A4	solute carrier family 4 member 4	1.571483	2.283812	2.239477
gene-LOC728688	LOC728688	ubiquitin like with PHD and ring finger domains 1 pseudogene	1.755048	1.967456	2.235900
NM_001010905	C6orf58	chromosome 6 open reading frame 58	1.615615	2.589899	2.232742
NM_016095	GINS2	GINS complex subunit 2	1.623588	1.984242	2.232507
NM_001039775,XM_005245918,XM_011541672,XM_011541673,XR_001737260,XR_946681	CRYBG2	crystallin beta-gamma domain containing 2	1.621810	1.283314	2.231241

XR_936966	LOC105372708	uncharacterized LOC105372708	1.408085	1.316329	2.228273
NM_001190456,NM_001190457,NM_001324014,NM_001324015,NM_006091	CORO2B	coronin 2B	1.784972	1.245263	2.226911
NM_002729	HHEX	hematopoietically expressed homeobox	1.846305	1.991968	2.223561
NM_001349181,NM_001349182,NM_001349183,NM_001349184,NM_001349185,NM_001349186,NM_014746,NR_146069,XM_005246202,XM_017005398,XM_017005400,XM_017005401,XM_017005402,XM_017005403,XR_001739082	RNF144A	ring finger protein 144A	2.666391	2.878095	2.223487
NM_024081,XM_006718313,XM_006718314	PRRG4	proline rich and Gla domain 4	1.880147	1.800252	2.214924
NM_001042426,NM_001809	CENPA	centromere protein A	1.400311	2.141924	2.213358
NM_000204,NM_001318057,NM_001331035,NM_001375278,NM_001375279,NM_001375280,NM_001375281,NM_001375282,NM_001375283,NM_001375284,NR_164671,NR_164672,NR_164673,XM_006714210,XM_011531920,XM_017008166	CFI	complement factor I	2.273348	1.874365	2.212275
NM_001282460,XM_011534989,XM_011534990,XM_011534992,XM_011534993,XM_01	LRRC63	leucine rich repeat containing 63	1.062788	2.110667	2.208959

7020421,XM_017020422,XM_017020423,XM_017020424,XM_017020425,XM_017020426,XM_017020427,XM_017020428,XM_017020429,XM_017020430,XM_017020431,XR_001749492,XR_001749493,XR_001749494,XR_001749496,XR_001749497,XR_001749498,XR_941517,XR_941518,XR_941519,XR_941520					
NM_002275,XM_011524784,XM_017024614	KRT15	keratin 15	1.206726	1.384834	2.203564
NR_110826	LINC02015	long intergenic non-protein coding RNA 2015	2.199736	1.604910	2.199721
NM_018159	NUDT11	nudix hydrolase 11	1.441491	1.791271	2.198564
NM_001145208,NM_018369,XM_011543509,XM_011543510,XM_017009629,XR_948274	DEPDC1B	DEP domain containing 1B	2.122409	2.644889	2.196885
NM_001258357,NM_001258358,NM_001258359,NM_002149,NM_134421,XM_005246160,XM_005246161,XM_005246162,XM_005246163,XM_011510346,XM_011510347,XM_011510348,XM_011510349,XM_017003950,XM_017003951,XM_0	HPCAL1	hippocalcin like 1	1.850555	1.328393	2.190063

17003952,XM_017003953					
NM_080668,XM_005273733,XM_005273734,XM_011544743,XM_011544747,XM_011544748,XM_011544749,XM_017017158,XR_001747757,XR_001747758	CDCA5	cell division cycle associated 5	1.630157	1.813264	2.189944
NM_002263,XM_011514585,XM_011514587,XM_017010836,XM_017010837	KIFC1	kinesin family member C1	1.692973	1.869329	2.186915
NM_018518,NM_182751,XM_011519538	MCM10	minichromosome maintenance 10 replication initiation factor	1.912029	1.956413	2.186072
NR_103778,NR_103779	FLG-AS1	FLG antisense RNA 1	1.366806	1.772956	2.178321
NM_001301339,NM_213720,NR_125755,NR_125756	CHCHD10	coiled-coil-helix-coiled-coil-helix domain containing 10	1.893886	1.254291	2.176467
NM_003206,NM_198392	TCF21	transcription factor 21	1.679038	1.280537	2.176190
NM_001142935,NM_031300	MXD3	MAX dimerization protein 3	1.584471	1.816389	2.176100
NM_001007255,NM_152366,NR_033385,NR_033386	KLHDC9	kelch domain containing 9	1.427685	1.909100	2.173330
NM_001243142,NM_001243143,NM_001243144,NM_001301136,NM_016359,NM_018454,XM_005254428,XM_005254430,XM_005254431,XM_006720559,XM_006720560,XM_	NUSAP1	nucleolar and spindle associated protein 1	1.668080	2.187690	2.173111

006720561,XM_006720562,XM_006720563,XM_017022294,XM_017022295,XR_001751299,XR_001751300,XR_001751301					
NR_145451	LINC02012	long intergenic non-protein coding RNA 2012	1.451137	1.343569	2.168633
XR_001755593	LOC105373077	uncharacterized LOC105373077	2.160475	1.394491	2.164111
NM_001031683,NM_001289758,NM_001289759,NM_001549	IFIT3	interferon induced protein with tetratricopeptide repeats 3	1.240559	1.946402	2.162224
NM_001067,XM_005257632,XM_011525165	TOP2A	DNA topoisomerase II alpha	1.627128	2.333946	2.161514
NR_026934	LINC02085	long intergenic non-protein coding RNA 2085	1.797148	1.737471	2.160982
NM_001029989,NM_014736,NR_109934	PCLAF	PCNA clamp associated factor	1.641546	2.015766	2.158835
NM_178171,XM_006721832,XM_011524651,XM_017024502,XM_017024503,XM_017024504	GSDMA	gasdermin A	1.412193	1.479550	2.157765
NM_000418,NM_001257406,NM_001257407,NM_001257997,XM_005255308,XM_006721043,XM_011545825,XM_011545826,XM_011545827,XM_011545828,XM_011545829,XM_011545830,XM_011545831,XM_011545832,XM_011545833,X	IL4R	interleukin 4 receptor	1.740524	1.459038	2.153559

M_011545834,XM_017023211					
NM_007193,XM_011531571	ANXA10	annexin A10	2.551070	1.156699	2.152414
NM_033184	KRTAP2-4	keratin associated protein 2-4	1.585117	1.350878	2.150703
NM_002232,NR_109845,NR_109846	KCNA3	potassium voltage-gated channel subfamily A member 3	2.321011	2.674936	2.149453
NM_001185056,NM_005602	CLDN11	claudin 11	1.861752	1.506114	2.149102
NM_019886	CHST7	carbohydrate sulfotransferase 7	1.940634	1.282908	2.148838
NM_001170806,NM_173549	ERICH5	glutamate rich 5	1.362696	1.857404	2.147329
XR_001754063,XR_001754064,XR_001754065,XR_001754066,XR_001754067,XR_001754068,XR_936406	LOC105372310	uncharacterized LOC105372310, transcript variant X6	1.516122	2.098548	2.145360
NM_001085480	FAM162B	family with sequence similarity 162 member B	1.677005	1.192505	2.138333
NM_002596,NM_212502,NM_212503,XM_011509602,XM_017001423,XR_001737212,XR_001737213,XR_002956776,XR_426779,XR_426780,XR_426781,XR_426782,XR_426784,XR_921822	CDK18	cyclin dependent kinase 18	1.510787	1.436756	2.136613
gene-LOC339966	LOC339966	ubiquitin family domain containing 1 pseudogene	3.580067	1.843120	2.135890

NM_001267580,NM_003981,NM_199413,XM_005254987,XM_006720759,XM_006720760,XM_011522187,XM_011522188,XM_011522189,XM_011522190,XM_011522191,XM_011522192,XM_017022712,XM_017022713,XM_017022714,XM_017022715,XM_017022716,XM_017022717	PRC1	protein regulator of cytokinesis 1	1.504646	1.999976	2.135830
NM_001278616,NM_001278617,NM_004336,XR_923001	BUB1	BUB1 mitotic checkpoint serine/threonine kinase	1.464054	1.942029	2.134614
NM_001351678,NM_152476,XM_011527696,XM_011527697,XM_017026327,XM_017026328,XM_017026329,XM_017026330,XM_024451380	ZNF560	zinc finger protein 560	1.588451	1.444993	2.133864
NR_026815	TMEM191A	transmembrane protein 191A (pseudogene)	1.723746	1.580749	2.129681
NM_000882,NM_001354582,NM_001354583	IL12A	interleukin 12A	1.552006	2.007635	2.124046
NM_052969	RPL39L	ribosomal protein L39 like	1.324833	1.751926	2.120800
XR_940324	LOC105374848	uncharacterized LOC105374848	1.495412	1.672911	2.117256
NM_001256944,NM_001830	CLCN4	chloride voltage-gated channel 4	1.479732	2.098847	2.114308
NM_001367886,NM_207311,NR_147892,NR_147893,NR_147894,	BICDL1	BICD family like cargo adaptor 1	1.515208	1.240768	2.112491

XM_006719694,XM_006719696,XM_006719697,XM_011538999,XM_011539000,XM_011539001,XR_001748923,XR_944834,XR_944836					
NM_032336,XM_005273659	GINS4	GINS complex subunit 4	2.011661	1.774898	2.107417
NM_001185015,NM_001378442,NM_001378443,NM_001378444,NM_001378445,NM_001378446,NM_001378447,NM_004509,NM_004510,NM_080424,XM_011511090,XM_011511091,XM_011511092,XM_017003968,XM_017003969,XM_024452850	SP110	SP110 nuclear body protein	1.223359	1.751419	2.106807
NM_001305792,NM_014875,XM_011510231,XM_011510232,XM_011510233,XM_011510235,XM_011510236,XM_017003005,XM_017003006,XM_017003007	KIF14	kinesin family member 14	1.675012	2.452362	2.106173
NM_001039656,NM_004923,XM_011545402,XM_011545403,XM_017018588,XR_950115	TESMIN	testis expressed metallothionein like protein	1.678757	1.732908	2.105589
NR_037853	ATP6V1G2-DDX39B	ATP6V1G2-DDX39B readthrough (NMD candidate)	-1.315983	-1.111213	2.104907

NM_016426,XM_005261627,XM_011530213,XM_017028816	GTSE1	G2 and S-phase expressed 1	1.431812	1.795844	2.103362
NM_001199864	BCL2L2-PABPN1	BCL2L2-PABPN1 readthrough	1.661376	1.649863	2.102867
NM_001330430,NM_020485,NM_138616,NM_138617,NM_138618,XM_005245957,XM_006710810,XM_011541888,XM_011541889,XM_011541891,XM_017002014	RHCE	Rh blood group CcEe antigens	1.638733	1.275111	2.101558
NM_006668,XM_005267274,XM_011536364,XM_011536365,XM_017020933	CYP46A1	cytochrome P450 family 46 subfamily A member 1	1.958276	1.420375	2.099606
NM_001297602,NM_001297603,NM_001297604,NM_001331019,NM_031299,NR_123727,NR_138531,XR_001748879,XR_001748880,XR_001748881,XR_002957383,XR_002957384,XR_002957385	CDCA3	cell division cycle associated 3	1.447466	1.880855	2.097527
NM_001166260,NM_004237,XM_011514163	TRIP13	thyroid hormone receptor interactor 13	1.454497	1.914275	2.093871
NM_001278610,NM_002466	MYBL2	MYB proto-oncogene like 2	1.549263	2.048243	2.091630
NM_015714	G0S2	G0/G1 switch 2	1.644939	1.510277	2.089138
NM_001289067,NM_001289068,NM_001289069,NM_001289070,NM_001289071,NM_001289072,NM_001289073,NM_001289074,N	HELLS	helicase, lymphoid specific	1.657683	1.885409	2.088563

M_001289075,NM_018063,XM_024447967,XM_024447968					
NM_001282962,NM_001282963,NM_018410,XM_011511437	HJURP	Holliday junction recognition protein	1.493146	1.945355	2.088191
NM_001174159,NM_001174160,NM_001363110,NM_001363111,NM_022071,NR_156444	SH2D4A	SH2 domain containing 4A	1.360952	1.869286	2.087383
NM_001289973,NM_207411,XM_011534750,XM_017013401,XM_017013402	XKR5	XK related 5	-1.038357	2.205450	2.086971
NM_153221	CILP2	cartilage intermediate layer protein 2	1.602371	-1.177936	2.085369
NM_004482,XM_005246449,XM_011510929,XM_017003770,XR_002959253	GALNT3	polypeptide N-acetylgalactosaminyltransferase 3	1.629122	1.672976	2.085279
NM_001145664,NM_001367508,NM_001367509,NM_001367510,XM_011511771,XM_011511772,XM_011511773,XM_011511776,XM_011511777,XM_011511778,XM_017004851,XM_017004852,XM_017004853,XM_017004854,XR_001738924	RFX8	RFX family member 8, lacking RFX DNA binding domain	1.454516	1.479282	2.082836
NR_022014	HMGN2P46	high mobility group nucleosomal binding domain 2 pseudogene 46	1.251566	1.102179	2.082354
NM_001243088,NM_001243089,NM_021953,NM_202002,NM_202	FOXM1	forkhead box M1	1.494731	1.904338	2.081843

003,XM_005253676,XM_011520930,XM_011520931,XM_011520932,XM_011520933,XM_011520934,XM_011520935,XR_931507					
NM_001166131,NM_152463,XM_005257081,XM_011524395,XM_017024236,XR_002957972,XR_934390	EME1	essential meiotic structure-specific endonuclease 1	1.669408	1.873271	2.081393
NM_005225	E2F1	E2F transcription factor 1	1.643901	1.586563	2.078167
NM_005929,NM_033316,XM_006713643,XM_011512850,XM_011512851	MELTF	melanotransferrin	1.546792	1.611308	2.077697
NM_000775,NR_134981,NR_134982	CYP2J2	cytochrome P450 family 2 subfamily J member 2	1.056514	1.561110	2.077546
NM_001144925,NM_001178046,NM_001282920,NM_002462,XM_005260978,XM_005260979,XM_005260980,XM_005260981,XM_005260982,XM_011529568,XM_011529570,XM_017028349,XM_017028350,XM_017028351,XM_017028352	MX1	MX dynamin like GTPase 1	1.004911	2.141058	2.075268
NM_001040059,NM_001251	CD68	CD68 molecule	1.694724	1.336254	2.072476
NR_040288,NR_040289	AKR7L	aldo-keto reductase family 7 like (gene/pseudogene)	1.699145	1.340155	2.071226
NM_001161440,NM_002842,XM_011527183,XM_011527188,XM_	PTPRH	protein tyrosine phosphatase receptor type H	2.007699	1.279472	2.070509

011527190,XM_017027056,XM_017027057,XM_017027058,XM_017027059,XM_017027060,XM_017027061,XM_017027062,XM_017027063,XM_017027064,XR_001753730,XR_001753731					
NM_001009991,NM_001242384,NM_001242394,NM_001242395,NM_001318745,XM_005267215,XM_005267218,XM_005267222,XM_006715605,XM_006715606,XM_006715611,XM_011536254,XM_011536255,XM_017011495,XM_017011496,XM_017011497,XM_017011498,XM_017011499,XM_024446588	SYTL3	synaptotagmin like 3	1.800655	1.443512	2.067906
NM_001005413,NM_007057,NM_032997,XM_017015605,XM_024447784,XR_428692	ZWINT	ZW10 interacting kinetochore protein	1.615916	1.727784	2.066330
NM_001257135,NM_002309,XM_024452239,XM_024452240	LIF	LIF interleukin 6 family cytokine	1.637890	1.555662	2.062617
NM_001099640,NM_018286,XM_017024815,XM_017024816,XM_024450826	TMEM100	transmembrane protein 100	3.310120	-1.176706	2.058118
NM_001111031,NM_001111032,NM_001111033,NM_145259	ACVR1C	activin A receptor type 1C	1.478267	2.133429	2.053467

NM_001547	IFIT2	interferon induced protein with tetratricopeptide repeats 2	1.325534	1.563978	2.052038
NM_000161,NM_001024024,NM_001024070,NM_001024071,XM_017021218	GCH1	GTP cyclohydrolase 1	1.575486	1.381076	2.050043
NR_135038,NR_147930,NR_147931,NR_147932	LINC02609	long intergenic non-protein coding RNA 2609	1.362913	2.232745	2.049525
XR_001741915,XR_001741916	LOC107986325	uncharacterized LOC107986325, transcript variant X2	1.484195	1.754660	2.048630
NM_005953	MT2A	metallothionein 2A	1.590402	1.395888	2.048043
NM_001031739,NM_001168530,NM_001168531,NM_024087,XM_005274446,XM_011545458,XM_011545460,XM_017029283,XM_017029284,XM_017029285,XM_017029286,XM_024452341	ASB9	ankyrin repeat and SOCS box containing 9	1.296663	1.808097	2.046694
NM_002970,NR_027783,XM_024452421	SAT1	spermidine/spermine N1-acetyltransferase 1	1.885076	1.308372	2.046693
NM_000057,NM_001287246,NM_001287247,NM_001287248,XM_006720632,XM_011521881,XM_011521882	BLM	BLM RecQ like helicase	2.059117	2.322341	2.046652
NM_001193333,NM_007074,XM_011545714,XM_017022885,XM_017022886	CORO1A	coronin 1A	1.089284	1.484742	2.044508
NM_001282062,NM_001282063,NM_001282068,NM_003586,NR_1	DOC2A	double C2 domain alpha	1.526178	1.740790	2.043519

04089,NR_104090,XM_011545975,XM_017023778,XM_024450472,XM_024450473,XM_024450474					
NM_001103160,NM_001103161,XM_011541459,XM_011541460,XM_011541461,XM_011541462	SH2D5	SH2 domain containing 5	1.551602	1.394116	2.042146
NM_001100118,NM_001100119,NM_001371229,NM_001371231,NM_001371232,NM_005432,XM_005268046	XRCC3	X-ray repair cross complementing 3	1.686066	1.651371	2.038449
NM_000062,NM_001032295	SERPING1	serpin family G member 1	1.596006	1.587126	2.036370
NM_001143839,NM_001146209,NM_001243784,NM_002599,XM_005274040	PDE2A	phosphodiesterase 2A	1.404763	2.610643	2.029917
NM_138444	KCTD12	potassium channel tetramerization domain containing 12	1.499431	1.517661	2.028948
NM_001135099,NM_001382720,NM_005656	TMPRSS2	transmembrane serine protease 2	2.655347	-1.027092	2.027140
NM_001171251,NM_001321310,NM_001321311,NM_024032,XM_011525187,XM_011525188,XM_011525189,XM_011525190,XM_011525191,XM_011525192,XM_011525193,XM_011525194,XM_011525195,XM_011525197,XR_002958064,XR_934545	HROB	homologous recombination factor with OB-fold	1.609345	1.676725	2.026023

NM_006397	RNASEH2A	ribonuclease H2 subunit A	1.679779	1.644107	2.024541
NM_015180,NM_182910,NM_182913,NM_182914,XM_005267454,XM_005267456,XM_005267457,XM_005267458,XM_005267459,XM_011536574,XM_011536575,XM_011536576,XM_011536577,XM_011536578,XM_011536579,XM_011536580,XM_011536581,XM_011536582,XM_011536584,XM_017021101,XM_017021102,XM_017021103,XM_017021104,XR_001750198	SYNE2	spectrin repeat containing nuclear envelope protein 2	1.870577	2.202899	2.024155
NM_001317931,NM_003116,XM_005260520,XM_011529009	SPAG4	sperm associated antigen 4	2.080510	1.357401	2.020545
NM_153695,XM_011518345	ZNF367	zinc finger protein 367	1.678318	1.844199	2.018315
NM_001005376,NM_001005377,NM_001301037,NM_002659,XM_005258990,XM_011527027,XM_011527028,XM_011527029,XM_011527030,XM_011527031,XM_017026872,XM_017026873	PLAUR	plasminogen activator, urokinase receptor	1.666143	1.312486	2.017511
NM_001165,NM_182962,XM_024448467	BIRC3	baculoviral IAP repeat containing 3	1.476678	1.863286	2.016600
NM_000602	SERPINE1	serpin family E member 1	1.714958	1.629244	2.015916

NM_001145775,NM_001145776,NM_001145777,NM_004117	FKBP5	FKBP prolyl isomerase 5	1.740076	1.838249	2.015729
NM_012306,XM_005268730	FAIM2	Fas apoptotic inhibitory molecule 2	-1.159862	1.407589	2.014370
NM_001206615,NM_001206616,NM_001378041,NM_001378042,NM_001378043,NM_001378044,NM_001378045,NM_001378046,NM_001378047,NM_001378048,NM_001378049,NM_001378050,NM_001378051,NM_001378052,NM_001378053,NM_001378054,NM_001378055,NM_001378056,NM_012153,NR_165390,NR_165391,XM_005252861,XM_024448431,XM_024448434	EHF	ETS homologous factor	2.158460	1.554387	2.014020
NM_020439,XM_017001866,XM_017001867	CAMK1G	calcium/calmodulin dependent protein kinase IG	1.555707	1.394902	2.013426
NM_001136023,NM_001261461,NM_006163	NFE2	nuclear factor, erythroid 2	1.789468	1.064561	2.012763
NM_001003827,NM_021616,NM_130390	TRIM34	tripartite motif containing 34	1.259861	1.865182	2.012148
NM_004051,NM_203314,NM_203315,XM_005269352,XM_005269355,XM_011513067,XM_017007007,XM_017007008,XM_017007009,XM_017007010,XM_017007011,XM_01	BDH1	3-hydroxybutyrate dehydrogenase 1	1.606770	1.206044	2.010148

7007012,XM_017007013,XM_017007015,XR_001740229					
NM_001130688,NM_001130689,NM_002129	HMGB2	high mobility group box 2	1.381357	1.726264	2.009534
NM_001284511,NM_001284512,NM_001284513,NM_001324118,NM_152341	PAQR4	progestin and adipoQ receptor family member 4	1.661425	1.570045	2.007528
NM_005167	PPM1J	protein phosphatase, Mg2+/Mn2+ dependent 1J	1.256537	1.110608	2.004187
NM_000405,NM_001167607	GM2A	GM2 ganglioside activator	1.814117	1.367556	2.003848
NM_001355404,NM_001355405,NM_001355406,XM_024451520,XM_024451521	ZNF724	zinc finger protein 724	1.920267	1.900631	2.003396
NM_152446,NR_157142,XM_011536490,XM_011536491,XM_011536492,XM_011536493,XM_011536495,XM_017021042,XM_017021043	CEP128	centrosomal protein 128	1.586211	1.780606	2.003317
NM_001017523,NM_001018072,NM_001347943,NM_001347944,XM_005268645,XM_011537909,XM_011537910,XM_017018807	BTBD11	BTB domain containing 11	1.425710	1.353391	2.002882
NM_001319224,NM_003686,NM_006027,NM_130398,XM_006711840,XM_011544321,XM_011544322,XM_011544323,XM_011544324,XM_011544325,XM_017002793	EXO1	exonuclease 1	1.604738	1.850473	2.002307

NR_131982	LOC105373383	uncharacterized LOC105373383	1.347577	1.770774	2.001303
NM_001128850,NM_004165	RRAD	RRAD, Ras related glycolysis inhibitor and calcium channel regulator	1.394604	1.345579	2.000762
NM_001165252	KRTAP2-3	keratin associated protein 2-3	1.673780	1.568805	2.000589
NR_015421	LOC154761	family with sequence similarity 115, member C pseudogene	1.851688	1.603019	2.000473
NM_004523	KIF11	kinesin family member 11	1.387504	2.090627	1.989806
NM_001284259,NM_001382506,NM_016195,NR_168374,NR_168375	KIF20B	kinesin family member 20B	1.661862	2.186363	1.983024
NM_000222,NM_001093772,XM_005265740,XM_005265741,XM_005265742,XM_017008178,XM_017008179,XM_017008180	KIT	KIT proto-oncogene, receptor tyrosine kinase	1.363217	2.227476	1.979093
XM_017029164	USP41	ubiquitin specific peptidase 41	1.131745	2.157188	1.978191
NM_022842,NM_178181,XM_011534024,XM_017007070,XM_024453718	CDCP1	CUB domain containing protein 1	2.266019	1.308380	1.976648
NM_001167911,NM_001167912,NM_001167915,NM_001167916,NM_001167917,NM_024621,XM_011513134,XM_011513135,XM_024453746,XM_024453747,XM_024453748,X	VEPH1	ventricular zone expressed PH domain containing 1	2.036314	1.454328	1.967192

M_024453749,XM_024453750					
NM_001270989,NM_001270990,NM_001270991,NM_001270992,NM_001270993,NR_073114,NR_073117,XM_011531817	EPGN	epithelial mitogen	3.457380	1.149819	1.965722
NR_015431,NR_024153,NR_034120,NR_109850,NR_109851,NR_109852,NR_109853,NR_109854,NR_109855,NR_110472,NR_110473	LINC-PINT	long intergenic non-protein coding RNA, p53 induced transcript	2.089049	1.074561	1.965683
NM_003155	STC1	stanniocalcin 1	2.721799	1.270987	1.961156
NM_001010919,NM_001276460,XM_005266999,XM_011535845	CALHM6	calcium homeostasis modulator family member 6	1.792206	2.343836	1.960079
NM_001256850,NM_001267550,NM_003319,NM_133378,NM_133379,NM_133432,NM_133437,XM_017004819,XM_017004820,XM_017004821,XM_017004822,XM_017004823,XM_024453094,XM_024453095,XM_024453096,XM_024453097,XM_024453098,XM_024453099,XM_024453100	TTN	titin	2.165772	1.907325	1.957012
NM_001317860,NM_007280	OIP5	Opa interacting protein 5	1.571713	2.412350	1.948652

gene-RPLP1P4	RPLP1P4	ribosomal protein lateral stalk subunit P1 pseudogene 4	2.039853	1.694302	1.939661
NM_001004339,NM_001307931,XM_011541480,XM_011541481,XM_011541482,XM_011541483,XM_011541484,XM_011541485,XM_011541486,XM_011541487,XM_011541488	ZYG11A	zyg-11 family member A, cell cycle regulator	1.355508	2.021571	1.934857
NM_001198557,NM_005573,NR_134488	LMNB1	lamin B1	1.365069	2.016611	1.931215
gene-LOC729217	LOC729217	EBP like pseudogene	3.029085	1.406387	1.920033
NM_003979	GPRC5A	G protein-coupled receptor class C group 5 member A	2.068298	1.166510	1.918543
NM_016343,XM_011509082,XM_017000086	CENPF	centromere protein F	1.590984	2.114540	1.911577
NM_001237	CCNA2	cyclin A2	1.208411	2.037114	1.908035
NM_001277403,XM_017026114,XM_017026115,XR_001753564,XR_001753565,XR_001753566,XR_001753567,XR_001753568,XR_001753569	ZNF730	zinc finger protein 730	1.683708	2.110510	1.900869
NR_002557	LOC613038	SAGA complex associated factor 29 pseudogene	2.166939	1.509452	1.900829
NM_001144994	C2orf72	chromosome 2 open reading frame 72	1.882315	2.158846	1.892828
NM_144508,NM_170589,XM_017022432	KNL1	kinetochore scaffold 1	1.566568	2.229929	1.886811

NM_001282695,NM_001282696,NM_001282697,NM_001282698,NM_001282699,NM_001282700,NM_001282701,NM_001282702,NM_001282703,NM_001320735,NM_001320736,NM_001320737,NM_001320738,NM_001320739,NM_001320740,NM_001320741,NM_031453,XM_005252616,XM_011519735,XM_017016749	FAM107B	family with sequence similarity 107 member B	2.032903	1.400196	1.863438
NR_002835	HAS2-AS1	HAS2 antisense RNA 1	1.680890	2.301301	1.849447
NM_005127	CLEC2B	C-type lectin domain family 2 member B	2.215737	1.505204	1.834199
NM_006273	CCL7	C-C motif chemokine ligand 7	2.254943	1.065174	1.831188
NM_001206846,NM_018136	ASPM	assembly factor for spindle microtubules	1.461212	2.147954	1.823618
NM_001329993,NM_001329994,NM_001381946,NM_181791,XM_011515374,XM_011515375,XM_011515383,XM_011515385,XM_011515386,XM_011515388,XM_011515389,XM_017012161,XM_017012163,XM_017012164,XM_017012165,XM_017012166	GPR141	G protein-coupled receptor 141	2.114206	1.347376	1.812248
NR_033851	SERPINB9P1	serpin family B member 9 pseudogene 1	2.114589	2.274717	1.806708

NM_001289112,NM_130808,NM_153429,XM_011512406,XM_011512407,XM_011512408,XM_017005694,XM_017005695,XM_017005696,XM_024453338,XM_024453339,XM_024453340	CPNE4	copine 4	1.246903	2.158215	1.798645
NM_001099677,NM_020844,XM_005273584,XM_005273585,XM_005273586,XM_005273588,XM_005273590,XM_005273591,XM_011544596,XM_011544597,XM_011544598,XM_011544600,XM_011544601,XM_017013705,XM_017013706,XM_017013710,XM_017013714,XM_024447214,XM_024447215,XM_024447216,XM_024447217,XM_024447218,XM_024447219	TRMT9B	tRNA methyltransferase 9B (putative)	1.119870	2.294752	1.793094
NM_153366	SVEP1	sushi, von Willebrand factor type A, EGF and pentraxin domain containing 1	2.000595	1.288921	1.777904
NM_019018,XM_011514056,XR_925623	OTULINL	OTU deubiquitinase with linear linkage specificity like	2.268477	1.118538	1.776241
NM_199001	CYSRT1	cysteine rich tail 1	2.042341	1.847218	1.758727
gene-RPS15AP19	RPS15AP19	ribosomal protein S15a pseudogene 19	2.093076	1.520853	1.742617

XR_946104	LOC105378383	uncharacterized LOC105378383	2.179981	1.891751	1.720295
NM_001365758,NM_001370326,NM_153228,XM_006721728,XM_011524428,XM_011524429,XM_011524430,XM_011524431,XM_011524433,XM_011524434,XM_017024263,XM_017024264,XM_017024265,XM_017024266,XM_017024267,XM_017024269,XM_017024270	ANKFN1	ankyrin repeat and fibronectin type III domain containing 1	2.881744	3.063275	1.716396
NM_001207035,NM_001207036,NM_001207037,NM_001207038,NM_001207039,NM_001207040,NM_001207041,NM_016135,XM_011514659	ETV7	ETS variant transcription factor 7	-1.345950	2.236827	1.715182
gene-LDHAP4	LDHAP4	lactate dehydrogenase A pseudogene 4	2.459258	1.677603	1.708113
gene-CLIC1P1	CLIC1P1	chloride intracellular channel 1 pseudogene 1	2.074806	1.616647	1.702645
NM_001003679,NM_001003680,NM_001198687,NM_001198688,NM_001198689,NM_002303	LEPR	leptin receptor	2.203856	1.076168	1.692248
XR_001741057	LINC02051	long intergenic non-protein coding RNA 2051	-1.364207	3.277746	1.629814
NM_004915,NM_016818,NM_207174,NM_207627,NM_207628,NM_207629,XM_01152	ABCG1	ATP binding cassette subfamily G member 1	2.042012	1.009405	1.620141

9806,XM_011529807,XM_024452141					
NM_000832,NM_001185090,NM_001185091,NM_007327,NM_021569,XM_005266071,XM_005266072,XM_005266073,XM_011518583	GRIN1	glutamate ionotropic receptor NMDA type subunit 1	2.212686	-1.163975	1.615601
NM_016354,XM_005260203,XM_011528792,XM_017027826,XM_017027827,XR_001754249,XR_001754250,XR_001754251,XR_001754252,XR_001754253,XR_001754254,XR_001754255,XR_001754256,XR_001754257,XR_001754258,XR_002958473,XR_002958474,XR_244115,XR_244116,XR_936524	SLCO4A1	solute carrier organic anion transporter family member 4A1	2.138934	-1.050881	1.595337
XR_001745952,XR_428359,XR_929012,XR_929013,XR_929014	LOC102724687	uncharacterized LOC102724687, transcript variant X5	2.139025	1.009013	1.588719
NM_001029875,NM_001271890,NM_001271891,NR_073511,XM_005248502,XR_948251	RGS7BP	regulator of G protein signaling 7 binding protein	-1.061849	2.228147	1.578886
NM_006089,NR_033717,XM_006724458,XM_017029219,XM_017029220,XM_017029221,XM_017029222	SCML2	Scm polycomb group protein like 2	1.715550	2.003207	1.562795
XR_947362	LOC105378730	uncharacterized LOC105378730	-1.346672	2.633030	1.562437

NM_001432	EREG	epiregulin	2.147392	-1.144066	1.552058
XR_002956521,XR_002956522,XR_002956523,XR_002956524,XR_002956525	LOC112267988	uncharacterized LOC112267988, transcript variant X5	2.002752	1.362544	1.546250
NM_001168364,NM_001321325,NM_173853	KRTCAP3	keratinocyte associated protein 3	1.022856	2.514825	1.509479
XR_001740993,XR_001740994	LOC105374187	uncharacterized LOC105374187, transcript variant X1	1.025311	2.162398	1.465708
NM_001098672	HEPHL1	hephaestin like 1	2.361183	1.099530	1.450954
NM_001206997,NM_152770,XM_011531816,XM_017007972,XM_017007973,XM_017007974,XM_017007975,XM_017007976,XM_017007977,XM_017007978	CFAP299	cilia and flagella associated protein 299	2.064439	1.427401	1.429468
NR_149032	LOC100507516	uncharacterized LOC100507516	-1.087215	2.182743	1.423279
NM_003102,XR_427488	SOD3	superoxide dismutase 3	2.136661	-1.113855	1.421211
NM_001128602,NM_001306086,NM_005739,XM_005254114,XM_011521151,XM_017021860	RASGRP1	RAS guanyl releasing protein 1	1.362913	2.045408	1.405154
NM_002562,NR_033948,NR_033949,NR_033950,NR_033951,NR_033952,NR_033953,NR_033954,NR_033955,NR_033956,XM_011538419,XM_011538420	P2RX7	purinergic receptor P2X 7	-1.514194	2.070105	1.401252

,XM_017019364,XM_017019365,XM_017019366,XM_017019367					
NM_199243	GPR150	G protein-coupled receptor 150	1.645452	2.878524	1.395954
NM_001142958,NM_152676,XM_011525856,XM_011525858,XM_011525859,XM_011525860,XM_017025603,XM_017025604,XM_024451099	FBXO15	F-box protein 15	1.153383	2.148531	1.395368
NR_027338	TPI1P3	triosephosphate isomerase 1 pseudogene 3	2.167450	2.027024	1.391676
NR_103445	RBFADN	RBFA downstream neighbor	2.685903	1.851403	1.364752
NR_024586	KMT2E-AS1	KMT2E antisense RNA 1	2.403356	1.931231	1.317087
NM_000963	PTGS2	prostaglandin-endoperoxide synthase 2	2.284580	1.050689	1.257866
NR_002833	DPY19L2P1	DPY19L2 pseudogene 1	1.138028	2.387312	1.252699
XR_924254	LOC105373997	uncharacterized LOC105373997	2.483013	1.508124	1.252163
NM_001278736,NM_002985	CCL5	C-C motif chemokine ligand 5	-3.541327	1.323995	1.243273
NR_046273	LOC729683	uncharacterized LOC729683	-2.979534	-1.050821	1.221567
NR_027263	ARHGAP5-AS1	ARHGAP5 antisense RNA 1 (head to head)	-1.667421	-2.246862	1.173762
NR_002724	MBL1P	mannose binding lectin 1, pseudogene	-1.431215	-2.010930	1.139997

NM_001025081,NM_001025090,NM_001025092,NM_001025100,NM_001025101,NM_002385,XM_017025778,XM_017025780,XM_024451185,XM_024451186,XM_024451187,XM_024451188,XM_024451189,XR_001753201,XR_001753202	MBP	myelin basic protein	-2.082732	-1.220957	1.130803
XR_001747305,XR_946233	LOC105378449	uncharacterized LOC105378449, transcript variant X1	2.143218	1.129512	1.106488
NR_146151	RNA45SN3	RNA, 45S pre-ribosomal N3	4.165842	1.163893	1.046637
XR_001740731,XR_001740732,XR_001740733,XR_001740734,XR_940908,XR_940909,XR_940910,XR_940911,XR_940912,XR_940913	LOC105377123	uncharacterized LOC105377123, transcript variant X2	-1.264749	2.464690	1.022877
NR_038985	LINC00856	long intergenic non-protein coding RNA 856	-2.824366	1.739878	1.005368
XR_001753834	LOC107985303	uncharacterized LOC107985303	-1.071684	-2.048686	-1.053320
NR_104131,NR_160779,NR_160780,NR_160781,NR_160782,NR_160783,NR_160784	SLC44A3-AS1	SLC44A3 antisense RNA 1	-2.156772	-1.064179	-1.056557
NM_001190810	AGAP9	ArfGAP with GTPase domain, ankyrin repeat and PH domain 9	-1.929870	-2.561498	-1.070727
NM_001243746,NM_017565,NR_027751,XM_006721959,XM_011524918,XM_017024781	FAM20A	FAM20A golgi associated secretory pathway pseudokinase	-1.528837	-2.004408	-1.098717

,XR_001752543,XR_001752544,XR_002958041,XR_429905,XR_934487					
NR_037633,NR_037634,NR_037635,NR_037636,NR_037637	GJA9-MYCBP	GJA9-MYCBP readthrough	1.583151	-6.563743	-1.105208
XR_001747657,XR_001747658,XR_001747659,XR_001747660	LOC105378561	uncharacterized LOC105378561, transcript variant X2	-1.587816	-2.478238	-1.144179
NR_135646	LOC105371795	uncharacterized LOC105371795	-1.427275	-2.006006	-1.191979
NM_000076,NM_001122630,NM_001122631,NM_001362474,NM_001362475	CDKN1C	cyclin dependent kinase inhibitor 1C	-2.117311	-1.054310	-1.208429
NM_175055	H2BU1	H2B.U histone 1	1.142388	-4.050494	-1.211166
NM_001492	GDF1	growth differentiation factor 1	-1.468405	-2.054005	-1.216842
NM_018897,XM_011511488,XM_011511489,XM_011511490,XM_011511491,XM_011511492,XM_011511493,XM_011511494,XM_011511495,XM_011511497,XM_017004504,XR_922968	DNAH7	dynein axonemal heavy chain 7	-2.272739	-1.156297	-1.234408
NM_018397,XM_006713250,XM_006713251,XM_006713252,XM_011533938,XM_011533939,XM_017006797,XM_017006798,XM_017006799,XR_001740199,XR_002959545	CHDH	choline dehydrogenase	-2.215594	-1.263565	-1.292370

NM_022147	RTP4	receptor transporter protein 4	-2.222652	1.143660	-1.304971
NM_001330450,NM_002758,XM_005257516,XM_006721975,XM_011525026,XM_011525027	MAP2K6	mitogen-activated protein kinase kinase 6	-3.330901	1.493853	-1.340539
NM_001284351,NM_004174	SLC9A3	solute carrier family 9 member A3	-1.814898	-3.595031	-1.342541
NM_003732	EIF4EBP3	eukaryotic translation initiation factor 4E binding protein 3	-2.840868	-1.458297	-1.343346
NM_001199893,NM_001615	ACTG2	actin gamma 2, smooth muscle	-2.321007	-1.100052	-1.359264
NM_001009571,NM_001167940,NM_001363389,NM_001363390,NM_001363391,NM_001363392,NM_001363393,NM_001363394,NM_001363395,NM_001363396,NM_001363397,NM_001363398,NM_001363399,NM_001363400,NM_017954,XM_005250696,XM_005250697,XM_005250699,XM_005250701,XM_005250702,XM_005250704,XM_005250706,XM_005250707,XM_017012794,XM_017012796,XM_017012798,XM_024446998	CADPS2	calcium dependent secretion activator 2	-2.195323	-1.011096	-1.364656
NM_000866,NM_001322208,NM_001322209,NM_001322210,XM_	HTR1F	5-hydroxytryptamine receptor 1F	-1.583667	2.521264	-1.365979

005264751,XM_011533664					
XR_936221	LOC105372232	uncharacterized LOC105372232	-1.520445	-2.635292	-1.386243
NR_145424,NR_145425,NR_145426	RUSC1-AS1	RUSC1 antisense RNA 1	-1.311818	-2.562030	-1.395740
NM_000934,NM_001165920,NM_001165921,XM_005256701,XM_017024765	SERPINF2	serpin family F member 2	-2.508668	-2.136700	-1.416537
NM_001318835,NM_005794,NM_182908,XM_005267249,XM_006720001,XM_011536338,XM_011536339,XR_001750105,XR_001750106,XR_001750107,XR_943366,XR_943367	DHRS2	dehydrogenase/reductase 2	-2.107583	-1.595126	-1.423498
NM_001113410,NM_002785,NM_203287	PSG11	pregnancy specific beta-1-glycoprotein 11	-1.902515	-2.851903	-1.446695
NM_001035516,NM_001126056,NM_001126057,NM_001126058,NM_001126059,NM_001190347,NM_001190348,NM_001190349,NM_001308380,NM_001308383,NM_001352328,NM_001352329,NM_001352330,NM_001352331,NM_001352332,NM_033317,NR_033746,NR_147958,NR_147959,XM_006723477,XM_006723484,XM_006723489,XM_006723493,XM_006723494	DMKN	dermokine	-2.034995	-1.098501	-1.455930

,XM_006723503,XM_011527494,XM_011527495,XM_011527496,XM_011527497,XM_011527498,XM_011527499,XM_011527500,XM_011527501,XM_011527502,XM_011527503,XM_011527504,XM_011527505,XM_011527506,XM_011527507,XM_011527508,XM_011527509,XM_011527513,XM_017027475,XM_024451775,XM_024451776,XM_024451777,XM_024451778,XM_024451779,XR_002958383,XR_002958384,XR_935871,XR_935872,XR_935873,XR_935874					
NM_001146256,NM_001146257,NM_144969,XM_006724624,XM_017029296,XM_017029297,XM_017029298,XM_024452342,XR_001755658,XR_002958764,XR_938393	ZDHHC15	zinc finger DHHC-type palmitoyltransferase 15	-2.082889	-1.245901	-1.465539
NM_018904,NM_031865	PCDHA13	protocadherin alpha 13	-2.357481	-2.859852	-1.472952
NM_001199219,NM_006774	INMT	indolethylamine N-methyltransferase	1.337704	-2.173857	-1.484727
NM_001145033	C11orf96	chromosome 11 open reading frame 96	-3.007217	-1.066124	-1.486388
XM_011520896	LOC105369669	uncharacterized LOC105369669	-1.369606	-2.165458	-1.495527

NM_018906,NM_031497	PCDHA3	protocadherin alpha 3	-2.296612	1.390568	-1.530374
NM_001080433,NM_001348512,NM_001348513,NM_001348514,NM_001348515,NM_001348516,XM_024452642	CCDC85A	coiled-coil domain containing 85A	-2.142808	-1.308840	-1.532671
NM_000063,NM_001145903,NM_001178063,NM_001282457,NM_001282458,NM_001282459	C2	complement C2	-2.254828	-1.328373	-1.534662
NM_001099,NM_001134194,NM_001292037,XM_011512946,XM_011512947	ACP3	acid phosphatase 3	1.985690	-2.881223	-1.537117
NR_149036	LOC100128653	uncharacterized LOC100128653	-3.012878	-1.575937	-1.537786
NM_003510	H2AC15	H2A clustered histone 15	-1.752607	-2.797482	-1.553849
NM_001320727,NR_135324	SETDB2-PHF11	SETDB2-PHF11 readthrough	-2.646332	-1.085604	-1.555124
NM_001318144,NM_004925,XR_001746289	AQP3	aquaporin 3 (Gill blood group)	-2.712616	-2.325015	-1.570621
NM_005472,XM_011544713,XM_017017047,XM_017017048,XM_017017049,XM_017017051,XM_017017052	KCNE3	potassium voltage-gated channel subfamily E regulatory subunit 3	-2.155556	-1.662134	-1.602008
NM_014586,XM_011529537	HUNK	hormonally up-regulated Neu-associated kinase	-2.019552	-1.146296	-1.610701
NM_001242935,NM_001308100,NM_001308105,NM_001308106,NM_001308107,NM_00	SNCAIP	synuclein alpha interacting protein	-2.092519	-1.179303	-1.614890

1308108,NM_001308109,NM_005460,NR_131761,NR_131762,XM_005272138,XM_005272139,XM_006714734,XM_011543737,XM_011543738,XM_011543739,XM_011543741,XM_011543743,XM_011543745,XM_011543749,XM_011543750,XM_017010078,XM_017010079,XM_017010080,XM_017010081,XM_017010082,XM_017010083,XM_017010084,XM_017010085,XM_017010086,XM_024446266,XM_024446267,XM_024446268,XM_024446269,XR_001742362,XR_001742363,XR_001742364,XR_001742365,XR_001742366					
NM_033452,XM_005257787,XM_005257788	TRIM47	tripartite motif containing 47	-2.405871	-1.441607	-1.622046
NM_152228,XM_017002435,XM_017002436	TAS1R3	taste 1 receptor member 3	-2.084855	-2.013670	-1.633833
NR_024041	CETN4P	centrin 4, pseudogene	-1.471649	-2.137985	-1.637997
NM_006897	HOXC9	homeobox C9	-2.004325	-1.290556	-1.643935
NR_120685,NR_120686	LINC01503	long intergenic non-protein coding RNA 1503	-2.324592	-1.757226	-1.645597

NM_017831,XM_011526045,XM_011526046,XM_011526047	RNF125	ring finger protein 125	-2.023977	-1.243471	-1.653609
NR_120648	LOC101928994	uncharacterized LOC101928994	-2.009774	-1.534307	-1.658814
NM_000527,NM_001195798,NM_001195799,NM_001195800,NM_001195803,XM_011528010,XR_001753685,XR_001753686	LDLR	low density lipoprotein receptor	-1.145947	-2.020541	-1.675460
NM_000503,NM_001288574,NM_001288575,NM_001370333,NM_001370334,NM_001370335,NM_001370336,NM_172058,NM_172059,XM_011517483,XM_011517484,XM_017013202,XM_017013203,XM_017013204,XM_017013205,XM_017013207,XM_017013208,XM_017013211,XM_017013212,XM_017013213	EYA1	EYA transcriptional coactivator and phosphatase 1	-2.409474	-1.422681	-1.693060
NM_001100812,NM_022059	CXCL16	C-X-C motif chemokine ligand 16	-2.107602	-1.216888	-1.710767
NM_000337,NM_001128209,NM_172244,XM_005265966,XM_005265967,XM_011534621,XM_017009723,XM_017009724	SGCD	sarcoglycan delta	-2.100540	1.163717	-1.717182
NM_014391	ANKRD1	ankyrin repeat domain 1	-2.461539	-1.193961	-1.721281
NR_103777	LRIG2-DT	LRIG2 divergent transcript	-1.158773	-2.289531	-1.728223

gene-KRT8P33	KRT8P33	keratin 8 pseudogene 33	-2.416949	-1.402228	-1.728825
NM_001031804,NM_005360,XM_017023233,XM_017023234,XM_017023235,XM_024450279,XR_001751902,XR_002957802,XR_002957803,XR_002957804	MAF	MAF bZIP transcription factor	-2.071680	-1.074606	-1.733804
NM_003394,XM_011538722,XM_011538724,XM_017019919,XM_024449179	WNT10B	Wnt family member 10B	-2.324930	-2.131322	-1.735937
NM_001365276,NM_019105,NM_032470	TNXB	tenascin XB	-1.238641	-2.026914	-1.741096
NM_001079691,NM_001286459,NM_001286460,NM_001286461,NM_001353627,NM_001353628,NM_001353629,NM_001353630,NM_001353631,NM_001353632,NM_001353633,NM_001353634,NM_001353635,NM_001353636,NM_001353637,NM_052818,NR_148475,NR_148476,NR_148477,NR_148478,NR_148479,NR_148480,XM_011535303,XM_017020838,XM_017020840,XM_017020841,XM_024449431	N4BP2L1	NEDD4 binding protein 2 like 1	-2.100621	-1.565818	-1.745948
gene-PCNPP1	PCNPP1	PEST containing nuclear protein pseudogene 1	-2.321272	-2.234230	-1.747104

NM_001001522,NM_003186	TAGLN	transgelin	-2.246697	-1.023750	-1.752129
NR_027244	LINC01106	long intergenic non-protein coding RNA 1106	-2.245199	-1.787402	-1.760732
NM_002430	MN1	MN1 proto-oncogene, transcriptional regulator	-2.401204	-1.373995	-1.761428
NM_020433,NM_175913,XM_006723833	JPH2	junctophilin 2	-2.008315	-1.184092	-1.764787
NM_001144763,NM_001144764,NM_001144765,NM_001308019,NM_015065,XM_011542696,XM_011542698,XM_011542700,XM_017017397,XM_017017398,XM_017017399,XM_017017400,XM_017017401,XM_017017402,XM_017017403,XM_017017404	EXPH5	exophilin 5	-2.219982	-1.142540	-1.782871
NM_002848,NM_030667,NM_030668,NM_030669,NM_030670,NM_030671,XM_017019725,XR_931316,XR_931318	PTPRO	protein tyrosine phosphatase receptor type O	-2.463280	1.064026	-1.783940
NM_012275,NM_173170	IL36RN	interleukin 36 receptor antagonist	-2.012724	-1.699543	-1.785961
NM_003806,NR_073189,XM_017020146	HRK	harakiri, BCL2 interacting protein	-2.778222	-1.233590	-1.799306
NM_021255,XM_005267890,XM_006720211,XM_011536990,XM_011536992,XM_017021478,XM_017021479	PELI2	pellino E3 ubiquitin protein ligase family member 2	-2.079137	-1.249793	-1.805147

XR_242166	SUGCT-AS1	SUGCT antisense RNA 1	-3.014671	-1.644335	-1.810304
NM_000880,NM_001199886,NM_001199887,NM_001199888,XM_011517522,XM_011517523,XM_017013397	IL7	interleukin 7	-2.940321	-1.107627	-1.820700
NM_001135556,NM_001135557,NM_001278421,NM_001278422,NM_004411	DYNC1I1	dynein cytoplasmic 1 intermediate chain 1	-2.747300	-1.513404	-1.822992
NM_001206802,NM_004881,NM_147184,XM_005264650,XM_006712150,XM_024453249	TP53I3	tumor protein p53 inducible protein 3	-2.075309	-1.529997	-1.834272
XR_001741738,XR_001741739,XR_001741740	LOC105377294	uncharacterized LOC105377294, transcript variant X1	-2.042387	-1.701813	-1.837127
XR_947744	LOC105378929	uncharacterized LOC105378929	-2.918207	-1.920672	-1.851605
NM_171998	RAB39B	RAB39B, member RAS oncogene family	-2.481988	-1.328132	-1.875539
NM_020311,XM_005246097,XM_005246098,XM_017004516	ACKR3	atypical chemokine receptor 3	-2.009274	-1.078982	-1.875969
NM_001271856,NM_181711,XM_005268691,XM_011537996	TAMALIN	trafficking regulator and scaffold protein tamalin	-2.091798	-1.841329	-1.877918
NM_001142883,NM_054111,XM_005248842,XM_005248843,XM_011514295,XM_024446323,XM_024446324,XM_024446325,XM_024446326,XM_024446327	IP6K3	inositol hexakisphosphate kinase 3	-2.707459	-1.071217	-1.889766

NM_001271946,NM_003062,XM_017009779	SLIT3	slit guidance ligand 3	-2.264584	-1.227388	-1.896080
NM_001171940,NM_001171941,NM_153756	FNDC5	fibronectin type III domain containing 5	-2.008142	1.016567	-1.917984
gene-LOC100420326	LOC100420326	ferritin heavy chain like 17 pseudogene	-3.219411	-3.366291	-1.918617
NM_001128588,NM_001146036,NM_001146037,NM_001308278,NM_001308279,NM_015865,XM_005258329,XM_005258333,XM_006722526,XM_011526142,XM_011526144,XM_024451238,XR_001753266	SLC14A1	solute carrier family 14 member 1 (Kidd blood group)	-1.046991	-2.160738	-1.923742
XR_946246	LOC105378452	uncharacterized LOC105378452	-1.357651	-2.348427	-1.930594
XR_949348	LOC105373264	uncharacterized LOC105373264	-2.159536	-1.836514	-1.942937
gene-GPAA1P1	GPAA1P1	glycosylphosphatidylinositol anchor attachment 1 pseudogene 1	-2.411505	-1.579056	-1.944014
NM_004202	TMSB4Y	thymosin beta 4 Y-linked	-2.705241	-1.000397	-1.956736
NM_005545,NM_201526	ISLR	immunoglobulin superfamily containing leucine rich repeat	-2.789021	-1.439731	-1.959681
NM_032532,XM_011536190,XM_011536191	FNDC1	fibronectin type III domain containing 1	-2.412644	-1.339565	-1.974444
NM_001080480,NR_073465,XM_006714999,XM_006715000,XM_011514313	MBOAT1	membrane bound O-acyltransferase domain containing 1	-2.626969	-1.094343	-1.997176

NM_203371	FIBIN	fin bud initiation factor homolog	-2.044229	-1.311792	-1.997336
NM_014867,XM_011534771,XM_011534772,XM_017014114,XM_017014115,XM_017014116,XM_017014117	KBTBD11	kelch repeat and BTB domain containing 11	-2.730207	-1.114679	-2.000272
NR_144550	SDAD1P1	SDA1 domain containing 1 pseudogene 1	-1.340346	-1.192062	-2.002095
NM_016224,XM_005267015,XM_011535886	SNX9	sorting nexin 9	-1.515377	-1.623809	-2.003129
NM_003966,XM_006714506,XM_006714507,XM_011514155,XM_011514156,XM_011514157,XM_011514158,XM_011514159,XM_017010016	SEMA5A	semaphorin 5A	-1.333167	-1.280721	-2.006606
XR_001742582,XR_001742583,XR_001742584,XR_001742585,XR_001742586,XR_001742587,XR_002956209,XR_241729,XR_925722,XR_925725	LOC101929200	uncharacterized LOC101929200, transcript variant X3	-1.847629	-1.681927	-2.007709
NM_001168399,NM_001168400,NM_001168401,NM_032621	BEX2	brain expressed X-linked 2	-1.804873	-1.442432	-2.008317
NM_144717,XM_006713665,XM_011512910,XM_011512911,XM_017006632	IL20RB	interleukin 20 receptor subunit beta	-1.522526	-1.072850	-2.008548
NM_001290360,NM_001324101,NM_001324103,NM_001324106,NM_001324107,NM_001324108,NM_001324109,NM_001324111,N	EBF1	EBF transcription factor 1	-1.941968	-1.374246	-2.008627

M_001364155,NM_001364156,NM_001364157,NM_001364158,NM_001364159,NM_024007,NM_182708,XM_017009192,XM_017009193,XM_017009194,XM_017009195,XM_017009196,XM_017009197,XM_017009199,XM_017009200,XM_017009201,XM_017009202,XM_017009203,XM_017009204,XM_024454390,XM_024454391,XM_024454392,XM_024454393					
NM_006690,XM_011528500,XM_017027597,XM_017027598	MMP24	matrix metallopeptidase 24	-1.601770	-1.583015	-2.008955
NM_001282466,NM_001282467,NM_021197,XM_024450364,XM_024450365	WFDC1	WAP four-disulfide core domain 1	-2.314650	-1.048485	-2.012386
NM_022160	DMRTA1	DMRT like family A1	-2.155269	-1.679983	-2.015001
NM_001349413,NM_001349414,NM_001349415,NM_001349416,NM_001349417,NM_001349418,NM_001349419,NM_001349420,NM_001349421,NM_001349422,NM_001349423,NM_001349424,NM_001349425,NM_001349426,NM_001349428,NM_001349429,NM_017644,NR_146169,NR_146170,XM_0052	KLHL24	kelch like family member 24	-1.708477	-1.239186	-2.015590

47552,XM_017006653,XM_017006655,XM_017006658,XM_017006661,XM_024453607					
NM_014618	BRINP1	BMP/retinoic acid inducible neural specific 1	-2.032743	-1.529080	-2.016566
NM_001363598,NM_031412	GABARAPL1	GABA type A receptor associated protein like 1	-1.438015	-1.385563	-2.017838
NR_047482	DOCK9-DT	DOCK9 divergent transcript	-1.859472	-2.093548	-2.017976
NR_110997	MIR3936HG	MIR3936 host gene	-1.661312	-1.890144	-2.018128
NR_027145	LIMS3-LOC440895	LIMS3-LOC440895 readthrough	-2.367871	-1.492554	-2.019191
NM_001205254,NM_001205255,NM_002538,XM_017008913,XM_017008914	OCLN	occludin	-1.855087	-1.387537	-2.022327
NM_005941,XM_024447154	MMP16	matrix metallopeptidase 16	-1.490404	-1.352030	-2.023141
NM_001098272,NM_001324219,NM_001324220,NM_001324222,NM_001324223,NM_001324224,NM_001330663,NM_001364188,NM_002130,XM_011514036,XM_024446038	HMGCS1	3-hydroxy-3-methylglutaryl-CoA synthase 1	-1.630635	-1.631366	-2.023242
NM_005144,NM_018411,XM_005273569,XM_006716367	HR	HR lysine demethylase and nuclear receptor corepressor	-1.712156	-1.386044	-2.024576
NM_001018109,NM_003662	PIR	pirin	-2.352349	-1.324286	-2.024778
NM_002775	HTRA1	HtrA serine peptidase 1	-1.774919	-1.326376	-2.025064

XR_001747518	LOC107984248	uncharacterized LOC107984248	-1.623551	-1.645705	-2.026363
XR_001741421	LOC105379404	uncharacterized LOC105379404	1.145350	-1.099557	-2.030310
NM_003013	SFRP2	secreted frizzled related protein 2	-1.404158	-1.636545	-2.030420
NM_001039538,NM_001363910,NM_001363911,NM_001363913,NM_001375474,NM_001375493,NM_001375494,NM_001375495,NM_001375496,NM_001375497,NM_001375498,NM_001375499,NM_001375500,NM_001375501,NM_001375502,NM_001375503,NM_001375504,NM_001375505,NM_001375506,NM_001375507,NM_001375508,NM_001375509,NM_001375510,NM_001375526,NM_001375527,NM_001375528,NM_001375529,NM_001375530,NM_001375531,NM_001375532,NM_001375533,NM_001375534,NM_001375535,NM_001375536,NM_001375537,NM_001375538,NM_001375539,NM_001375540,NM_001375541,NM_001375542,NM_001375543,NM_001375544,NM_001375545,NM_001375546,N	MAP2	microtubule associated protein 2	-1.614344	-1.472735	-2.031583

M_001375548,NM_001375551,NM_001375552,NM_001375553,NM_001375554,NM_001375555,NM_001375556,NM_001375557,NM_001375558,NM_001375559,NM_001375583,NM_002374,NM_031845,NM_031847,NR_164694,NR_164695,NR_164696,NR_164697,NR_164698,NR_164699,XM_005246565,XM_005246566,XM_011511195,XM_011511196,XM_011511197,XM_017004112,XM_017004113,XM_017004114,XM_017004116,XM_017004122,XM_017004128,XM_017004129,XM_017004130,XM_017004131,XM_017004138,XM_024452893,XM_024452894,XM_024452895,XM_024452896,XM_024452897,XM_024452899,XM_024452902,XM_024452906,XM_024452907					
NM_020431	TMEM63C	transmembrane protein 63C	-1.233495	-1.355250	-2.032061
NM_019074	DLL4	delta like canonical Notch ligand 4	1.389587	-1.838511	-2.033686
NM_033199	UCN2	urocortin 2	-1.462787	-1.363332	-2.038341

NM_018398	CACNA2D3	calcium voltage-gated channel auxiliary subunit alpha2delta 3	-1.423364	-1.340109	-2.040024
NM_002148	HOXD10	homeobox D10	-1.852255	-1.050196	-2.040354
XR_001737764	LOC105373265	uncharacterized LOC105373265, transcript variant X1	-2.407475	-1.497868	-2.044734
NM_001099743,NM_001099744,NM_001099745,NM_001099746,NM_001099747,NM_001099748,NM_001099749,NM_001099750,NM_001099751,NM_001099752,NM_001099753,NM_001099754,NM_001099755,NM_001099756,NM_001330596,NM_001363032,NM_017786,XM_011517154,XM_017013614,XM_017013615	SYBU	syntabulin	-1.942654	-1.478782	-2.045594
NM_001367607,NR_160272,XM_011525664,XM_011525665,XM_011525666,XM_024451167,XM_024451168,XR_001753189,XR_001753190,XR_001753191,XR_935057,XR_935058,XR_935059,XR_935060,XR_935061,XR_935062	ANKRD30B	ankyrin repeat domain 30B	-1.744805	-1.208652	-2.045604
XR_001755024,XR_001755025,XR_001755026,XR_937695	LOC105369301	uncharacterized LOC105369301, transcript variant X4	-2.338665	1.164866	-2.045745
NM_015419	MXRA5	matrix remodeling associated 5	-1.755692	1.115923	-2.046256

NM_001142502,NM_006663,XM_017026177,XM_017026178,XM_017026179	PPP1R13L	protein phosphatase 1 regulatory subunit 13 like	-1.764906	-1.473848	-2.046941
XR_001742939	LOC107986465	uncharacterized LOC107986465	-1.868818	-1.462153	-2.053824
NM_001174060,NM_017784,XM_005264843,XM_005264844,XM_005264845,XM_011533326,XM_017005670	OSBPL10	oxysterol binding protein like 10	-1.683044	-1.264438	-2.055407
NM_002345	LUM	lumican	-1.364910	-1.571469	-2.055410
NM_001322286,NM_001322290,NM_001322291,NM_145756,XM_006722432,XM_017025687,XM_017025688,XM_017025689,XM_024451138	ZNF396	zinc finger protein 396	-1.623227	-1.075461	-2.056831
NR_002924	TBC1D3P1-DHX40P1	TBC1D3P1-DHX40P1 readthrough, transcribed pseudogene	-2.062674	-1.496103	-2.057306
NM_001034173,NR_027752,XM_011537986,XM_011537988,XM_011537989,XM_017018889,XM_017018890	ALDH1L2	aldehyde dehydrogenase 1 family member L2	-1.449050	-1.134608	-2.058476
NM_001025580,NM_001171689,NM_015365	AMMECR1	AMMECR nuclear protein 1	-1.610162	-1.314478	-2.058828
NM_001287437,NM_001287439,NM_001287440,NM_001287441,NM_001287442,NM_001287443,NM_024900,NM_199320,XM_005263232,XM_017008626,XM_017008627,XM_	JADE1	jade family PHD finger 1	-1.743486	-1.188235	-2.060403

024454217,XM_024454218,XM_024454219,XM_024454220,XM_024454221					
gene-RNU6-3P	RNU6-3P	RNA, U6 small nuclear 3, pseudogene	-1.561714	-1.940925	-2.060586
NM_001288748,NM_182904,NR_110031,XR_001747836,XR_001747837,XR_001747838,XR_001747839,XR_001747840	P4HA3	prolyl 4-hydroxylase subunit alpha 3	-1.845669	-1.321188	-2.060774
NM_013281,NM_198391,XM_005260682,XM_011529204,XM_011529205	FLRT3	fibronectin leucine rich transmembrane protein 3	-1.557507	-1.403812	-2.061344
NM_001146254,NM_001146255,NM_001370095,NM_024825,XM_006722902,XM_006722903,XM_011528308,XM_011528309,XM_011528310,XM_011528311,XM_011528313,XM_011528314,XM_011528316,XM_017027313,XM_024451726	PODNL1	podocan like 1	-1.940234	-1.451267	-2.062501
NM_001033553,NM_001033554,NM_001033555,NM_001243438,NM_001243439,NM_152904,XM_005256860,XM_011524075,XM_017025317,XM_017025318,XM_017025319,XM_017025320,XM_017025321,XM_017025322,XM_017025323,XM_017025324,XM_01	SPECC1	sperm antigen with calponin homology and coiled-coil domains 1	-1.761302	-1.486116	-2.069383

7025325,XR_001752682					
NM_001282715,NM_182487,XM_005251760,XM_006716989	OLFML2A	olfactomedin like 2A	-2.987550	-1.073971	-2.070454
NM_001320437,NM_001320441,NM_001350995,NM_001378208,NM_001378209,NM_001378210,NM_152586,NR_135249,NR_135250,NR_146997,NR_146998,NR_165442,NR_165443,XM_005269582,XM_011539368,XM_017015774,XM_017015775,XM_017015777,XM_017015782,XM_017015783,XM_024447832,XM_024447833,XM_024447834,XM_024447835,XM_024447836,XM_024447837,XM_024447838,XM_024447839,XM_024447840,XM_024447841,XM_024447842,XM_024447843,XM_024447844,XM_024447845,XM_024447846,XM_024447847,XM_024447848,XM_024447849,XM_024447850,XM_024447851,XM_024447852,XR_001747036,XR_001747037,XR_001747038,XR_001747039,XR_001747041,XR_001747042,XR_001747043,XR_001747044,XR	USP54	ubiquitin specific peptidase 54	-1.465655	-1.515867	-2.073680

_001747047,XR_001747048,XR_002956959,XR_002956960,XR_002956961,XR_002956962					
NR_138037	HCG20	HLA complex group 20	-1.381680	-1.623396	-2.074976
NM_174911,NR_156466,XM_017013107,XM_017013108	LRATD2	LRAT domain containing 2	-1.948972	-1.361120	-2.076281
NM_001286262,NM_152772,XM_005268767,XM_005268768,XM_011538129,XM_011538130,XM_011538131,XM_011538132,XM_017019127,XM_017019129,XM_017019130,XM_017019131,XM_017019132,XM_017019133	TCP11L2	t-complex 11 like 2	-1.528782	-1.416069	-2.080238
NM_001018053,NM_006212,XM_005273162,XM_024447654,XM_024447655,XM_024447656,XM_024447657	PFKFB2	6-phosphofructo-2-kinase/fructose-2,6-biphosphatase 2	-1.926178	-1.490516	-2.082130
NM_001114309,NM_004433,XM_005244942	ELF3	E74 like ETS transcription factor 3	-1.866891	-1.081950	-2.082778
NM_001142314,NM_001321519,NM_080876,NR_135688	DUSP19	dual specificity phosphatase 19	-1.626324	-1.184798	-2.083806
NM_001004019,NM_001165035,NM_001998,XM_006713026	FBLN2	fibulin 2	-1.936595	-1.513342	-2.085647
NM_001193482,NM_001193483,NM_001193484,NM_001193485,NM_001193488,NM_001371494,NM_0013714	LIMS1	LIM zinc finger domain containing 1	-1.660080	-1.339670	-2.086167

95,NM_001371496,NM_001371497,NM_001371498,NM_001371499,NM_001371500,NM_004987					
XR_929077	LOC105375913	uncharacterized LOC105375913	-1.764717	1.334861	-2.087410
NM_001142699,NM_001142700,NM_001142702,NM_001206769,NM_001300983,NM_001351274,NM_001351275,NM_001351276,NM_001364,NM_001377966,NM_001377967,NM_001377968,NM_001377970,NM_001377971,NM_001377972,NM_001377973,NM_001377974,NM_001377975,NM_001377976,NM_001377977,NM_001377978,NM_001377979,NM_001377980,NM_001377981,NM_001377982,NM_001377983,NR_165353,XM_005273810,XM_011544778,XM_011544780,XM_011544782,XM_017017254,XM_017017255,XM_017017256,XM_017017257,XM_017017258,XM_017017261,XM_017017262,XM_017017263,XM_017017264,XM_017017265,XM_017017267,XM_017017268,XM_017017269,XM_0170172	DLG2	discs large MAGUK scaffold protein 2	-1.412871	-1.796775	-2.087764

70,XM_017017271,XM_017017273,XM_017017276,XM_017017277,XM_017017279,XM_017017280,XM_017017281,XM_017017284,XM_017017285,XM_017017286,XM_017017287,XM_017017288,XM_017017289,XM_017017290,XM_024448378,XM_024448379					
NM_000029,NM_001382817	AGT	angiotensinogen	-3.305283	-1.571602	-2.087893
NM_000393,XM_011510573	COL5A2	collagen type V alpha 2 chain	-1.364103	-1.366422	-2.088943
NR_024569	LOC100130872	uncharacterized LOC100130872	-1.668286	-1.376893	-2.089535
NM_001123066,NM_001123067,NM_001203251,NM_001203252,NM_001377265,NM_001377266,NM_001377267,NM_001377268,NM_005910,NM_016834,NM_016835,NM_016841,NR_165166,XM_005257362,XM_005257365,XM_005257366,XM_005257367,XM_005257368,XM_005257369,XM_005257370,XM_005257371	MAPT	microtubule associated protein tau	-1.765694	-1.775392	-2.101268
NM_000428,XM_011536765	LTBP2	latent transforming growth factor beta binding protein 2	-2.063272	-1.073429	-2.102585

NM_000433,NM_001127651,NM_001190789,NM_001190794,XM_005245207,XM_011509580,XM_011509581	NCF2	neutrophil cytosolic factor 2	-2.049864	-1.257323	-2.105720
NM_001008844,NM_001319034,NM_004415	DSP	desmoplakin	-1.869138	-1.249048	-2.106107
NM_001318918,NM_032575	GLIS2	GLIS family zinc finger 2	-1.680821	-1.563392	-2.107611
NM_001201427,NM_015345,XM_006715039,XM_006715040,XM_006715042,XM_006715043,XM_006715045,XM_006715046,XM_017010630	DAAM2	dishevelled associated activator of morphogenesis 2	-1.929609	-1.438353	-2.110467
NM_001884,XM_011543168,XM_017009051,XM_017009052,XM_017009053,XM_017009054	HAPLN1	hyaluronan and proteoglycan link protein 1	-1.805561	-1.717362	-2.111473
NM_000376,NM_001017535,NM_001017536,NM_001364085,NM_001374661,NM_001374662,XM_011538720,XM_024449178	VDR	vitamin D receptor	-1.634044	-1.434861	-2.112666
NM_001289	CLIC2	chloride intracellular channel 2	-2.116524	-1.689961	-2.113504
NM_024080,XM_011511810,XM_017004891,XM_024453132,XM_024453133,XM_024453134	TRPM8	transient receptor potential cation channel subfamily M member 8	-1.621518	-1.787103	-2.115707
NM_001010923,NM_001164685,NM_001164687,NM_001318531,XM_011535814,XM_01	THEMIS	thymocyte selection associated	-1.881853	-1.378448	-2.117576

1535816,XM_017010848,XM_017010849,XM_024446433,XM_024446434					
NR_160425,NR_160426,NR_160427	BMS1P4-AGAP5	BMS1P4-AGAP5 readthough	1.043753	1.021792	-2.125681
NM_001256324,NM_001256325,NM_001256326,NM_001256327,NM_001256328,NM_001256329,NM_001256330,NM_001256331,NM_001256332,NM_001256333,NM_001256334,NM_001256359,NM_001256360,NM_001256361,NM_018896,NM_198376,NM_198377,NM_198378,NM_198379,NM_198380,NM_198382,NM_198383,NM_198384,NM_198385,NM_198386,NM_198387,NM_198388,NM_198396,NR_046054,NR_046055,NR_046056,NR_046057,NR_046058,XM_006722160,XM_006722161	CACNA1G	calcium voltage-gated channel subunit alpha1 G	-1.770830	-1.722278	-2.128080
XR_923227	LOC105373570	uncharacterized LOC105373570	-2.307093	-1.172900	-2.128650
NM_001329564,NM_032367,NR_138050	ZBED3	zinc finger BED-type containing 3	-1.479983	-1.181207	-2.130176
gene-SLC16A6P1	SLC16A6P1	SLC16A6 pseudogene 1	-1.115774	-2.629117	-2.133794
NM_001136494,NM_001136495,NM_032800,XM_017002599	C1orf198	chromosome 1 open reading frame 198	-1.755607	-1.387614	-2.134383

NM_001355016,NM_001355017,NM_002609,NR_149150	PDGFRB	platelet derived growth factor receptor beta	-1.682302	-1.506582	-2.136990
NM_001144950,NM_001195267	SSC5D	scavenger receptor cysteine rich family member with 5 domains	-1.986730	-1.431618	-2.137124
NM_024795,XM_011511876	TM4SF20	transmembrane 4 L six family member 20	-4.724534	-1.148934	-2.139697
NM_014840	NUAK1	NUAK family kinase 1	-1.938969	-1.185646	-2.141097
XR_110231	LOC100506731	uncharacterized LOC100506731	-3.109774	-1.406949	-2.146241
XM_017023965,XM_017023966	LOC107984138	serine/threonine-protein kinase SMG1-like, transcript variant X1	-1.537733	-1.247922	-2.146690
NM_001042483,NM_012385	NUPR1	nuclear protein 1, transcriptional regulator	-1.721467	-1.483634	-2.149119
NM_001256105,NM_001377271,NM_001377272,NM_003392,XM_011534085,XM_011534086,XM_011534088,XM_011534089,XM_017007127,XM_017007128	WNT5A	Wnt family member 5A	-1.382403	-1.354953	-2.152336
NM_001381939,NM_001381940,NM_001381941,NM_001381942,NM_003247,NR_167744,NR_167745	THBS2	thrombospondin 2	-1.810205	-1.246539	-2.152920
NM_001034954,NM_001034955,NM_001034956,NM_001034957,NM_001290294,NM_001290295,NM_0012902	SORBS1	sorbin and SH3 domain containing 1	-1.620603	1.059384	-2.155289

96,NM_001290297,NM_001290298,NM_001377197,NM_001377198,NM_001377199,NM_001377200,NM_001377201,NM_001377202,NM_001377203,NM_001377204,NM_001377205,NM_001377206,NM_001377207,NM_001377208,NM_001377209,NM_006434,NM_015385,NM_024991,XM_006717589,XM_006717593,XM_011539140,XM_011539150,XM_011539155,XM_011539167,XM_017015500,XM_017015501,XM_017015502,XM_017015503,XM_017015504,XM_017015505,XM_017015506,XM_017015507,XM_017015508,XM_017015509,XM_017015510,XM_017015511,XM_017015512,XM_017015513,XM_017015514,XM_017015515,XM_017015517,XM_017015523,XM_017015525,XM_017015530,XM_017015532,XM_017015533,XM_017015536,XM_017015537,XM_017015539,XM_017015540,XM_024447769,XM_024447770

NM_001371116,NM_033393,XM_005263319,XM_011532389	FHDC1	FH2 domain containing 1	-1.645423	-1.752897	-2.156731
XR_002958414	LOC112268244	uncharacterized LOC112268244	-1.308646	-1.575353	-2.158762
NM_001198,NM_182907,XM_006715550,XM_011536062,XM_011536063,XM_011536064,XM_017011187	PRDM1	PR/SET domain 1	-1.334029	-1.498742	-2.159476
NM_001145536	C17orf107	chromosome 17 open reading frame 107	-1.621810	-1.443346	-2.161715
NM_001378183,NM_022068,XM_011525723,XM_011525724,XM_011525725,XM_011525726,XM_017025918,XR_001753259	PIEZO2	piezo type mechanosensitive ion channel component 2	-1.486586	-1.266172	-2.164081
NM_014951,NM_199450,XM_017015937	ZNF365	zinc finger protein 365	-1.881883	-1.428843	-2.165843
NM_173582,XM_011544953	PGM2L1	phosphoglucomutase 2 like 1	-1.613759	-1.407380	-2.169347
NM_001286777,NM_001286778,NM_031461	CRISPLD1	cysteine rich secretory protein LCCL domain containing 1	-3.304789	-1.518056	-2.169557
NM_001330322,NM_001330323,NM_002146,XM_005257277,XM_006721854,XM_011524708,XM_011524710,XM_011524719,XM_011524720,XM_011524721,XM_011524726,XM_017024560,XM_024450737	HOXB3	homeobox B3	-1.301847	-1.410683	-2.173464
XR_001751773,XR_001751776,XR_001751777,XR_002957755,XR	LOC107984805	uncharacterized LOC107984805, transcript variant X3	-1.562111	-1.182962	-2.173941

_002957756,XR_002957757,XR_002957758,XR_002957759,XR_002957760,XR_002957761					
NM_014243,XM_011532421,XM_011532422	ADAMTS3	ADAM metallopeptidase with thrombospondin type 1 motif 3	-2.153459	1.057901	-2.176080
NM_018931	PCDHB11	protocadherin beta 11	-2.475881	-1.652975	-2.177064
NM_001256099,NM_138455,XM_011516824	CTHRC1	collagen triple helix repeat containing 1	-2.123846	-1.015142	-2.177962
NM_181616	KRTAP20-2	keratin associated protein 20-2	-1.467827	-1.165876	-2.178838
NM_002015,XM_011535008,XM_011535010	FOXO1	forkhead box O1	-1.766567	-1.178650	-2.182438
NM_013324,NM_145071,XM_011533329	CISH	cytokine inducible SH2 containing protein	-1.969331	-1.430535	-2.189614
NM_001018100,NM_152451	MYZAP	myocardial zonula adherens protein	-1.094832	-1.848504	-2.190080
NM_001920,NM_133503,NM_133504,NM_133505,NM_133506,NM_133507	DCN	decorin	-1.546678	-1.649881	-2.197143
NM_001406	EFNB3	ephrin B3	-1.615508	-1.281022	-2.198336
NR_047507	HOXC13-AS	HOXC13 antisense RNA	-2.618281	-1.683209	-2.204972
NM_001198595,NM_006873	STON1	stonin 1	-1.698572	-1.547720	-2.205280
NM_005559	LAMA1	laminin subunit alpha 1	-1.894169	-1.270169	-2.210911

XR_002958137,XR_002958138,XR_429942,XR_934678	LOC102724715	uncharacterized LOC102724715, transcript variant X1	-1.709072	-3.147359	-2.215371
NM_005266,NM_181703,XM_005272951,XM_017001044	GJA5	gap junction protein alpha 5	-1.060429	-1.448359	-2.216321
NR_037627	LOC100288570	glycosylphosphatidylinositol anchor attachment protein 1 homolog (yeast) pseudogene	-1.992252	-1.340712	-2.217334
NM_007115	TNFAIP6	TNF alpha induced protein 6	-1.441021	-1.299923	-2.218295
XR_001748470,XR_001748471	LOC105369149	uncharacterized LOC105369149, transcript variant X1	-1.084154	-1.868568	-2.219045
NM_001199829,NM_032132,XM_011510054	HORMAD1	HORMA domain containing 1	-1.263365	-1.405592	-2.220132
NM_001195483,NM_024628	SLC12A8	solute carrier family 12 member 8	-2.025636	-1.007783	-2.222776
NM_001278233,NM_001278234,NM_001278235,NM_014583	LMCD1	LIM and cysteine rich domains 1	-2.053106	-1.071664	-2.227291
NM_018557,XM_017004341,XM_017004342,XR_001738778	LRP1B	LDL receptor related protein 1B	-1.967070	-1.336102	-2.227646
NR_033969	PLCE1-AS1	PLCE1 antisense RNA 1	-2.512816	1.486660	-2.228769
NM_032289,XM_011537696,XM_017009976,XM_017009977	PSD2	pleckstrin and Sec7 domain containing 2	-1.963724	-1.668863	-2.230400
NM_019599	TAS2R1	taste 2 receptor member 1	-1.715445	-1.331506	-2.232948
NM_001105543,NM_020911,NM_181775,XM_005250686,XM_006716171,XM_0115166	PLXNA4	plexin A4	-2.257257	1.075858	-2.233121

76,XM_017012779,XR_927546					
NR_024607	MIR503HG	MIR503 host gene	-1.934930	-1.176883	-2.235301
NM_018899,NM_031883	PCDHAC2	protocadherin alpha subfamily C, 2	-1.701975	-1.406602	-2.236140
NM_003107	SOX4	SRY-box transcription factor 4	-2.602110	-1.127503	-2.241362
NM_001205288,NM_001371340,NR_038099,XM_017003103,XM_017003104,XM_017003105	LIMS4	LIM zinc finger domain containing 4	-1.505034	-1.329249	-2.245679
NM_001142749,NM_001291990,NM_001291991,NM_152748,XM_006715894,XM_011515918,XM_011515921,XM_017011843,XM_017011844,XM_024446686	ELAPOR2	endosome-lysosome associated apoptosis and autophagy regulator family member 2	-2.581753	-1.652897	-2.247160
NM_001012662,NM_001012664,NM_001013251,NM_002394,NR_037193	SLC3A2	solute carrier family 3 member 2	-1.986589	-1.498585	-2.247673
NM_001190709,NM_001854,NM_080629,NM_080630,NR_134980,XM_017000334,XM_017000335,XM_017000336,XM_017000337	COL11A1	collagen type XI alpha 1 chain	-1.513273	-1.453368	-2.250030
NM_001277077,NM_001277078,NM_004272,XM_017010059	HOMER1	homer scaffold protein 1	-1.737100	-1.356986	-2.252413
XR_001753445	LOC107987258	uncharacterized LOC107987258	-1.511238	-1.409366	-2.258488

NM_001278919,NM_001278920,NM_030779,NM_173092,XM_011525308,XM_011525309,XM_011525310,XM_011525311,XM_011525312,XM_011525313,XM_017025175,XM_017025176,XM_017025177,XM_017025178,XM_017025179,XM_017025180,XR_934568	KCNH6	potassium voltage-gated channel subfamily H member 6	1.225148	-1.223853	-2.259124
NM_001127891,NM_001302508,NM_001302509,NM_001302510,NM_004530	MMP2	matrix metallopeptidase 2	-1.733913	-1.453472	-2.260527
NM_001128933,NM_001128934,NM_001286754,NM_001286755,NM_133477	SYNPO2	synaptopodin 2	-1.574285	-1.231119	-2.260839
NM_001277742,NM_019885,XM_005264433,XM_011532988	CYP26B1	cytochrome P450 family 26 subfamily B member 1	-1.531514	-2.135668	-2.263772
NM_021165,XM_005245379,XM_011509826,XM_024448722	BRINP2	BMP/retinoic acid inducible neural specific 2	-2.057876	-1.453257	-2.271783
XR_001755529,XR_001755530,XR_938223	LOC105377199	uncharacterized LOC105377199, transcript variant X3	-1.335862	-1.348440	-2.277720
NM_001293179,NM_001293180,NM_007173,NR_120591,NR_120592,NR_120593	PRSS23	serine protease 23	-2.406819	-1.251334	-2.281597
NM_001242350,NM_004665,NM_078488,NR_034173,NR_034174,NR_110143,NR_110144,NR_110145,NR_110146,XM_006715593,XM_011536231,XM_01	VNN2	vanin 2	-2.140010	-1.316679	-2.282543

7011408,XM_017011409,XR_002956311					
NM_001197294,NM_001387,XM_011537574	DPYSL3	dihydropyrimidinase like 3	-2.107493	-1.302927	-2.283142
NM_001256442,NM_001256443,NM_145239,XM_011545715,XM_017022887,XM_017022888,XM_017022889	PRRT2	proline rich transmembrane protein 2	-2.211942	-1.372394	-2.285017
NM_001031700,NM_001128424,NM_016613,XM_024454078,XM_024454079	GASK1B	golgi associated kinase 1B	-2.146128	-1.299068	-2.285144
NM_001135919,NM_001347960,NM_181785,XM_005266361	SLC46A3	solute carrier family 46 member 3	-1.665180	-1.275651	-2.288436
NM_001285829,NM_001287424,NM_001287435,NM_004364	CEBPA	CCAAT enhancer binding protein alpha	-1.805376	-2.045566	-2.289026
NM_030916,XM_005245508,XM_011510021,XM_011510022,XM_011510023	NECTIN4	nectin cell adhesion molecule 4	-2.373706	-1.541661	-2.289567
NM_006043,XM_011546001,XM_011546002	HS3ST2	heparan sulfate-glucosamine 3-sulfotransferase 2	-1.649455	-1.445861	-2.292440
NR_146730,NR_146731	LINC01715	long intergenic non-protein coding RNA 1715	-3.406801	-1.826732	-2.292740
NM_033514,NR_027467	LIMS3	LIM zinc finger domain containing 3	-1.409949	-1.316789	-2.292807
NR_024337	SERTAD4-AS1	SERTAD4 antisense RNA 1	-2.837068	-1.115320	-2.294006
NM_172069,XM_017003351,XM_017003352,XM_017003353,XM_024452695,XR_001738620,XR_001738621,	PLEKHH2	pleckstrin homology, MyTH4 and FERM domain containing H2	-1.363977	-1.614880	-2.297711

XR_001738622,XR_426981,XR_939657					
NM_002193	INHBB	inhibin subunit beta B	-1.867446	-1.139131	-2.299775
NM_005761,NR_037687,XM_006719186,XM_011537730,XM_011537731,XM_017018671,XM_017018672	PLXNC1	plexin C1	-1.668078	-1.353086	-2.305035
NM_005556,XM_011538325,XM_017019294,XR_001748699,XR_001748700	KRT7	keratin 7	-2.210716	-1.262819	-2.310355
NM_022131,XM_017007022	CLSTN2	calsyntenin 2	-1.496026	-1.293197	-2.315063
NM_001378969,NM_001378970,NM_004980,NM_172198,XM_006710629,XM_006710631,XM_006710632,XM_011541425,XM_011541426,XM_011541427,XM_011541428,XM_017001244,XM_017001245	KCND3	potassium voltage-gated channel subfamily D member 3	-1.840533	-1.904292	-2.315785
NM_001099294,XM_005261790	SHISAL1	shisa like 1	-2.762249	-1.398274	-2.319482
NM_181617	KRTAP21-2	keratin associated protein 21-2	-2.472105	1.137402	-2.327511
NR_037182	LOC653513	phosphodiesterase 4D interacting protein-like	-2.390450	-1.431948	-2.330325
NM_001012973,NM_001331125,NR_138551	PLAC9	placenta associated 9	-2.123542	-1.779638	-2.336518
NM_001309443,NM_001309444,NM_003118	SPARC	secreted protein acidic and cysteine rich	-1.565476	-1.277377	-2.339060

NM_001006932,NM_001318936,NM_001318937,NM_001318938,NM_021135,XM_006715549	RPS6KA2	ribosomal protein S6 kinase A2	-1.800425	-1.277459	-2.342817
NM_004975,XM_006723784,XM_011528799	KCNB1	potassium voltage-gated channel subfamily B member 1	-1.585884	1.055290	-2.351643
NM_001258311	PGBD5	piggyBac transposable element derived 5	-3.057225	-1.329564	-2.351820
NM_001313726,NM_001313727,NM_031418,XM_011520282,XM_017018118,XM_017018119	ANO3	anoctamin 3	-1.460925	-1.027077	-2.352090
NM_001365079,NM_014631	SH3PXD2A	SH3 and PX domains 2A	-1.615256	-1.490180	-2.357590
NM_001303420,NM_001303421,NM_015101	COLGALT2	collagen beta(1-O)galactosyltransferase 2	-2.396926	-1.392354	-2.369328
NR_036485	SBF2-AS1	SBF2 antisense RNA 1	-1.277043	-1.779599	-2.378054
NR_015358	LINC00643	long intergenic non-protein coding RNA 643	-2.365307	-1.485190	-2.379547
NR_161374	LOC401478	uncharacterized LOC401478	-5.265410	1.037427	-2.385046
NM_001114396,NM_001351060,NM_001351062,NM_001351063,NM_002262,NM_007334,NR_147038,NR_147039,NR_147040,XM_006719067,XM_011520650,XM_011520651,XM_017019285,XM_017019286,XM_017019287,XM_017019288,X	KLRD1	killer cell lectin like receptor D1	-1.702661	-1.387573	-2.385892

M_017019289,XM_024448974,XR_001748696,XR_001748697					
NM_001324112,NM_004820,XM_017014002	CYP7B1	cytochrome P450 family 7 subfamily B member 1	-1.455462	-1.166055	-2.388021
NM_003469	SCG2	secretogranin II	-1.186349	-1.693174	-2.391282
NM_001278613,NM_001278615,NM_002587,NM_032420,XM_005268452,XM_005268454,XM_005268455,XM_017009517,XM_017009518	PCDH1	protocadherin 1	-1.871478	-1.507244	-2.393431
NR_033937	LOC100132077	uncharacterized LOC100132077	1.040276	-1.166686	-2.393498
NM_002114,XM_011514546,XM_011514547,XM_011514548,XM_011514549,XM_011514550,XM_011514551,XM_011514552,XM_011514553,XM_011514555,XM_017010800,XM_017010801,XM_017010802,XM_017010803,XM_017010804,XR_001743372	HIVEP1	HIVEP zinc finger 1	-1.773359	-1.215063	-2.400736
NR_125967,NR_125968	LINC01719	long intergenic non-protein coding RNA 1719	-1.206157	-1.356821	-2.401426
NM_001346590,NM_001346591,NM_001346592,NM_001346593,NM_001346594,NM_005542,NM_198336,NM_198337	INSIG1	insulin induced gene 1	-1.854271	-1.747578	-2.402348

NM_199346,XM_005264292,XM_006712007,XM_011532823	PFN4	profilin family member 4	-2.037495	-1.992970	-2.410351
NM_000090	COL3A1	collagen type III alpha 1 chain	-1.658042	-1.301872	-2.411601
NM_001142776,NM_024111,XM_024450045,XM_024450046,XM_024450047	CHAC1	ChaC glutathione specific gamma-glutamylcyclotransferase 1	-1.556112	-1.448102	-2.412216
NM_025130,XM_011540195,XR_001747209	HKDC1	hexokinase domain containing 1	-1.994073	-1.321327	-2.422935
NM_000093,NM_001278074,XM_017014266,XR_001746183	COL5A1	collagen type V alpha 1 chain	-1.845562	-1.249688	-2.429304
NM_198353,XM_011513690	KCTD8	potassium channel tetramerization domain containing 8	-2.194796	-2.923626	-2.434740
NM_001255976,NM_020182,NM_199169,NM_199170,NM_199171	PMEPA1	prostate transmembrane protein, androgen induced 1	-2.373971	-1.213825	-2.435467
XR_938114	LOC105372976	uncharacterized LOC105372976	-1.265856	-1.648207	-2.440156
NM_001080529,XM_017012522	WIPF3	WAS/WASL interacting protein family member 3	-3.118528	1.002510	-2.442217
XR_947756	LOC105378936	uncharacterized LOC105378936	-2.170226	-1.513841	-2.456240
NM_001030060,XM_017010850	SAMD5	sterile alpha motif domain containing 5	-2.522407	-1.396941	-2.456709
NM_020690	ANKHD1-EIF4EBP3	ANKHD1-EIF4EBP3 readthrough	1.291213	-1.021394	-2.461436
NM_001169122,NM_001169123,NM_001169124,NM_001169125,NM_001170628,NM_002025	AFF2	AF4/FMR2 family member 2	-3.240890	-1.316734	-2.468640

NM_001007237,NM_001542,XM_005270794,XM_006710593,XM_011541315,XM_011541316	IGSF3	immunoglobulin superfamily member 3	-1.895527	-1.504769	-2.468742
NM_002928,XM_024448796	RGS16	regulator of G protein signaling 16	-2.625677	-1.083197	-2.469769
XR_945388,XR_945389	LOC105370003	uncharacterized LOC105370003, transcript variant X2	-3.187074	-1.193431	-2.472166
NM_001134,NM_001354717	AFP	alpha fetoprotein	-1.734717	-1.123134	-2.476212
NM_020697	KCNS2	potassium voltage-gated channel modifier subfamily S member 2	-1.297779	-1.528271	-2.477556
NM_181726,XM_017008176	ANKRD37	ankyrin repeat domain 37	-1.901385	-1.706412	-2.478966
XR_001743969	LOC107986566	uncharacterized LOC107986566	-1.014992	1.050153	-2.484104
NM_001145453,NM_001348096,NM_001348098,NM_001348099,NM_001382556,NM_001382557,NM_001382558,NM_001382559,NM_001382560,NM_001382561,NM_005264,NM_145793,XM_011539634	GFRA1	GDNF family receptor alpha 1	-2.473939	-1.010002	-2.489763
NR_135290	GCC2-AS1	GCC2 antisense RNA 1	-2.198102	-1.403029	-2.504454
NR_037868	SLFNL1-AS1	SLFNL1 antisense RNA 1	-2.166230	-1.785673	-2.505684
NR_026974	ZNF252P-AS1	ZNF252P antisense RNA 1	-1.742639	-2.766187	-2.507264

NR_102710,NR_102711	XXYLT1-AS2	XXYLT1 antisense RNA 2	-1.488078	-1.439685	-2.507462
NM_001382000,NM_014695,NR_130142,NR_167766,XM_017025429,XM_017025430,XM_024451045,XM_024451046,XR_001752702,XR_001752703,XR_001752704	CCDC144A	coiled-coil domain containing 144A	-1.708787	-1.019636	-2.511413
XR_001737772	LOC107985599	uncharacterized LOC107985599	-3.978538	-2.431922	-2.512207
NM_001253693,NM_018298,XM_005271003,XM_006710750,XM_011541739,XM_011541740,XM_011541741,XM_011541742,XR_946699	MCOLN3	mucolipin 3	-2.144963	-1.500926	-2.513787
NM_001329630,NM_001329631,NM_175058,XM_017017241,XM_017017242,XM_024448356,XM_024448357,XM_024448358,XM_024448359,XM_024448360,XM_024448361,XM_024448362,XM_024448363,XM_024448364,XM_024448365,XM_024448366,XM_024448367,XM_024448368,XM_024448369,XM_024448370,XR_002957126	PLEKHA7	pleckstrin homology domain containing A7	-3.037379	-1.388391	-2.514082
NM_001305581,NM_032024,NR_131178	LRMDA	leucine rich melanocyte differentiation associated	-1.717892	1.085424	-2.515547

NR_130726	LINC00243	long intergenic non-protein coding RNA 243	-1.455077	1.099885	-2.522852
XR_001739239,XR_001739240	LOC102723825	uncharacterized LOC102723825, transcript variant X1	-2.058303	-1.713614	-2.523694
NM_005257	GATA6	GATA binding protein 6	-1.778821	-1.265687	-2.528968
NM_006469,XM_005244843,XM_011509079,XM_011509080,XM_017000084	IVNS1ABP	influenza virus NS1A binding protein	-2.210322	-1.420148	-2.540224
NM_000827,NM_001114183,NM_001258019,NM_001258020,NM_001258021,NM_001258022,NM_001258023,NM_001364165,NM_001364166,NM_001364167,NR_047578,NR_157093,XM_017009392	GRIA1	glutamate ionotropic receptor AMPA type subunit 1	-1.438024	1.048431	-2.542307
NR_147989	CZ1P-ASNS	CZ1P-ASNS readthrough	-1.645197	1.648495	-2.544522
NM_001135599,NM_003238,NR_138148,NR_138149	TGFB2	transforming growth factor beta 2	-1.956671	-1.143684	-2.544980
NM_001271751,NM_001369434,NM_001369435,NM_005795,XM_005246232,XM_005246234	CALCRL	calcitonin receptor like receptor	-2.543008	-1.627258	-2.555240
NR_110806	LINC01969	long intergenic non-protein coding RNA 1969	-2.248208	-1.629362	-2.562435
NR_015375	VLDLR-AS1	VLDLR antisense RNA 1	-2.885450	-1.180795	-2.565930
NM_001105077,NM_001105078,NM_001163	MECOM	MDS1 and EVI1 complex locus	-2.497104	-1.878495	-2.572783

999,NM_001164000,NM_001205194,NM_001366466,NM_001366467,NM_001366468,NM_001366469,NM_001366470,NM_001366471,NM_001366472,NM_001366473,NM_001366474,NM_004991,NM_005241,XM_005247213,XM_005247214,XM_005247221,XM_005247224,XM_005247225,XM_011512546,XM_017005874,XM_017005877					
NM_020179,XM_011542907,XM_011542909,XM_011542910,XM_011542911,XM_017018019,XM_017018020	SMCO4	single-pass membrane protein with coiled-coil domains 4	-2.462104	-1.666891	-2.572800
NM_002240	KCNJ6	potassium inwardly rectifying channel subfamily J member 6	-1.079132	-1.560396	-2.575871
NR_002594	SLC7A5P2	solute carrier family 7 member 5 pseudogene 2	-1.388563	-1.101706	-2.577309
NM_001164104,NM_001164105,NM_001164106,NM_018995,XM_005261923,XM_011530696,XM_011530697,XM_011530698,XM_011530699,XM_011530700,XM_011530701,XM_011530702,XM_011530703,XM_011530704,XM_017028833,XM_017028834,XM_017028835,XM_0170288	MOV10L1	Mov10 like RISC complex RNA helicase 1	-1.876327	-1.735658	-2.580851

36,XM_017028837,XR_002958704					
NM_198182,XM_005246159,XM_006711882,XM_006711884,XM_011510343,XM_017003900,XM_017003901,XR_001738726,XR_001738727,XR_001738728	GRHL1	grainyhead like transcription factor 1	-1.786561	-1.820713	-2.583527
XR_001755541,XR_938256	LOC105373032	uncharacterized LOC105373032, transcript variant X2	-2.454903	-1.636509	-2.590291
NM_015719,XM_011528042,XM_017026849	COL5A3	collagen type V alpha 3 chain	-1.863077	-1.848438	-2.595586
NR_040058	RAD51-AS1	RAD51 antisense RNA 1	-1.437694	-1.506287	-2.598920
NM_014585,XM_005246505,XM_017003938	SLC40A1	solute carrier family 40 member 1	-2.192363	-1.888196	-2.600401
NM_014737,NM_170774,XM_005260895,XM_011529410,XM_011529411,XM_011529412,XM_017028149,XM_017028150,XM_017028151,XM_017028152,XM_017028153	RASSF2	Ras association domain family member 2	-1.584509	-1.994228	-2.607455
NM_032246	MEX3B	mex-3 RNA binding family member B	-2.481362	-1.320716	-2.622732
XM_011513341	LOC105374013	endogenous retrovirus group K member 5 Gag polyprotein	-1.986444	-1.813109	-2.623188
NM_001112800,NM_001112801,NM_001112802,NM_001252624,NM_001351483,NM_001351484,NM_0013514	SLC8A1	solute carrier family 8 member A1	-1.909134	-1.066687	-2.626330

85,NM_001351486,NM_001351487,NM_001351488,NM_001351489,NM_001351490,NM_001351491,NM_001351492,NM_001351493,NM_001351494,NM_001372263,NM_021097,XM_006712082,XM_006712083,XM_006712084,XM_006712085,XM_011533050,XM_011533054,XM_011533055,XM_011533056,XM_011533058,XM_017004745,XM_017004746,XM_017004749,XM_017004750,XM_017004751,XM_017004752,XM_017004753,XM_017004755,XM_017004764					
NM_001350120,NM_001350121,NM_006379	SEMA3C	semaphorin 3C	-1.800632	-1.419329	-2.631132
NR_024008	WWC2-AS2	WWC2 antisense RNA 2	-2.073361	-1.103269	-2.631324
NM_001371783,NM_198461,XR_002959247	LONRF2	LON peptidase N-terminal domain and ring finger 2	-1.683602	-2.265432	-2.632267
NM_001308315,NM_001308316,NM_005725,XM_016999996	TSPAN2	tetraspanin 2	-2.946657	-1.088354	-2.632816
NM_014244,NM_021599	ADAMTS2	ADAM metallopeptidase with thrombospondin type 1 motif 2	-2.188685	-1.251393	-2.633268
XR_943077	LOC105378029	uncharacterized LOC105378029	-2.030300	-1.372475	-2.646319

NM_001202439,XM_011520072,XM_011520073,XM_011520074,XM_011520075	NCR3LG1	natural killer cell cytotoxicity receptor 3 ligand 1	-1.695658	-1.524632	-2.653727
NR_003051	RMRP	RNA component of mitochondrial RNA processing endoribonuclease	-2.333685	-1.844991	-2.655337
XR_243478,XR_429779,XR_933760,XR_933761	LOC101928230	uncharacterized LOC101928230, transcript variant X4	-1.805110	-1.732028	-2.669775
NM_130767,XM_006714531,XM_006714532,XM_017009045,XM_017009046,XM_017009047,XM_017009048,XM_024454366	ACOT12	acyl-CoA thioesterase 12	-2.710957	-1.617307	-2.671014
NM_031476,XM_005256190,XM_024450463	CRISPLD2	cysteine rich secretory protein LCCL domain containing 2	-2.212429	-1.478382	-2.675180
NM_001174071,NM_001174072,NM_178276,NR_126060,NR_126061,XM_011543304,XM_017009323,XM_017009324,XM_017009326,XM_024446013	SERINC5	serine incorporator 5	-2.071744	-1.786193	-2.675975
NM_001114734,NM_001363585,XR_001741133,XR_001741134,XR_001741135,XR_001741136,XR_001741137,XR_001741138,XR_001741139	PABPC4L	poly(A) binding protein cytoplasmic 4 like	-4.048386	-1.761105	-2.681130
NR_038399,NR_138541,NR_138542	FILNC1	FOXO induced long non-coding RNA 1	-1.151015	-2.319576	-2.684189
NM_012342	BAMBI	BMP and activin membrane bound inhibitor	-2.655823	-1.278457	-2.688623

NM_020700,XM_011538578,XM_011538579,XM_017019676	PPM1H	protein phosphatase, Mg2+/Mn2+ dependent 1H	-2.056071	-1.346180	-2.696332
NM_001135934,NM_001135935,NM_001135936,NM_001286665,NM_001286666,NM_001286667,NM_001330517,NM_006475,XM_005266232,XM_017020355,XM_017020356	POSTN	periostin	-1.840356	-1.345670	-2.702380
NM_001242463,NM_058229,NM_148177	FBXO32	F-box protein 32	-2.408854	-1.446091	-2.710831
XR_001753371,XR_935292,XR_935293	LOC105372026	uncharacterized LOC105372026, transcript variant X2	-1.526725	1.211802	-2.716361
NM_001130455,NM_001130976,NM_001130977,NM_001130978,NM_001130979,NM_001130980,NM_001130981,NM_001130982,NM_001130983,NM_001130984,NM_001130985,NM_001130986,NM_001130987,NM_003494,XM_005264584,XM_005264585,XR_001738969	DYSF	dysferlin	-2.898858	-1.997541	-2.716790
NM_001018073,NM_001291556,NM_001308054,NM_004563,XM_006720158	PCK2	phosphoenolpyruvate carboxykinase 2, mitochondrial	-2.291367	-1.519436	-2.717017
NM_005252	FOS	Fos proto-oncogene, AP-1 transcription factor subunit	-2.044894	-2.216126	-2.720856
NR_110681	LOC101927604	uncharacterized LOC101927604	-1.979733	-1.608875	-2.722208

NM_001142569,NM_001367289,NM_001367290,NM_018265,XM_011509754,XM_011509755,XM_011509756	INAVA	innate immunity activator	-1.547933	-1.089905	-2.726625
XR_001746706	LOC107984011	uncharacterized LOC107984011	-2.118661	-1.301745	-2.728293
NM_004794,XM_017029963	RAB33A	RAB33A, member RAS oncogene family	-2.535817	-1.518568	-2.735072
NM_001300965,NM_001321	CSRP2	cysteine and glycine rich protein 2	-2.427096	-1.552188	-2.735658
NM_138573,XM_017021937,XM_017021938,XM_017021939,XM_017021940,XM_017021941,XM_017021942,XM_017021943,XM_017021944,XM_017021945,XM_017021946,XM_017021947,XM_017021948,XM_024449847,XM_024449848	NRG4	neuregulin 4	-2.611304	-1.908145	-2.737542
NM_000253,NM_001300785	MTTP	microsomal triglyceride transfer protein	-1.557875	-1.715495	-2.737760
XR_948811	CTB-1I21.1	uncharacterized CTB-1I21.1	-2.098131	-1.426844	-2.739212
XR_946382,XR_946383,XR_946384,XR_946385	LOC105378525	uncharacterized LOC105378525, transcript variant X3	-2.873663	-2.277857	-2.742576
NM_001287491,NM_001366022,XM_011532682,XM_011532683,XM_011532684,XM_011532685,XM_011532686,XM_011532687,XM_011532688,XM_011532690,XM_0170035	TET3	tet methylcytosine dioxygenase 3	-1.858221	-1.328862	-2.751050

66,XM_024452745,XM_024452746,XM_024452747					
NR_105059,NR_105060	CARMN	cardiac mesoderm enhancer-associated non-coding RNA	-1.754610	-1.056516	-2.755294
NM_001363487,NM_145008	YPEL4	yippee like 4	-2.758321	-1.514043	-2.758852
gene-ANKRD30BP1	ANKRD30BP1	ankyrin repeat domain 30B pseudogene 1	-1.888261	1.078736	-2.765422
NM_001127384,NM_001291133,NM_013266,XM_017016151,XM_017016152,XM_017016153,XM_017016154,XM_017016155,XM_017016156,XM_017016157,XM_017016158	CTNNA3	catenin alpha 3	-2.944221	-1.227632	-2.771524
XR_001753953,XR_001753954	LOC107985305	uncharacterized LOC107985305, transcript variant X1	1.033450	-1.480472	-2.776124
NR_046110,NR_046111,NR_046112	LINC01123	long intergenic non-protein coding RNA 1123	-2.278932	-1.986126	-2.780484
XR_001754477	LOC107985414	uncharacterized LOC107985414	-1.579677	-1.584024	-2.780701
NM_001278642,NM_005711	EDIL3	EGF like repeats and discoidin domains 3	-2.349531	-1.146461	-2.781177
NR_109767	LOC100131315	uncharacterized LOC100131315	-1.573469	-2.389041	-2.782161
gene-RPL7AP16	RPL7AP16	ribosomal protein L7a pseudogene 16	-1.232658	-1.947961	-2.787704
NM_001378891,NM_001378902,NM_002944,XM_006715548,XM_011536049,XM_011536050,XM_011536051,	ROS1	ROS proto-oncogene 1, receptor tyrosine kinase	-2.117805	-1.436765	-2.788814

XM_011536053,XM_011536054,XM_011536055,XM_011536056,XM_011536057,XM_011536058,XM_017011172,XM_017011173					
NM_001320996,NM_004102,XM_011541007	FABP3	fatty acid binding protein 3	-2.054185	-2.681795	-2.789238
NM_020142	NDUFA4L2	NDUFA4 mitochondrial complex associated like 2	-2.398580	-1.153488	-2.789649
NM_152754,XM_011515960,XM_011515961,XM_017011873	SEMA3D	semaphorin 3D	-1.596161	-1.578399	-2.796352
NR_151491	NMRAL2P	NmrA like redox sensor 2, pseudogene	-3.060260	-1.517294	-2.802256
NM_001005242,NM_004572	PKP2	plakophilin 2	-2.522266	-1.191856	-2.807128
NR_033651,NR_033652	MSC-AS1	MSC antisense RNA 1	-2.769544	-1.412016	-2.808941
NM_003619,XM_005263318,XM_011532387	PRSS12	serine protease 12	-2.118909	-1.364809	-2.809786
NM_001448	GPC4	glypican 4	-2.500903	-1.365588	-2.812267
NR_004847	UBE2Q2P2	ubiquitin conjugating enzyme E2 Q2 pseudogene 2	-2.667761	-1.671527	-2.821691
NM_183373,XM_011514393,XR_001743252	PXDC1	PX domain containing 1	-2.187376	-1.358869	-2.823128
NM_024490,XM_005268261,XM_011521826,XM_011521828,XM_011521829,XM_011521830,XM_017022436,XM_017022437,XR_0	ATP10A	ATPase phospholipid transporting 10A (putative)	-2.706618	-1.372243	-2.837910

01751366,XR_001751367,XR_001751368					
XR_001739683	LOC107985942	uncharacterized LOC107985942	-1.481492	-1.375462	-2.838826
NM_015351	TTC9	tetratricopeptide repeat domain 9	-2.544862	-1.537570	-2.842055
NM_001303110,NM_001845,XM_011521048	COL4A1	collagen type IV alpha 1 chain	-1.787643	-1.371072	-2.844856
NM_012098,XM_006717030	ANGPTL2	angiopoietin like 2	-2.389408	-1.685247	-2.848317
NM_001292049,NM_003149,XM_011534037,XM_011534038,XM_017007083,XM_024453722,XM_024453723,XR_940496	STAC	SH3 and cysteine rich domain	-2.361595	-1.453965	-2.862703
NM_001278579,NM_001278580,NM_001616	ACVR2A	activin A receptor type 2A	-2.200795	-1.353713	-2.876457
NM_001282773,NM_001282775,NM_001282778,NM_001350113,NM_001350114,NM_001350115,NM_001350116,NM_001364886,NM_001374806,NM_001374807,NM_001374808,NM_001374809,NM_001374810,NM_001374811,NM_001374812,NM_001374813,NM_001374814,NM_001374815,NM_001374816,NM_002924,XM_006711800,XM_011544246,XM_011544247,XM_017002001,XM_017002002,XM_0170020	RGS7	regulator of G protein signaling 7	-2.403358	-1.164280	-2.877641

05,XM_017002009,XM_017002011,XM_017002012,XM_017002013					
NM_001711,XM_017029724	BGN	biglycan	-2.621782	-1.588926	-2.878281
NR_135738	GTF2IP23	general transcription factor IIi pseudogene 23	-1.643067	-1.083309	-2.883453
NR_132969,NR_132970,NR_132971	LOC145694	uncharacterized LOC145694	-1.846950	-1.878522	-2.900540
NM_001168368,NM_020692,XM_011537005,XM_011537006,XM_011537007,XM_017021498	GALNT16	polypeptide N-acetylgalactosaminyltransferase 16	-3.960904	-1.394358	-2.902060
NM_015000,XM_005253342,XM_006719058,XM_006719059,XM_011520613,XM_024448889,XM_024448890,XM_024448891,XM_024448892,XM_024448893,XM_024448894,XM_024448895,XM_024448896,XM_024448897,XR_001748626	STK38L	serine/threonine kinase 38 like	-2.205198	-1.642954	-2.903245
NR_164111	LMO7DN	LMO7 downstream neighbor	-1.813442	-1.708103	-2.909886
NM_001317184,NM_001317185,NM_001317186,NM_004360	CDH1	cadherin 1	-1.372767	-1.399597	-2.911172
XR_932532	LOC105370924	uncharacterized LOC105370924	-2.164750	-1.196147	-2.916449
NM_001029851,NM_001029852,NM_001029853,NM_001029854,NM_001349748,NM_00	PDE8B	phosphodiesterase 8B	-1.576899	-1.523298	-2.944585

1349749,NM_001349750,NM_001349751,NM_001349752,NM_001349753,NM_001376062,NM_001376063,NM_001376064,NM_001376065,NM_001376066,NM_001376067,NM_001376068,NM_001376069,NM_001376070,NM_001376071,NM_001376072,NM_001376073,NM_001376074,NM_001376075,NM_003719,XM_006714726,XM_011543699,XM_011543700,XM_011543704					
NM_001005407,NM_021098,XM_005255652,XM_006720963,XM_006720964,XM_006720965,XM_006720967,XM_006720968,XM_011522724,XM_011522727,XM_017023819,XM_017023820,XM_017023821,XR_002957850	CACNA1H	calcium voltage-gated channel subunit alpha1 H	-2.014227	-2.465702	-2.964332
NM_001409,XM_006710406,XM_011540885,XM_011540886,XM_011540887,XM_011540888,XM_017000533	MEGF6	multiple EGF like domains 6	-2.046908	-1.519416	-2.970158
NM_080671	KCNE4	potassium voltage-gated channel subfamily E regulatory subunit 4	-2.134883	-1.602476	-2.988613
NM_022469,XM_005273226,XM_011544249	GREM2	gremlin 2, DAN family BMP antagonist	-2.334503	-2.221507	-2.997676

NM_001142474,NM_001142475,NM_001142476,NM_001142477,NM_001142478,NM_001142479,NM_001142480,NM_001142481,NM_001142482,NM_001142483,NM_004772,XM_006714732,XM_006714733	NREP	neuronal regeneration related protein	-2.211108	-1.131284	-3.001672
NM_001003683,NM_001258312,NM_001258313,NM_001258314,NM_001363871,NM_005019,XM_011511323,XM_011511324,XM_011511325,XM_011511326,XM_017004294,XM_017004295,XM_017004296,XM_017004297,XM_017004298,XM_017004299,XM_017004300,XM_017004301,XM_024452959,XR_001738769,XR_002959304	PDE1A	phosphodiesterase 1A	-2.908640	-1.712314	-3.023576
NM_001195610,NM_016356	DCDC2	doublecortin domain containing 2	-2.319838	-1.937069	-3.032794
NR_002836	PGM5P2	phosphoglucomutase 5 pseudogene 2	-1.530283	-1.291340	-3.037253
NR_027118,NR_027119	INHBA-AS1	INHBA antisense RNA 1	-3.756017	-1.004836	-3.040329
NM_001287444,NM_001365580,XM_006711894,XM_017004836,XM_017004837,XR_001738920,XR_001738921	DCDC2C	doublecortin domain containing 2C	-3.592245	-1.512220	-3.049480

NR_120598	GACAT2	gastric cancer associated transcript 2	-3.205279	-1.433733	-3.051085
NM_001288973,NM_001288974,NM_001288975,NM_003474,NM_021641,XM_017016705,XM_017016706,XM_024448210	ADAM12	ADAM metallopeptidase domain 12	-2.037925	-1.419053	-3.075328
NM_001004439,XM_005254228,XM_011521363	ITGA11	integrin subunit alpha 11	-2.187622	-1.717666	-3.083996
NM_021186	ZP4	zona pellucida glycoprotein 4	-2.821075	1.184130	-3.099099
NM_004405	DLX2	distal-less homeobox 2	-2.632691	-1.724932	-3.100920
gene-XPOTP1	XPOTP1	exportin for tRNA pseudogene 1	-1.016393	-1.467134	-3.107160
NM_001371457,NM_002410,XM_005263669,XM_005263670,XM_006712534,XM_011511199,XM_011511200,XM_011511201,XM_011511202,XM_011511204,XM_017004147,XM_017004148,XM_017004149	MGAT5	alpha-1,6-mannosylglycoprotein 6-beta-N-acetylglucosaminyltransferase	-1.744932	-1.792136	-3.107495
NM_001173523,NM_002589,NM_032456,NM_032457,XM_005248163,XM_005248164,XM_005248166,XM_011513842,XM_011513843,XM_011513844,XM_017008259,XM_017008260,XM_017008261,XM_017008262,XM_017008263,XM_01	PCDH7	protocadherin 7	-2.443069	-1.362860	-3.121601

7008264,XM_017008265,XM_017008266,XM_017008267,XM_017008268,XM_017008269,XM_017008271,XM_017008272					
NM_001017961,NM_001320302,NR_135199,NR_163271,XM_017000417	FAM78B	family with sequence similarity 78 member B	-1.765127	-1.563594	-3.122001
NM_001040167,NM_001040168,NM_001166355,NM_002304	LFNG	LFNG O-fucosylpeptide 3-beta-N-acetylglucosaminyltransferase	-3.092361	-1.376366	-3.170380
NM_001172651,NM_003451	ZNF177	zinc finger protein 177	-1.346091	1.047948	-3.177059
NM_001354173,NM_001375428,NM_019605,XR_921894	SERTAD4	SERTA domain containing 4	-3.022136	-1.058912	-3.182508
NM_001901	CCN2	cellular communication network factor 2	-2.322388	-1.492730	-3.194285
NR_027142,NR_027143	LOC440895	two pore channel 3 pseudogene	-1.433404	-2.025733	-3.235603
NR_125715	TGFB2-OT1	TGFB2 overlapping transcript 1	-2.531554	-2.545292	-3.244824
NM_001009554,NM_001301647,NM_001301648,NM_021647,NR_125890,XM_005263366,XM_005263368,XM_017008864,XM_017008865,XM_017008866,XM_017008867,XM_017008868,XM_017008869,XM_017008870,XM_017008871	MFAP3L	microfibril associated protein 3 like	-3.234892	-1.315806	-3.293530

NM_001098517,NM_001301043,NM_001301044,NM_001301045,NM_014333,XM_005271494,XM_017017457	CADM1	cell adhesion molecule 1	-2.254726	-1.395889	-3.317704
NR_003334	SNORD116-20	small nucleolar RNA, C/D box 116-20	1.038188	1.087741	-3.318427
NM_018903,NM_031864	PCDHA12	protocadherin alpha 12	-1.518456	1.062242	-3.325564
NM_024870,NM_025170,XM_011517612	PREX2	phosphatidylinositol-3,4,5-trisphosphate dependent Rac exchange factor 2	-2.488039	-1.289434	-3.326528
NM_021965,XM_011518782,XM_011518783,XM_024447573	PGM5	phosphoglucomutase 5	-2.739814	-1.391952	-3.332603
NM_001126336,NM_001164097,NM_001164098,NM_004385	VCAN	versican	-2.488533	-1.327014	-3.340307
NM_001376763,NM_004673,XR_002957991,XR_241102	ANGPTL1	angiopoietin like 1	-4.841830	-1.403420	-3.370472
XR_002959410	LOC112268421	uncharacterized LOC112268421	-3.064193	-1.451091	-3.376832
NM_001171020,NM_152423,XM_005262077,XM_011530856,XM_011530857,XM_017029269,XM_024452334	PWWP3B	PWWP domain containing 3B	-2.630536	-2.047129	-3.389577
NM_001291702,NM_001291703,NM_001368149,NM_001368150,NM_001368151,NM_001368152,NM_001368154,NM_001368155,NM_001368156	VNN3	vanin 3	-1.507939	-2.063906	-3.395118

NM_001004733	OR5B12	olfactory receptor family 5 subfamily B member 12	-1.952826	-2.983936	-3.413622
NM_001135608,NM_001349547,NM_015071,NR_146198,XM_005268398,XM_005268399,XM_005268402,XM_006714774,XM_011537610,XM_011537611,XM_017009247,XM_017009248,XM_017009249,XM_017009250,XR_001742044,XR_001742045,XR_001742046,XR_001742047,XR_001742048,XR_002956154,XR_002956155,XR_944311,XR_944312,XR_944313,XR_944314,XR_944315,XR_944317,XR_944319,XR_944320,XR_944321,XR_944323	ARHGAP26	Rho GTPase activating protein 26	-2.596414	-1.635792	-3.419251
NM_007365,XM_017000148,XR_001736944	PADI2	peptidyl arginine deiminase 2	-3.606453	-1.939602	-3.423628
NM_005285	NPBWR1	neuropeptides B and W receptor 1	-2.710082	-1.808764	-3.435261
NM_020722,XM_005265752,XM_005265755,XM_005265756,XM_006714045,XM_011534397,XM_017008473,XM_017008474,XM_024454158,XM_024454159	CRACD	capping protein inhibiting regulator of actin dynamics	-3.299960	-1.551425	-3.464822
NM_000104	CYP1B1	cytochrome P450 family 1 subfamily B member 1	-2.151316	-1.954206	-3.477550

NR_103839	FRY-AS1	FRY antisense RNA 1	-1.776164	-1.680240	-3.480826
NM_001300828,NM_019035,XM_006714239,XM_017008311	PCDH18	protocadherin 18	-1.984184	-1.794671	-3.481006
XR_001742942	LOC107986467	uncharacterized LOC107986467	-2.934445	-2.144094	-3.484072
NM_001167674,NM_001167675,NM_001256502,NM_001256503,NM_001256504,NM_001256505,NM_001375960,NM_001375961,NM_001375964,NM_001375967,NM_001375968,NM_001381963,NM_001381964,NM_153184,XM_017006062,XM_017006063	CADM2	cell adhesion molecule 2	-5.722311	-2.074331	-3.490728
NM_001347887,NM_001347888,NM_022140,NR_144931,XM_011543531,XM_011543532,XM_011543533,XM_017009689,XR_001742173	EPB41L4A	erythrocyte membrane protein band 4.1 like 4A	-1.872776	-1.080441	-3.491466
XR_001743932	LOC107986558	uncharacterized LOC107986558	-2.592897	-1.140893	-3.491884
NM_014438,NM_173178,XM_011510962	IL36B	interleukin 36 beta	-3.343775	-3.345962	-3.497150
gene-RPS16P10	RPS16P10	ribosomal protein S16 pseudogene 10	-6.814900	-1.031216	-3.507957
NM_001079520,NM_016651,NR_046093,NR_046095,NR_165650,NR_165651,NR_165652	DACT1	dishevelled binding antagonist of beta catenin 1	-3.242227	-1.257598	-3.523004

NM_001198695,NM_002404	MFAP4	microfibril associated protein 4	-2.760227	-1.651622	-3.523581
NM_023037,XM_006719749,XM_017020305,XM_017020306,XM_017020307	FRY	FRY microtubule binding protein	-2.027427	-1.717875	-3.533814
NM_001178075,NM_001178076,NM_001178077,NM_001352496,NM_001673,NM_133436,NM_183356	ASNS	asparagine synthetase (glutamine-hydrolyzing)	-2.616546	-1.413483	-3.549313
NM_001366122,NM_199349,XM_017012184,XM_017012186,XM_017012187,XM_017012188,XM_017012189,XM_017012190,XM_017012191,XM_017012192,XM_017012193,XM_017012194	KCP	kielin cysteine rich BMP regulator	-2.928398	-2.234092	-3.556360
NM_006308	HSPB3	heat shock protein family B (small) member 3	-2.089713	-1.327269	-3.596400
NM_001321708,NM_001321709,NM_001321710,NM_004717,XM_017012780,XM_017012788	DGKI	diacylglycerol kinase iota	-2.354997	-1.446363	-3.596822
NM_001172684,NM_001172685,NM_052840	CELF6	CUGBP Elav-like family member 6	-2.416917	-1.994114	-3.623110
NR_135198	LOC729652	uncharacterized LOC729652	-2.254817	-2.084213	-3.625887
XR_001739409	LOC107985869	uncharacterized LOC107985869	-2.408777	-1.562224	-3.636154
NM_001316676,NM_001316677,NM_001323354,NM_001323355,NM_001323356,NM_00	PTPRE	protein tyrosine phosphatase receptor type E	-1.720596	-1.633402	-3.636282

1323357,NM_006504,NM_130435,XM_005252691,XM_011539994,XM_011539995,XM_011539996,XM_011539998,XM_017016467,XM_017016468,XM_017016469,XM_024448092,XM_024448093,XR_002956995,XR_002956996					
NM_001136528,NM_001136530,NM_006216,NR_073116,XM_005246641,XM_017004330,XM_017004332	SERPINE2	serpin family E member 2	-2.228794	-1.546406	-3.645202
NM_005242,XM_017009223	F2RL1	F2R like trypsin receptor 1	-1.542149	-1.884707	-3.647768
NM_001284355,NM_001321766,NM_133463,XM_011515150,XM_011515151,XM_017011774,XM_017011775,XM_017011776	AMZ1	archaelysin family metallopeptidase 1	-2.382384	-1.439155	-3.660010
NM_005098	MSC	musculin	-2.774205	-1.582744	-3.693082
NM_003246,XM_011521971	THBS1	thrombospondin 1	-2.352345	-1.623443	-3.703813
NM_001024845,NM_001261380,NM_001328626,NM_001328627,NM_001328628,NM_001328629,NM_001328630,NM_006934,NM_201649,NR_048548,XM_011542017,XM_017002152,XM_017002153,XM_024449295	SLC6A9	solute carrier family 6 member 9	-2.578834	-1.574090	-3.714678

XR_001751487,XR_001751488,XR_932129,XR_932130	LOC105370777	uncharacterized LOC105370777, transcript variant X3	-1.789041	-2.255161	-3.721910
NM_001293298,NM_001293304,NM_018689,XM_024450001	CEMIP	cell migration inducing hyaluronidase 1	-2.239717	-1.496377	-3.743651
NM_001037132,NM_001193582,NM_001193583,NM_001193584,NM_001371119,NM_001371122,NM_001371123,NM_001371124,NM_001371125,NM_001371126,NM_001371127,NM_001371128,NM_001371129,NM_001371130,NM_001371131,NM_001371132,NM_001371133,NM_001371134,NM_001371135,NM_001371136,NM_001371137,NM_001371138,NM_001371139,NM_001371140,NM_001371141,NM_001371142,NM_001371143,NM_001371144,NM_001371145,NM_001371146,NM_001371147,NM_001371148,NM_001371149,NM_001371150,NM_001371151,NM_001371152,NM_001371153,NM_001371154,NM_001371155,NM_001371156,NM_001371157,NM_001371158,NM_001371159,NM_001371160,NM_001371161,NM_00	NRCAM	neuronal cell adhesion molecule	-3.403954	-1.619523	-3.744386

1371162,NM_0013711 63,NM_001371164,N M_001371165,NM_00 1371166,NM_0013711 67,NM_001371168,N M_001371169,NM_00 1371170,NM_0013711 71,NM_001371172,N M_001371173,NM_00 1371174,NM_0013711 75,NM_001371176,N M_001371177,NM_00 1371178,NM_0013711 79,NM_001371180,N M_001371181,NM_00 1371182,NM_005010, NR_163867,NR_16386 8,NR_163869,NR_163 870,NR_163871,XM_0 06716003,XM_006716 007,XM_006716012,X M_006716014,XM_01 1516253,XM_0115162 55,XM_011516257,X M_011516258,XM_01 1516259,XM_0115162 61,XM_011516262,X M_011516265,XM_01 1516266,XM_0115162 67,XM_011516268,X M_011516269,XM_01 1516270,XM_0170122 36,XM_017012238,X M_017012239,XM_01 7012246,XM_0170122 47,XM_017012248,X M_017012251,XM_02 4446773,XM_0244467 75,XM_024446777,X M_024446778,XM_02

4446779,XM_024446780					
XR_001753444	LOC107985147	uncharacterized LOC107985147	-1.192335	-1.285023	-3.780282
NM_001080421,XM_011527810,XM_011527811,XM_017026502	UNC13A	unc-13 homolog A	-1.491979	-2.201258	-3.780617
NM_001308353,NM_005855,XM_017003152,XM_017003153,XM_017003154,XM_017003155,XM_017003156	RAMP1	receptor activity modifying protein 1	-2.654189	-2.142080	-3.823650
NM_002010,XM_011534996	FGF9	fibroblast growth factor 9	-2.858257	-1.866644	-3.846591
gene-LOC389473	LOC389473	chromosome 5 open reading frame 13 pseudogene	-1.645589	-1.033163	-3.850993
NM_014331,XM_011531802,XR_001741190,XR_001741191	SLC7A11	solute carrier family 7 member 11	-2.339777	-1.712587	-3.861570
NM_001025108,NM_002285,XM_005263943,XM_005263945,XM_011511169,XM_011511170,XM_011511171,XM_011511173,XM_011511174,XM_011511175,XM_011511176,XM_011511177,XM_011511178,XM_011511179,XM_017004085,XM_017004086,XM_017004087,XM_024452883	AFF3	AF4/FMR2 family member 3	-2.947777	-1.659206	-3.867081
NR_120367	LINC01583	long intergenic non-protein coding RNA 1583	-2.737346	-1.269573	-3.889083

NM_001278585,NM_001278586,NM_006587	CORIN	corin, serine peptidase	-2.288321	-1.663623	-3.890785
NM_001145670,NM_001145671,NM_001145672,NM_001145673,NM_001145674,NM_001145675,NM_001270771,NM_003603,NM_021069,XM_005263307,XM_005263308,XM_005263310,XM_005263311,XM_005263312,XM_005263313,XM_006714388,XM_006714390,XM_017008738,XM_017008739,XM_017008740,XM_017008741,XM_017008743,XM_017008744,XM_017008748,XM_017008750,XM_017008751,XM_017008752,XM_017008753,XM_017008754,XM_017008755,XM_017008756,XM_017008757,XM_017008758,XM_017008759,XM_017008760,XM_017008761,XM_017008762,XM_017008763,XM_017008764,XM_017008765,XM_017008766,XM_017008767,XM_017008768,XM_017008769,XM_017008770,XM_017008771,XM_017008772,XM_017008773,XM_017008774,XM_017008775,XM_024454257,XM_02	SORBS2	sorbin and SH3 domain containing 2	-4.238178	-1.560085	-3.896593

4454258,XM_024454259,XM_024454260,XR_002959769					
NM_001253845,NM_001369882	ADM2	adrenomedullin 2	-2.308581	-1.417641	-3.900573
NM_001304347,NM_001304348,NM_058164	OLFM2	olfactomedin 2	-2.344650	-2.076145	-3.905157
NM_003014	SFRP4	secreted frizzled related protein 4	-2.442184	-1.794608	-3.906798
XR_946386	LOC105378526	uncharacterized LOC105378526	-1.821921	-3.131252	-3.911919
NM_001283012,NM_022783	DEPTOR	DEP domain containing MTOR interacting protein	-3.152455	-1.785448	-3.924889
NM_001308176,NM_001792,XM_011525788,XM_017025514	CDH2	cadherin 2	-2.444286	-1.532433	-3.924959
NR_037195	LINC00607	long intergenic non-protein coding RNA 607	-3.321146	-1.607549	-3.926239
NM_001281429,NM_138371,XM_005269224,XM_011538978,XM_017020207,XM_017020208,XM_017020209,XM_017020210,XM_017020211,XM_017020212,XM_017020213,XM_017020214,XM_017020215,XM_017020216	PCED1B	PC-esterase domain containing 1B	-4.481908	-1.363878	-3.980387
NM_001304392,NM_002996	CX3CL1	C-X3-C motif chemokine ligand 1	-1.628152	-1.150311	-3.985463
NR_029193	SH3RF3-AS1	SH3RF3 antisense RNA 1	-2.480925	-1.802018	-4.019574

NM_001243270,NM_001243271,NM_014361,NM_175566,XM_011542871,XM_011542873,XM_017017926,XM_017017927,XM_017017928,XM_017017929,XR_001747909	CNTN5	contactin 5	-2.931601	-2.470344	-4.047595
NM_004210,XM_005270269,XM_005270270,XM_011540331,XM_011540332,XM_011540333,XM_011540335,XM_017016909,XR_945866	NEURL1	neuralized E3 ubiquitin protein ligase 1	-3.382243	-2.312809	-4.050622
NM_004750	CRLF1	cytokine receptor like factor 1	-4.200602	-2.186943	-4.063146
NM_020981,XM_005246931,XM_006712819,XM_011512085	B3GALT1	beta-1,3-galactosyltransferase 1	-2.367681	-1.489736	-4.067719
NM_000014,NM_001347423,NM_001347424,NM_001347425,XM_006719056	A2M	alpha-2-macroglobulin	-2.213664	-2.026817	-4.069645
XR_001743929,XR_926401,XR_926402	LOC100507336	uncharacterized LOC100507336, transcript variant X3	-2.195426	-1.635444	-4.126147
NM_021154,NM_058179	PSAT1	phosphoserine aminotransferase 1	-2.852238	-1.357228	-4.141147
NM_007021	DEPP1	DEPP1 autophagy regulator	-6.152267	-1.717979	-4.170142
NM_002237,XM_006723785,XM_011528800,XM_011528801,XM_011528802,XM_011528803,XM_011528804,XM_011528805,XM_011528806	KCNG1	potassium voltage-gated channel modifier subfamily G member 1	-3.407689	-2.002356	-4.192286

NM_000864	HTR1D	5-hydroxytryptamine receptor 1D	-4.148763	-1.536707	-4.220323
NM_001306129,NM_001306130,NM_001306131,NM_001306132,NM_001365517,NM_001365518,NM_001365519,NM_001365520,NM_001365521,NM_001365522,NM_001365523,NM_001365524,NM_002026,NM_054034,NM_212474,NM_212476,NM_212478,NM_212482,XM_005246397,XM_005246398,XM_005246399,XM_005246401,XM_005246402,XM_005246403,XM_005246404,XM_005246407,XM_005246408,XM_005246410,XM_005246411,XM_005246416,XM_017003692,XM_017003695,XM_024452769,XM_024452770	FN1	fibronectin 1	-2.655773	-1.545318	-4.225371
NM_000088,XM_005257058,XM_005257059,XM_011524341	COL1A1	collagen type I alpha 1 chain	-2.622211	-1.611723	-4.267514
NM_001346143,NM_001346144,NM_001346145,NM_001346146,NM_013231,NR_144385,NR_144386,NR_144387,XM_017021130,XM_017021131,XM_017021132,XM_017021133,XM_017021134,XM_024449522	FLRT2	fibronectin leucine rich transmembrane protein 2	-3.161786	-1.737765	-4.273099

NM_024512,XM_011534110,XM_017007177,XR_001740264	LRRC2	leucine rich repeat containing 2	-2.801629	-1.880069	-4.285833
NM_001301188,NM_001301190,NM_001301193,NM_001301196,NM_001301201,NM_021158,XM_017027989	TRIB3	tribbles pseudokinase 3	-3.097907	-1.990580	-4.295276
NM_018012	KIF26B	kinesin family member 26B	-2.165315	-2.025328	-4.321292
NM_001048209,NM_001144058,NM_001144059,NM_001352001,NM_001352002,NM_001352003,NM_001352004,NM_001352005,NM_001352006,NM_001352007,NM_001352008,NM_001352009,NM_016522,NR_147848,NR_147849,NR_147850,NR_147851,NR_147852,NR_147853,NR_147854,XM_011542861,XM_017017852,XM_017017854,XM_017017855,XM_017017856,XM_017017857,XM_017017858,XM_017017859,XM_017017860,XM_017017862,XM_017017863,XM_017017865,XM_017017866,XM_024448566,XM_024448567,XM_024448568,XM_024448569,XR_001747893,XR_001747894,XR_001747896	NTM	neurotrimin	-3.797632	-1.630673	-4.352314

NM_001128325,NM_001199021,NM_012445	SPON2	spondin 2	-3.068442	-1.782870	-4.395589
NM_020988,NM_138736,XM_011523003	GNAO1	G protein subunit alpha o1	-2.519569	-1.554533	-4.458324
NR_148200	L3MBTL2-AS1	L3MBTL2 antisense RNA 1	-3.173044	-1.538105	-4.460780
NM_001145204,NM_001145205,XM_005255539,XM_011522642,XR_001751976,XR_932915	SHISA9	shisa family member 9	-5.501778	-2.536695	-4.467956
NR_015423,NR_121191,NR_121192	PGM5-AS1	PGM5 antisense RNA 1	-2.825146	-1.421251	-4.488424
NM_000395,XM_005261340,XM_011529903,XM_011529904,XM_011529905	CSF2RB	colony stimulating factor 2 receptor subunit beta	-2.290997	-1.621936	-4.509955
NM_001244889,NM_170744,XM_011539453,XM_017015834,XM_017015835,XR_002956966,XR_945617	UNC5B	unc-5 netrin receptor B	-3.286228	-2.658119	-4.522378
NM_022358,XM_017028003	KCNK15	potassium two pore domain channel subfamily K member 15	-3.051814	-3.407084	-4.547854
NM_001130960,NM_001130961,NM_001349250,NM_001349251,NM_001349252,NM_014996,XM_005247238,XM_005247239,XM_011512560,XM_011512561,XM_011512562,XM_011512565,XM_011512566,XM_011512567,XM_017005923,XM_017005925,XM_01	PLCH1	phospholipase C eta 1	-2.366869	-2.167020	-4.562100

7005926,XM_017005927					
NM_001276282,NM_207303,NR_074088,XM_011539587,XM_011539588,XM_011539589,XM_011539590,XM_011539591,XM_017016035,XM_017016036,XM_017016037,XM_017016038,XM_017016039,XM_017016040,XR_001747080,XR_945653	ATRNL1	attractin like 1	-3.358114	-2.358709	-4.562466
NM_001937	DPT	dermatopontin	-3.786680	-1.875171	-4.626565
NM_001286617,NM_001286618,NM_001286619,NM_001286620,NM_001286622,NM_001286623,NM_001286624,NM_001286634,NM_001371369,NM_001371370,NM_001371371,NM_001371372,NM_001371373,NM_001371374,NM_001371376,NM_020152	MAP3K7CL	MAP3K7 C-terminal like	-2.496941	-1.604377	-4.684409
NM_001142393,NM_001271033,NM_006403,NM_182966,NR_073131	NEDD9	neural precursor cell expressed, developmentally down-regulated 9	-3.156786	-1.383491	-4.701168
NM_001204406,NM_001629,XM_017020522	ALOX5AP	arachidonate 5-lipoxygenase activating protein	-2.443126	1.149897	-4.708318
NM_001197295,NM_001197296,NM_001393,XM_017014376,XM_017014377,XM_02444	ECM2	extracellular matrix protein 2	-3.817274	-1.459600	-4.724331

7435,XM_024447436,XM_024447437					
NM_001382323,NM_001382324,NM_001382325,NM_001382326,NM_001382327,NM_001382328,NM_001382329,NM_001382330,NM_001382331,NM_001382332,NM_001382333,NM_001382334,NM_001382335,NM_001382336,NM_001382337,NM_001382338,NM_001382339,NM_001382340,NM_001382341,NR_168076,NR_168077,NR_168078,NR_168079,NR_168080,NR_168081,NR_168082,NR_168083,NR_168084	PKNOX2	PBX/knotted 1 homeobox 2	-3.398635	-1.764020	-4.744880
NR_038397,NR_103486	DNM3OS	DNM3 opposite strand/antisense RNA	-3.541484	-2.034472	-4.753434
NM_001163334,NM_001163335,NM_138780,XM_011544001,XM_011544002,XM_017029972,XM_017029973,XM_017029974	SYTL5	synaptotagmin like 5	-3.412566	-3.095563	-4.809786
NM_001318031,NM_020436,XM_005260467,XM_011528921,XM_011528922	SALL4	spalt like transcription factor 4	-3.783292	-2.631029	-4.829994
NM_000092,XM_005246281,XM_005246282,XM_006712246,XM_011510557,XM_011510558,XM_011510559,	COL4A4	collagen type IV alpha 4 chain	-3.856433	-1.140155	-4.869519

XM_011510560,XM_011510561,XM_011510562,XM_011510565,XM_011510566,XM_011510567,XM_011510568,XM_011510569,XM_011510570,XM_011510572,XM_017003296,XM_017003297,XM_017003298,XM_017003299,XM_017003300,XR_001738602,XR_001738603,XR_001738604,XR_001738606,XR_001738607,XR_922837					
NR_003190	USP32P1	ubiquitin specific peptidase 32 pseudogene 1	-2.668833	-1.742859	-4.879874
NM_206966,XM_005268446,XM_017009459	C5orf46	chromosome 5 open reading frame 46	-2.852998	-1.112002	-4.887686
NM_001105250,NM_001272020,NM_001330195,NM_001366425,NM_001366426,NM_004796,NM_138970,NR_073546,NR_073547,NR_158973,NR_158974,NR_158975,XM_005268218,XM_006720322,XM_011537363,XM_011537364,XM_011537365,XM_011537366,XM_011537367,XM_011537368,XM_011537369,XM_011537370,XM_011537371,XM_011537372,XM_011537373,XM_017021790,XM_017021791,XM_01	NRXN3	neurexin 3	-3.247936	-1.455793	-4.909823

7021792,XM_017021793,XM_017021794,XM_017021796,XM_017021797,XM_017021798,XM_017021799,XM_017021800,XM_017021801,XM_017021804,XM_017021805,XM_017021807,XM_024449750,XM_024449751,XM_024449752,XM_024449753,XR_001750599,XR_001750600,XR_001750602,XR_001750604,XR_001750605,XR_001750606,XR_001750607,XR_001750609,XR_001750610,XR_002957572,XR_943561,XR_943563					
NM_004770,XM_017013981,XM_017013982,XR_001745620,XR_001745621	KCNB2	potassium voltage-gated channel subfamily B member 2	-2.041702	-1.838087	-4.910827
NR_146076	USP32P3	ubiquitin specific peptidase 32 pseudogene 3	-2.271942	-1.625123	-4.958997
NR_160737	LOC107985184	uncharacterized LOC107985184	-2.644190	-1.863424	-4.978733
NM_001040260,NM_001040261,NR_036614,XM_005262785,XM_005262786,XM_017007832,XM_017007833,XM_024453913,XM_024453914,XM_024453915,XR_244621	DCLK2	doublecortin like kinase 2	-2.745932	-1.684134	-4.986170
NM_003486,XM_006721286,XM_017023735	SLC7A5	solute carrier family 7 member 5	-3.197739	-1.881086	-5.039085

,XM_017023736,XM_017023737					
NM_001199148,NM_001351537,NM_001351538,NM_001351539,NM_001351540,NM_173512,XM_006712337,XM_011510737,XM_017003455,XM_017003456,XM_017003457,XM_017003458,XM_017003459,XM_017003460,XM_017003461,XM_017003462,XM_017003463,XM_017003464,XM_017003465,XM_024452729,XR_001738648	SLC38A11	solute carrier family 38 member 11	-1.659637	-2.502689	-5.064429
NM_173593	B4GALNT3	beta-1,4-N-acetyl-galactosaminyltransferase 3	-2.647520	-2.310174	-5.080435
XR_001749246	LOC105369890	uncharacterized LOC105369890	-2.276915	-2.696469	-5.084815
NM_001003940,NM_001003942,NM_001003943,NM_033503	BMF	Bcl2 modifying factor	-6.354008	-3.409471	-5.182040
NM_001330163,NM_002308,NM_009587,NR_024043,XM_006721892,XM_006721893,XM_006721895,XM_011524796,XM_017024623	LGALS9	galectin 9	-2.596493	-1.750535	-5.196516
NM_001362435,NM_004932,XM_011513921,XM_017008910,XR_001741972	CDH6	cadherin 6	-3.463906	-2.722019	-5.314876

NM_001040708,NM_001282851,NM_012258	HEY1	hes related family bHLH transcription factor with YRPW motif 1	-4.843288	-1.741894	-5.357939
NM_001320643,XM_017028208	SIK1B	salt inducible kinase 1B (putative)	-2.582797	-2.394417	-5.393976
NM_001718	BMP6	bone morphogenetic protein 6	-3.401613	-1.877180	-5.458383
NM_021245	MYOZ1	myozenin 1	-3.576549	-1.702231	-5.496833
NM_173354,XM_011529474	SIK1	salt inducible kinase 1	-2.710993	-2.365768	-5.546555
NM_001099287,NM_001172292,XM_011534552,XM_024446043	NIPAL4	NIPA like domain containing 4	-6.432735	-1.666314	-5.557919
NM_152486	SAMD11	sterile alpha motif domain containing 11	-4.385482	-2.483708	-5.588999
NM_004936,NM_078487	CDKN2B	cyclin dependent kinase inhibitor 2B	-2.960539	-2.225886	-5.685711
NM_000908,NM_001204375,NM_001204376,NM_001363652,NM_001364458,NM_001364460,XM_011514047,XM_011514049,XM_011514050,XM_017009492	NPR3	natriuretic peptide receptor 3	-5.603135	-1.498800	-5.804379
NM_020815,NM_032961,XM_011532150	PCDH10	protocadherin 10	-3.382739	-2.033181	-5.811592
NM_001168214	C3orf80	chromosome 3 open reading frame 80	-3.676976	-3.328085	-5.832616
NM_183387,XM_006720070,XM_011536528,XM_011536530,XM_011536531,XM_011536532,XM_011536533,	EML5	EMAP like 5	-4.260527	-3.184302	-5.883002

XM_011536534,XM_011536536,XM_011536538,XM_017021062,XM_017021063,XM_017021064,XM_017021065,XM_017021066,XM_017021067,XM_017021068,XM_017021069,XM_017021070,XR_001750180,XR_001750181,XR_001750182					
NM_001160045,NM_001167609,NM_005269,XM_011538189,XM_011538190	GLI1	GLI family zinc finger 1	-3.081114	-2.226412	-5.888001
NM_001013698,XM_017019312	SMCO3	single-pass membrane protein with coiled-coil domains 3	-4.242319	-1.682745	-5.908672
NM_001001557	GDF6	growth differentiation factor 6	-3.902785	-1.857766	-6.001042
gene-RPL39P34	RPL39P34	ribosomal protein L39 pseudogene 34	-1.708081	-5.913885	-6.024085
NM_001546	ID4	inhibitor of DNA binding 4, HLH protein	-5.624073	-3.650166	-6.055771
NM_031866	FZD8	frizzled class receptor 8	-5.619738	-2.616641	-6.073280
NM_031935,XM_011510038,XM_011510041,XM_017002437,XM_024450118	HMCN1	hemicentin 1	-2.927670	-1.526911	-6.101532
NM_001282736,NM_032812,XM_011519750	PLXDC2	plexin domain containing 2	-3.247091	-2.304667	-6.329424
NM_139318,NM_172375	KCNH5	potassium voltage-gated channel subfamily H member 5	-5.334873	-2.670874	-6.407339
NM_002820,NM_198964,NM_198965,NM_1	PTHLH	parathyroid hormone like hormone	-3.989425	-1.936361	-6.559366

98966,XM_011520774,XM_017019675					
NM_001172771,NM_001172772,NM_152456	IL34	interleukin 34	-8.036322	-1.578406	-6.719064
NM_005159	ACTC1	actin alpha cardiac muscle 1	-5.110448	-3.846225	-6.721491
NM_014398,XM_005247360,XM_006713586,XM_011512688,XM_024453453	LAMP3	lysosomal associated membrane protein 3	-4.049702	-2.910421	-6.727481
NM_001143668,NM_001370299,NM_181847	AMIGO2	adhesion molecule with Ig like domain 2	-5.225009	-1.605766	-6.892786
NM_032229	SLITRK6	SLIT and NTRK like family member 6	-6.131887	-2.354928	-6.968488
XR_946886	LOC105378608	uncharacterized LOC105378608	-3.350970	-2.427939	-7.044153
XR_243788	LOC101927057	uncharacterized LOC101927057	-3.528464	-4.284490	-7.089946
NM_001243252,NM_001308395,NM_025202	EFHD1	EF-hand domain family member D1	-4.857218	-1.753065	-7.129560
NM_031479	INHBE	inhibin subunit beta E	-2.755476	-1.978017	-7.138855
NM_181606	KRTAP7-1	keratin associated protein 7-1	-2.955562	-1.629526	-7.302224
NR_038849	LINC01133	long intergenic non-protein coding RNA 1133	-6.368323	-1.904012	-7.460020
NR_168405	LOC107985728	collagen alpha-1(III) chain-like	-6.346545	-3.315748	-7.470303
XR_927844	LOC105375440	uncharacterized LOC105375440	-5.107654	-2.112349	-7.542760
NM_000739,NM_001006626,NM_001006627,NM_001006628,NM_	CHRM2	cholinergic receptor muscarinic 2	-3.197621	-1.723142	-7.549883

001006629,NM_001006630,NM_001006631,NM_001006632,NM_001378972,NM_001378973					
NM_002281	KRT81	keratin 81	-5.931500	-5.784226	-7.882391
NM_153225,XM_017013145,XM_024447081,XR_928762	SBSPON	somatomedin B and thrombospondin type 1 domain containing	-3.283928	-2.682977	-7.971904
NR_002312	RPPH1	ribonuclease P RNA component H1	-2.855470	-5.317285	-8.205599
NM_005447,XM_011538988	RASSF9	Ras association domain family member 9	-5.180605	-2.235549	-8.338845
NM_138433	KLHDC7B	kelch domain containing 7B	-7.770664	-2.170386	-8.370759
XR_001753890	LOC107985336	uncharacterized LOC107985336	-6.759637	-2.619647	-8.578320
NM_139056,NR_136935,XR_001742032	ADAMTS16	ADAM metallopeptidase with thrombospondin type 1 motif 16	-4.240678	-2.939798	-8.654543
NM_001321015,NM_001482	GATM	glycine amidinotransferase	-8.710381	-2.224459	-8.680656
NM_017594	DIRAS2	DIRAS family GTPase 2	-5.737861	-4.628943	-8.794477
XR_001737736	LINC02806	long intergenic non-protein coding RNA 2806	-4.139753	-2.805864	-9.299797
NM_002192,XM_017012174,XM_017012175,XM_017012176	INHBA	inhibin subunit beta A	-4.842903	-2.456224	-9.374595
NM_005630,XM_017007077,XM_024453721	SLCO2A1	solute carrier organic anion transporter family member 2A1	-5.319936	-3.900851	-9.604294

NM_001329996,NM_001329997,NM_175887,XM_011515198,XM_011515199	PRR15	proline rich 15	-4.250182	-2.523013	-9.702827
NM_001143836,NM_001143837,NM_001291926,NM_001291927,NM_001291929,NM_001300995,NM_016931,NR_120406,XM_006718849,XM_011542857,XM_017017841,XM_017017842,XM_017017843,XM_017017844,XM_017017845	NOX4	NADPH oxidase 4	-5.581603	-2.439817	-9.775537
NM_030761,XM_011541597,XM_011541598,XM_011541599	WNT4	Wnt family member 4	-4.599853	-3.076531	-9.980521
NR_104185,NR_160710,NR_160711	CCDC144NL-AS1	CCDC144NL antisense RNA 1	-9.648636	-1.817738	-10.260275
NM_001367348	SMIM34A	small integral membrane protein 34A	-5.681320	-2.466431	-10.717004
NM_021094,NM_134431,XM_005253474,XM_005253477,XM_011520818,XM_011520819,XM_011520820,XM_011520821,XM_017019849,XM_017019850,XM_024449138,XM_024449139	SLCO1A2	solute carrier organic anion transporter family member 1A2	-6.106537	-3.480869	-11.175729
NR_110856	LOC101928516	uncharacterized LOC101928516	-5.656394	-2.142370	-11.341262
XR_001755792,XR_001755793	LOC107985715	uncharacterized LOC107985715, transcript variant X1	-4.024091	-1.066989	-11.471452
XR_001755794	LOC107985716	uncharacterized LOC107985716	-5.045702	-1.067890	-12.202239

NM_001278733,NM_054114,NM_138810,NM_152133	TAGAP	T cell activation RhoGTPase activating protein	-8.042906	-2.440374	-12.783461
NM_020318,NM_021936,XM_005245422,XM_011509857,XM_011509858,XM_017002023,XM_017002024,XM_017002025,XR_921907	PAPPA2	pappalysin 2	-4.359471	-1.924192	-12.967866
NM_001282941,NM_145307,XM_011539456,XM_011539457,XM_011539460,XM_017015843,XM_017015844,XR_001747053,XR_945618	RTKN2	rhotekin 2	-6.173104	-3.411356	-13.495186
NM_001193335,NM_017680	ASPN	asporin	-5.269859	-2.417423	-14.141296
NM_001855,XM_011518214	COL15A1	collagen type XV alpha 1 chain	-7.631679	-2.873346	-14.289086
NM_005221,XM_005250185,XM_017011803	DLX5	distal-less homeobox 5	-4.638593	-2.555993	-14.413363
NM_000231,NM_001378244,NM_001378245,NM_001378246	SGCG	sarcoglycan gamma	-7.628914	-2.447756	-17.924512
NM_005092	TNFSF18	TNF superfamily member 18	-4.840572	-2.992276	-18.251774
NM_000621,NM_001165947,NM_001378924	HTR2A	5-hydroxytryptamine receptor 2A	-7.697158	-1.913385	-18.666164
NM_005838,NM_201648,XM_017017087	GLYAT	glycine-N-acyltransferase	-8.031532	-2.917010	-19.121216
NM_002238,NM_172362,XM_017001246	KCNH1	potassium voltage-gated channel subfamily H member 1	-8.292527	-2.768047	-24.613536

NM_000399,NM_001136177,NM_001136178,NM_001136179,NM_001321037,XM_011539427	EGR2	early growth response 2	-8.450648	-2.560781	-25.835043
NM_000900,NM_001190839	MGP	matrix Gla protein	-8.780587	-3.425091	-26.748629
NM_000501,NM_001081752,NM_001081753,NM_001081754,NM_001081755,NM_001278912,NM_001278913,NM_001278914,NM_001278915,NM_001278916,NM_001278917,NM_001278918,NM_001278939,XM_005250187,XM_005250188,XM_011515868,XM_011515869,XM_011515870,XM_011515871,XM_011515872,XM_011515873,XM_011515874,XM_011515875,XM_011515876,XM_011515877,XM_017011813,XM_017011814	ELN	elastin	-21.589130	-2.321319	-27.104905
NM_000095	COMP	cartilage oligomeric matrix protein	-8.340907	-5.206235	-34.498990

Table S2. List of 398 genes that were not significantly changed by SB590885 or SB203580 but were significantly changed by co-treatment with SB590885 and SB203580.

Transcript_ID	Gene_Symbol	Description	Relative expression (SB590885 and SB203580//DMSO)
NM_002986	CCL11	C-C motif chemokine ligand 11	4.531963
XR_001741442	LOC107986196	uncharacterized LOC107986196	4.525134
NR_109971,NR_109972	LINC01483	long intergenic non-protein coding RNA 1483	3.982501
NM_033059	KRTAP4-11	keratin associated protein 4-11	3.920655
NM_001330065,NM_001369869,XM_006723946	KCNE1B	potassium voltage-gated channel subfamily E regulatory subunit 1B	3.864080
NR_026998	LOC91450	uncharacterized LOC91450	3.781688
gene-LOC401218	LOC401218	cyclin Q pseudogene	3.678977
NM_001193532,NM_152304,XM_017000226	RAB42	RAB42, member RAS oncogene family	3.660150
gene-CYCSP34	CYCSP34	CYCS pseudogene 34	3.115948
NM_022120	OXCT2	3-oxoacid CoA-transferase 2	3.104758
NM_001184779,NM_001184780,NM_024505,NR_033671,NR_033672	NOX5	NADPH oxidase 5	3.103827
NM_000221,NM_006488,XM_005264294,XM_005264296,XM_005264298,XM_006712008,XM_006712009,XM_006712010,XM_006712011,XM_006712012,XM_006712013,XM_006712014,XM_017004060,XM_017004061	KHK	ketohexokinase	3.074477
NR_015384	LOC100126784	uncharacterized LOC100126784	2.964672
NM_001370351,NM_001370352,NM_001370353,NM_001370354,NM_006467,XM_011543101,XM_011543102,XM_011543105,XM_017008957	POLR3G	RNA polymerase III subunit G	2.962448
NM_001346880,XM_017004063,XM_017004064,XM_017004065,XM_017004066,XM_017004067,XM_0	MFSD2B	major facilitator superfamily domain containing 2B	2.917080

17004068,XM_017004069,XM_017004070,XR_001738742			
NM_000956	PTGER2	prostaglandin E receptor 2	2.917041
NR_136175	LINC01960	long intergenic non-protein coding RNA 1960	2.912063
NM_001161575,NM_018296,XM_005256025,XM_005256026,XM_005256027,XM_005256028,XM_011523200,XM_011523201,XM_011523202,XM_011523203,XM_011523204,XM_011523205,XM_011523206,XM_017023400,XM_017023401,XM_017023402,XM_024450338,XR_243416,XR_429723	LRRC36	leucine rich repeat containing 36	2.839515
NM_001248,NM_001291960,NM_001291961,XM_011534266	ENTPD3	ectonucleoside triphosphate diphosphohydrolase 3	2.834599
NM_001287489,NM_004802,NM_194248,NM_194322,NM_194323	OTOF	otoferlin	2.816913
NM_018476	BEX1	brain expressed X-linked 1	2.774684
NM_001199771,NM_002905	RDH5	retinol dehydrogenase 5	2.756407
NR_033995	LOC389247	uncharacterized LOC389247	2.732412
NR_038285	MELTF-AS1	MELTF antisense RNA 1	2.700558
NM_001319077,NM_001319078,NM_001319079,NM_001319080,NM_001319081,NM_001319082,NM_002131,NM_145899,NM_145901,NM_145902,NM_145903,NM_145905	HMGA1	high mobility group AT-hook 1	2.665987
NM_002178	IGFBP6	insulin like growth factor binding protein 6	2.645364
NM_031960	KRTAP4-8	keratin associated protein 4-8	2.645034
NM_001066,XM_011542060,XM_011542063,XM_017002211,XM_017002214,XM_017002215	TNFRSF1B	TNF receptor superfamily member 1B	2.638649
XR_244188	LOC101927486	uncharacterized LOC101927486	2.632523

NM_032413,NM_197955	C15orf48	chromosome 15 open reading frame 48	2.605077
gene-HMGA1P3	HMGA1P3	high mobility group AT-hook 1 pseudogene 3	2.600648
NM_001080457,NM_001348568,XM_005259429,XM_006723506,XM_011527520	LRRC4B	leucine rich repeat containing 4B	2.590372
NM_000663,NM_001127448,NM_020686,XM_011522400,XM_011522401	ABAT	4-aminobutyrate aminotransferase	2.588352
NM_001009954,NM_017669	ERCC6L	ERCC excision repair 6 like, spindle assembly checkpoint helicase	2.585356
NM_001376887,NM_003807,NM_172014,XM_017027418,XR_001753777,XR_936212	TNFSF14	TNF superfamily member 14	2.574964
XR_950269	LOC105369370	uncharacterized LOC105369370	2.551970
NM_001100620,NM_001278324,NM_005480,XM_006719181,XM_011537723,XM_011537724,XR_944445,XR_944446	TROAP	trophinin associated protein	2.527258
XR_001738225,XR_922117	LOC105371427	uncharacterized LOC105371427, transcript variant X2	2.523977
NM_001310220,NM_001501,NM_178331,NM_178332,XM_017027823	GNRH2	gonadotropin releasing hormone 2	2.504201
gene-MT2P1	MT2P1	metallothionein 2 pseudogene 1	2.498544
NM_000757,NM_172210,NM_172211,NM_172212,XM_017000369	CSF1	colony stimulating factor 1	2.495574
NM_001363555,NM_175617	MT1E	metallothionein 1E	2.494610
NM_001220494,NM_001220496,NM_001354699,NM_080661,XM_011545354,XM_011545355,XM_011545356,XM_011545357,XM_011545358,XM_011545359,XM_024448769	GLYATL1	glycine-N-acyltransferase like 1	2.493069
NM_144683	DHRS13	dehydrogenase/reductase 13	2.489621
NR_026964	DKFZP434A062	uncharacterized LOC26102	2.475646

NM_001258450,NM_001258451,NM_004203,NM_182687,XM_011522734,XM_011522735,XM_011522736,XM_024450490	PKMYT1	protein kinase, membrane associated tyrosine/threonine 1	2.463895
NM_001095,NM_001256830,NM_020039,NR_046389,XM_011538350,XM_011538351,XM_011538352	ASIC1	acid sensing ion channel subunit 1	2.462221
NM_006829	ADIRF	adipogenesis regulatory factor	2.461520
NR_103553,NR_103554	LIFR-AS1	LIFR antisense RNA 1	2.458911
NM_004878	PTGES	prostaglandin E synthase	2.452794
NM_001276451,XM_011540089	DRGX	dorsal root ganglia homeobox	2.449093
NM_001302123,NM_005409	CXCL11	C-X-C motif chemokine ligand 11	2.446400
NM_000872,NM_019859,NM_019860,XM_024447973	HTR7	5-hydroxytryptamine receptor 7	2.443911
NM_001160130,NM_001160132,NM_001160133,NM_001160134,NM_019842,XM_017011058,XM_024446492,XM_024446493	KCNQ5	potassium voltage-gated channel subfamily Q member 5	2.441495
NM_002531,XM_011528827	NTSR1	neurotensin receptor 1	2.439811
NM_001301161,NM_152750,XM_005250224,XM_005250225,XM_006715905,XM_011515955,XM_011515956,XM_011515957,XM_011515958,XM_011515959,XM_017011862,XM_017011863,XM_017011864,XM_017011865,XM_017011866,XM_017011867,XM_024446689,XM_024446690,XR_001744598,XR_001744600,XR_001744601,XR_001744602,XR_001744603,XR_001744604,XR_001744605,XR_001744606,XR_001744607,XR_927414	CDHR3	cadherin related family member 3	2.434751
NM_001256834,NM_001284526,NM_001313950,NM_001313951,NM_001313952,NM_001313953,NM_001313954,NM_001313955,NM_004217,NR_132730,NR_132731,XM_0	AURKB	aurora kinase B	2.432148

11524072,XM_017025307,XM_017025308,XM_017025309,XM_017025310,XM_017025311			
NM_001281987,NM_001281988,NM_144569,XM_011542413,XM_011542414,XM_017002779,XM_017002780,XM_017002781,XM_017002782,XR_001737533,XR_946797	SPOCD1	SPOC domain containing 1	2.422645
NR_073447	LINC00595	long intergenic non-protein coding RNA 595	2.409576
NM_000015,XM_017012938	NAT2	N-acetyltransferase 2	2.409389
NM_030928	CDT1	chromatin licensing and DNA replication factor 1	2.407940
NM_002090	CXCL3	C-X-C motif chemokine ligand 3	2.406418
NM_001098500,NM_001282767,NM_001282768,NM_001282769,NM_001282770,NM_001321681,NM_019590,XM_005252516,XM_011519552,XM_011519555,XM_011519558,XM_011519559,XM_011519562,XM_011519564,XM_011519565,XM_011519566,XM_017016416,XM_017016417,XM_017016418,XM_017016419,XM_017016420,XM_017016421,XM_017016422,XM_017016423,XM_017016424,XM_017016425,XM_017016426,XM_017016427,XM_017016429,XM_024448082,XM_024448083,XM_024448084,XM_024448085,XM_024448086	KIAA1217	KIAA1217	2.405469
NM_024734,XM_011537158,XM_011537159,XM_017021646,XM_017021647,XR_001750558,XR_245721,XR_429330,XR_429332	CLMN	calmin	2.392802
NR_120519,NR_120520	MUC12-AS1	MUC12 antisense RNA 1	2.385981
NM_001323517,NM_001323518,NM_006744	RBP4	retinol binding protein 4	2.381851

NM_001243773,NM_001243774,NM_053064,XM_006720173,XM_011536846,XM_017021376,XM_017021377,XM_024449629,XM_024449630,XM_024449631,XM_024449632,XM_024449633,XM_024449634	GNG2	G protein subunit gamma 2	2.368045
NM_173564	NYAP1	neuronal tyrosine phosphorylated phosphoinositide-3-kinase adaptor 1	2.366615
NR_125997	RNF207-AS1	RNF207 antisense RNA 1	2.363118
NM_015123,XM_005264722,XM_005264723,XM_017005989,XM_017005990,XM_017005991,XM_017005992,XM_017005993,XM_017005994,XM_017005995,XM_017005996,XM_017005997	FRMD4B	FERM domain containing 4B	2.354960
NM_001143783,NM_001143784,NM_001143785,NM_002005,XM_005254880,XM_005254882,XM_017022005,XM_017022006,XM_017022007,XM_017022008,XM_017022009,XM_017022010,XR_001751142,XR_001751143,XR_001751144,XR_243206	FES	FES proto-oncogene, tyrosine kinase	2.348494
NM_018154	ASF1B	anti-silencing function 1B histone chaperone	2.345096
NM_005879,XM_011533264,XM_017005526,XR_001739979	TRAIP	TRAF interacting protein	2.340623
gene-PEBP1P2	PEBP1P2	phosphatidylethanolamine binding protein 1 pseudogene 2	2.327786
NM_005923,XM_011535839,XM_017010870,XM_017010871,XM_017010872,XM_017010873,XM_017010874,XM_017010875,XM_017010876,XM_017010877	MAP3K5	mitogen-activated protein kinase kinase kinase 5	2.301082
NM_001281710,NM_001281711,NM_001281712,NM_015341,XM_005263908,XM_006712388	NCAPH	non-SMC condensin I complex subunit H	2.299493
NM_001281768,NM_021116,XM_005249584,XM_005249585	ADCY1	adenylate cyclase 1	2.291831

NM_001024844,NM_002231,XM_006718223,XM_011520067	CD82	CD82 molecule	2.287552
NM_001254,XM_011525541,XM_011525542	CDC6	cell division cycle 6	2.284447
NM_001039844	ACBD7	acyl-CoA binding domain containing 7	2.283444
NM_001010898,XM_006710643	SLC6A17	solute carrier family 6 member 17	2.280864
NM_006342,XM_005247929,XM_005247930,XM_011513386,XM_017007653	TACC3	transforming acidic coiled-coil containing protein 3	2.280853
NM_000591,NM_001040021,NM_001174104,NM_001174105	CD14	CD14 molecule	2.270460
NR_135595	ZNF687-AS1	ZNF687 antisense RNA 1	2.268943
NM_005030	PLK1	polo like kinase 1	2.264322
NM_001144996,NM_001144997,NM_001367993,NM_001367994,NM_001374465,NM_002206,XM_005268839,XM_005268840,XM_005268841,XM_005268844,XM_005268846,XM_005268848,XM_005268849,XM_005268850,XM_017019265	ITGA7	integrin subunit alpha 7	2.259942
NR_146531	LNCOG	lncRNA osteogenesis associated	2.256732
NM_000899,NM_003994	KITLG	KIT ligand	2.254082
NM_002994	CXCL5	C-X-C motif chemokine ligand 5	2.253701
NR_102735	DBH-AS1	DBH antisense RNA 1	2.252213
NM_001197330,NM_001197331,NM_001348384,NM_001348385,NM_002692,XM_011536842,XM_011536843,XM_011536844,XM_017021374,XR_001750377,XR_001750378,XR_943479,XR_943480	POLE2	DNA polymerase epsilon 2, accessory subunit	2.251175
NM_001083961,NM_173636,XM_005258809,XM_011526837,XM_011526838,XM_011526839,XM_011526840,XM_011526841,XM_011526842,XM_011526843,XM_011526844	WDR62	WD repeat domain 62	2.248970

,XM_017026665,XR_001753671,XR_001753672			
XR_001746881,XR_001746882	LOC105376214	uncharacterized LOC105376214, transcript variant X1	2.241885
NM_012291,XM_006719705,XM_011539024,XM_011539025,XM_017020253,XR_001748927	ESPL1	extra spindle pole bodies like 1, separase	2.240088
gene-LOC728688	LOC728688	ubiquitin like with PHD and ring finger domains 1 pseudogene	2.235900
NM_016095	GINS2	GINS complex subunit 2	2.232507
NM_001039775,XM_005245918,XM_011541672,XM_011541673,XR_001737260,XR_946681	CRYBG2	crystallin beta-gamma domain containing 2	2.231241
XR_936966	LOC105372708	uncharacterized LOC105372708	2.228273
NM_001190456,NM_001190457,NM_001324014,NM_001324015,NM_006091	CORO2B	coronin 2B	2.226911
NM_002729	HHEX	hematopoietically expressed homeobox	2.223561
NM_024081,XM_006718313,XM_006718314	PRRG4	proline rich and Gla domain 4	2.214924
NM_002275,XM_011524784,XM_017024614	KRT15	keratin 15	2.203564
NM_018159	NUDT11	nudix hydrolase 11	2.198564
NM_001258357,NM_001258358,NM_001258359,NM_002149,NM_134421,XM_005246160,XM_005246161,XM_005246162,XM_005246163,XM_011510346,XM_011510347,XM_011510348,XM_011510349,XM_017003950,XM_017003951,XM_017003952,XM_017003953	HPCAL1	hippocalcin like 1	2.190063
NM_080668,XM_005273733,XM_005273734,XM_011544743,XM_011544747,XM_011544748,XM_011544749,XM_017017158,XR_001747757,XR_001747758	CDCA5	cell division cycle associated 5	2.189944

NM_002263,XM_011514585,XM_011514587,XM_017010836,XM_017010837	KIFC1	kinesin family member C1	2.186915
NM_018518,NM_182751,XM_011519538	MCM10	minichromosome maintenance 10 replication initiation factor	2.186072
NR_103778,NR_103779	FLG-AS1	FLG antisense RNA 1	2.178321
NM_001301339,NM_213720,NR_125755,NR_125756	CHCHD10	coiled-coil-helix-coiled-coil-helix domain containing 10	2.176467
NM_003206,NM_198392	TCF21	transcription factor 21	2.176190
NM_001142935,NM_031300	MXD3	MAX dimerization protein 3	2.176100
NM_001007255,NM_152366,NR_033385,NR_033386	KLHDC9	kelch domain containing 9	2.173330
NR_145451	LINC02012	long intergenic non-protein coding RNA 2012	2.168633
NM_001031683,NM_001289758,NM_001289759,NM_001549	IFIT3	interferon induced protein with tetratricopeptide repeats 3	2.162224
NR_026934	LINC02085	long intergenic non-protein coding RNA 2085	2.160982
NM_178171,XM_006721832,XM_011524651,XM_017024502,XM_017024503,XM_017024504	GSDMA	gasdermin A	2.157765
NM_000418,NM_001257406,NM_001257407,NM_001257997,XM_005255308,XM_006721043,XM_011545825,XM_011545826,XM_011545827,XM_011545828,XM_011545829,XM_011545830,XM_011545831,XM_011545832,XM_011545833,XM_011545834,XM_017023211	IL4R	interleukin 4 receptor	2.153559
NM_033184	KRTAP2-4	keratin associated protein 2-4	2.150703
NM_001185056,NM_005602	CLDN11	claudin 11	2.149102
NM_019886	CHST7	carbohydrate sulfotransferase 7	2.148838
NM_001170806,NM_173549	ERICH5	glutamate rich 5	2.147329
NM_001085480	FAM162B	family with sequence similarity 162 member B	2.138333

NM_002596,NM_212502,NM_212503,XM_011509602,XM_017001423,XR_001737212,XR_001737213,XR_002956776,XR_426779,XR_426780,XR_426781,XR_426782,XR_426784,XR_921822	CDK18	cyclin dependent kinase 18	2.136613
NM_001267580,NM_003981,NM_199413,XM_005254987,XM_006720759,XM_006720760,XM_011522187,XM_011522188,XM_011522189,XM_011522190,XM_011522191,XM_011522192,XM_017022712,XM_017022713,XM_017022714,XM_017022715,XM_017022716,XM_017022717	PRC1	protein regulator of cytokinesis 1	2.135830
NM_001278616,NM_001278617,NM_004336,XR_923001	BUB1	BUB1 mitotic checkpoint serine/threonine kinase	2.134614
NM_001351678,NM_152476,XM_011527696,XM_011527697,XM_017026327,XM_017026328,XM_017026329,XM_017026330,XM_024451380	ZNF560	zinc finger protein 560	2.133864
NR_026815	TMEM191A	transmembrane protein 191A (pseudogene)	2.129681
NM_052969	RPL39L	ribosomal protein L39 like	2.120800
XR_940324	LOC105374848	uncharacterized LOC105374848	2.117256
NM_001367886,NM_207311,NR_147892,NR_147893,NR_147894,XM_006719694,XM_006719696,XM_006719697,XM_011538999,XM_011539000,XM_011539001,XR_001748923,XR_944834,XR_944836	BICDL1	BICD family like cargo adaptor 1	2.112491
NM_001185015,NM_001378442,NM_001378443,NM_001378444,NM_001378445,NM_001378446,NM_001378447,NM_004509,NM_004510,NM_080424,XM_011511090,XM_011511091,XM_011511092,XM_01	SP110	SP110 nuclear body protein	2.106807

7003968,XM_017003969,XM_024452850			
NM_001039656,NM_004923,XM_011545402,XM_011545403,XM_017018588,XR_950115	TESMIN	testis expressed metallothionein like protein	2.105589
NR_037853	ATP6V1G2-DDX39B	ATP6V1G2-DDX39B readthrough (NMD candidate)	2.104907
NM_016426,XM_005261627,XM_011530213,XM_017028816	GTSE1	G2 and S-phase expressed 1	2.103362
NM_001199864	BCL2L2-PABPN1	BCL2L2-PABPN1 readthrough	2.102867
NM_001330430,NM_020485,NM_138616,NM_138617,NM_138618,XM_005245957,XM_006710810,XM_011541888,XM_011541889,XM_011541891,XM_017002014	RHCE	Rh blood group CcEe antigens	2.101558
NM_006668,XM_005267274,XM_011536364,XM_011536365,XM_017020933	CYP46A1	cytochrome P450 family 46 subfamily A member 1	2.099606
NM_001297602,NM_001297603,NM_001297604,NM_001331019,NM_031299,NR_123727,NR_138531,XR_001748879,XR_001748880,XR_001748881,XR_002957383,XR_002957384,XR_002957385	CDCA3	cell division cycle associated 3	2.097527
NM_001166260,NM_004237,XM_011514163	TRIP13	thyroid hormone receptor interactor 13	2.093871
NM_015714	G0S2	G0/G1 switch 2	2.089138
NM_001289067,NM_001289068,NM_001289069,NM_001289070,NM_001289071,NM_001289072,NM_001289073,NM_001289074,NM_001289075,NM_018063,XM_024447967,XM_024447968	HELLS	helicase, lymphoid specific	2.088563
NM_001282962,NM_001282963,NM_018410,XM_011511437	HJURP	Holliday junction recognition protein	2.088191
NM_001174159,NM_001174160,NM_001363110,NM_001363111,NM_022071,NR_156444	SH2D4A	SH2 domain containing 4A	2.087383

NM_153221	CILP2	cartilage intermediate layer protein 2	2.085369
NM_004482,XM_005246449,XM_011510929,XM_017003770,XR_002959253	GALNT3	polypeptide N-acetylgalactosaminyltransferase 3	2.085279
NM_001145664,NM_001367508,NM_001367509,NM_001367510,XM_011511771,XM_011511772,XM_011511773,XM_011511776,XM_011511777,XM_011511778,XM_017004851,XM_017004852,XM_017004853,XM_017004854,XR_001738924	RFX8	RFX family member 8, lacking RFX DNA binding domain	2.082836
NR_022014	HMGN2P46	high mobility group nucleosomal binding domain 2 pseudogene 46	2.082354
NM_001243088,NM_001243089,NM_021953,NM_202002,NM_202003,XM_005253676,XM_011520930,XM_011520931,XM_011520932,XM_011520933,XM_011520934,XM_011520935,XR_931507	FOXM1	forkhead box M1	2.081843
NM_001166131,NM_152463,XM_005257081,XM_011524395,XM_017024236,XR_002957972,XR_934390	EME1	essential meiotic structure-specific endonuclease 1	2.081393
NM_005225	E2F1	E2F transcription factor 1	2.078167
NM_005929,NM_033316,XM_006713643,XM_011512850,XM_011512851	MELTF	melanotransferrin	2.077697
NM_000775,NR_134981,NR_134982	CYP2J2	cytochrome P450 family 2 subfamily J member 2	2.077546
NM_001040059,NM_001251	CD68	CD68 molecule	2.072476
NR_040288,NR_040289	AKR7L	aldo-keto reductase family 7 like (gene/pseudogene)	2.071226
NM_001009991,NM_001242384,NM_001242394,NM_001242395,NM_001318745,XM_005267215,XM_005267218,XM_005267222,XM_006715605,XM_006715606,XM_006715611,XM_011536254,XM_011536255,XM_017011495,XM_017011496	SYTL3	synaptotagmin like 3	2.067906

,XM_017011497,XM_017011498,XM_017011499,XM_024446588			
NM_001005413,NM_007057,NM_032997,XM_017015605,XM_024447784,XR_428692	ZWINT	ZW10 interacting kinetochore protein	2.066330
NM_001257135,NM_002309,XM_024452239,XM_024452240	LIF	LIF interleukin 6 family cytokine	2.062617
NM_001547	IFIT2	interferon induced protein with tetratricopeptide repeats 2	2.052038
NM_000161,NM_001024024,NM_001024070,NM_001024071,XM_017021218	GCH1	GTP cyclohydrolase 1	2.050043
XR_001741915,XR_001741916	LOC107986325	uncharacterized LOC107986325, transcript variant X2	2.048630
NM_005953	MT2A	metallothionein 2A	2.048043
NM_001031739,NM_001168530,NM_001168531,NM_024087,XM_005274446,XM_011545458,XM_011545460,XM_017029283,XM_017029284,XM_017029285,XM_017029286,XM_024452341	ASB9	ankyrin repeat and SOCS box containing 9	2.046694
NM_002970,NR_027783,XM_024452421	SAT1	spermidine/spermine N1-acetyltransferase 1	2.046693
NM_001193333,NM_007074,XM_011545714,XM_017022885,XM_017022886	CORO1A	coronin 1A	2.044508
NM_001282062,NM_001282063,NM_001282068,NM_003586,NR_104089,NR_104090,XM_011545975,XM_017023778,XM_024450472,XM_024450473,XM_024450474	DOC2A	double C2 domain alpha	2.043519
NM_001103160,NM_001103161,XM_011541459,XM_011541460,XM_011541461,XM_011541462	SH2D5	SH2 domain containing 5	2.042146
NM_001100118,NM_001100119,NM_001371229,NM_001371231,NM_001371232,NM_005432,XM_005268046	XRCC3	X-ray repair cross complementing 3	2.038449
NM_000062,NM_001032295	SERPING1	serpin family G member 1	2.036370

NM_138444	KCTD12	potassium channel tetramerization domain containing 12	2.028948
NM_001171251,NM_001321310,NM_001321311,NM_024032,XM_011525187,XM_011525188,XM_011525189,XM_011525190,XM_011525191,XM_011525192,XM_011525193,XM_011525194,XM_011525195,XM_011525197,XR_002958064,XR_934545	HROB	homologous recombination factor with OB-fold	2.026023
NM_006397	RNASEH2A	ribonuclease H2 subunit A	2.024541
NM_153695,XM_011518345	ZNF367	zinc finger protein 367	2.018315
NM_001005376,NM_001005377,NM_001301037,NM_002659,XM_005258990,XM_011527027,XM_011527028,XM_011527029,XM_011527030,XM_011527031,XM_017026872,XM_017026873	PLAUR	plasminogen activator, urokinase receptor	2.017511
NM_001165,NM_182962,XM_024448467	BIRC3	baculoviral IAP repeat containing 3	2.016600
NM_000602	SERPINE1	serpin family E member 1	2.015916
NM_001145775,NM_001145776,NM_001145777,NM_004117	FKBP5	FKBP prolyl isomerase 5	2.015729
NM_012306,XM_005268730	FAIM2	Fas apoptotic inhibitory molecule 2	2.014370
NM_020439,XM_017001866,XM_017001867	CAMK1G	calcium/calmodulin dependent protein kinase IG	2.013426
NM_001136023,NM_001261461,NM_006163	NFE2	nuclear factor, erythroid 2	2.012763
NM_001003827,NM_021616,NM_130390	TRIM34	tripartite motif containing 34	2.012148
NM_004051,NM_203314,NM_203315,XM_005269352,XM_005269355,XM_011513067,XM_017007007,XM_017007008,XM_017007009,XM_017007010,XM_017007011,XM_017007012,XM_017007013,XM_017007015,XR_001740229	BDH1	3-hydroxybutyrate dehydrogenase 1	2.010148

NM_001130688,NM_001130689,NM_002129	HMGB2	high mobility group box 2	2.009534
NM_001284511,NM_001284512,NM_001284513,NM_001324118,NM_152341	PAQR4	progestin and adipoQ receptor family member 4	2.007528
NM_005167	PPM1J	protein phosphatase, Mg2+/Mn2+ dependent 1J	2.004187
NM_000405,NM_001167607	GM2A	GM2 ganglioside activator	2.003848
NM_001355404,NM_001355405,NM_001355406,XM_024451520,XM_024451521	ZNF724	zinc finger protein 724	2.003396
NM_152446,NR_157142,XM_011536490,XM_011536491,XM_011536492,XM_011536493,XM_011536495,XM_017021042,XM_017021043	CEP128	centrosomal protein 128	2.003317
NM_001017523,NM_001018072,NM_001347943,NM_001347944,XM_005268645,XM_011537909,XM_011537910,XM_017018807	BTBD11	BTB domain containing 11	2.002882
NM_001319224,NM_003686,NM_006027,NM_130398,XM_006711840,XM_011544321,XM_011544322,XM_011544323,XM_011544324,XM_011544325,XM_017002793	EXO1	exonuclease 1	2.002307
NR_131982	LOC105373383	uncharacterized LOC105373383	2.001303
NM_001128850,NM_004165	RRAD	RRAD, Ras related glycolysis inhibitor and calcium channel regulator	2.000762
NM_001165252	KRTAP2-3	keratin associated protein 2-3	2.000589
NR_015421	LOC154761	family with sequence similarity 115, member C pseudogene	2.000473
NR_144550	SDAD1P1	SDA1 domain containing 1 pseudogene 1	-2.002095
NM_016224,XM_005267015,XM_011535886	SNX9	sorting nexin 9	-2.003129
NM_003966,XM_006714506,XM_006714507,XM_011514155,XM_011514156,XM_011514157,XM_01151	SEMA5A	semaphorin 5A	-2.006606

4158,XM_011514159,XM_017010016			
XR_001742582,XR_001742583,XR_001742584,XR_001742585,XR_001742586,XR_001742587,XR_002956209,XR_241729,XR_925722,XR_925725	LOC101929200	uncharacterized LOC101929200, transcript variant X3	-2.007709
NM_001168399,NM_001168400,NM_001168401,NM_032621	BEX2	brain expressed X-linked 2	-2.008317
NM_144717,XM_006713665,XM_011512910,XM_011512911,XM_017006632	IL20RB	interleukin 20 receptor subunit beta	-2.008548
NM_001290360,NM_001324101,NM_001324103,NM_001324106,NM_001324107,NM_001324108,NM_001324109,NM_001324111,NM_001364155,NM_001364156,NM_001364157,NM_001364158,NM_001364159,NM_024007,NM_182708,XM_017009192,XM_017009193,XM_017009194,XM_017009195,XM_017009196,XM_017009197,XM_017009199,XM_017009200,XM_017009201,XM_017009202,XM_017009203,XM_017009204,XM_024454390,XM_024454391,XM_024454392,XM_024454393	EBF1	EBF transcription factor 1	-2.008627
NM_006690,XM_011528500,XM_017027597,XM_017027598	MMP24	matrix metallopeptidase 24	-2.008955
NM_001349413,NM_001349414,NM_001349415,NM_001349416,NM_001349417,NM_001349418,NM_001349419,NM_001349420,NM_001349421,NM_001349422,NM_001349423,NM_001349424,NM_001349425,NM_001349426,NM_001349428,NM_001349429,NM_017644,NR_146169,NR_146170,XM_005247552,XM_017006653,XM_017006655,X	KLHL24	kelch like family member 24	-2.015590

M_017006658,XM_017006661,XM_024453607			
NM_001363598,NM_031412	GABARAPL1	GABA type A receptor associated protein like 1	-2.017838
NR_110997	MIR3936HG	MIR3936 host gene	-2.018128
NM_001205254,NM_001205255,NM_002538,XM_017008913,XM_017008914	OCLN	occludin	-2.022327
NM_005941,XM_024447154	MMP16	matrix metallopeptidase 16	-2.023141
NM_001098272,NM_001324219,NM_001324220,NM_001324222,NM_001324223,NM_001324224,NM_001330663,NM_001364188,NM_002130,XM_011514036,XM_024446038	HMGCS1	3-hydroxy-3-methylglutaryl-CoA synthase 1	-2.023242
NM_005144,NM_018411,XM_005273569,XM_006716367	HR	HR lysine demethylase and nuclear receptor corepressor	-2.024576
NM_002775	HTRA1	HtrA serine peptidase 1	-2.025064
XR_001747518	LOC107984248	uncharacterized LOC107984248	-2.026363
XR_001741421	LOC105379404	uncharacterized LOC105379404	-2.030310
NM_003013	SFRP2	secreted frizzled related protein 2	-2.030420
NM_001039538,NM_001363910,NM_001363911,NM_001363913,NM_001375474,NM_001375493,NM_001375494,NM_001375495,NM_001375496,NM_001375497,NM_001375498,NM_001375499,NM_001375500,NM_001375501,NM_001375502,NM_001375503,NM_001375504,NM_001375505,NM_001375506,NM_001375507,NM_001375508,NM_001375509,NM_001375510,NM_001375526,NM_001375527,NM_001375528,NM_001375529,NM_001375530,NM_001375531,NM_001375532,NM_001375533,NM_001375534,NM_001375535,NM_001375536,NM	MAP2	microtubule associated protein 2	-2.031583

_001375537,NM_001375538,NM_001375539,NM_001375540,NM_001375541,NM_001375542,NM_001375543,NM_001375544,NM_001375545,NM_001375546,NM_001375548,NM_001375551,NM_001375552,NM_001375553,NM_001375554,NM_001375555,NM_001375556,NM_001375557,NM_001375558,NM_001375559,NM_001375583,NM_002374,NM_031845,NM_031847,NR_164694,NR_164695,NR_164696,NR_164697,NR_164698,NR_164699,XM_005246565,XM_005246566,XM_011511195,XM_011511196,XM_011511197,XM_017004112,XM_017004113,XM_017004114,XM_017004116,XM_017004122,XM_017004128,XM_017004129,XM_017004130,XM_017004131,XM_017004138,XM_024452893,XM_024452894,XM_024452895,XM_024452896,XM_024452897,XM_024452899,XM_024452902,XM_024452906,XM_024452907			
NM_020431	TMEM63C	transmembrane protein 63C	-2.032061
NM_019074	DLL4	delta like canonical Notch ligand 4	-2.033686
NM_033199	UCN2	urocortin 2	-2.038341
NM_018398	CACNA2D3	calcium voltage-gated channel auxiliary subunit alpha2delta 3	-2.040024
NM_002148	HOXD10	homeobox D10	-2.040354
NM_001099743,NM_001099744,NM_001099745,NM_001099746,NM_001099747,NM_001099748,NM_001099749,NM_001099750,NM_001099751,NM_001099752,NM_001099753,NM_001099754,NM_001099755,NM_001099756,NM_001330596	SYBU	syntabulin	-2.045594

,NM_001363032,NM_017786,XM_011517154,XM_017013614,XM_017013615			
NM_001367607,NR_160272,XM_011525664,XM_011525665,XM_011525666,XM_024451167,XM_024451168,XR_001753189,XR_001753190,XR_001753191,XR_935057,XR_935058,XR_935059,XR_935060,XR_935061,XR_935062	ANKRD30B	ankyrin repeat domain 30B	-2.045604
NM_015419	MXRA5	matrix remodeling associated 5	-2.046256
NM_001142502,NM_006663,XM_017026177,XM_017026178,XM_017026179	PPP1R13L	protein phosphatase 1 regulatory subunit 13 like	-2.046941
XR_001742939	LOC107986465	uncharacterized LOC107986465	-2.053824
NM_001174060,NM_017784,XM_005264843,XM_005264844,XM_005264845,XM_011533326,XM_017005670	OSBPL10	oxysterol binding protein like 10	-2.055407
NM_002345	LUM	lumican	-2.055410
NM_001322286,NM_001322290,NM_001322291,NM_145756,XM_006722432,XM_017025687,XM_017025688,XM_017025689,XM_024451138	ZNF396	zinc finger protein 396	-2.056831
NR_002924	TBC1D3P1-DHX40P1	TBC1D3P1-DHX40P1 readthrough, transcribed pseudogene	-2.057306
NM_001034173,NR_027752,XM_011537986,XM_011537988,XM_011537989,XM_017018889,XM_017018890	ALDH1L2	aldehyde dehydrogenase 1 family member L2	-2.058476
NM_001025580,NM_001171689,NM_015365	AMMECR1	AMMECR nuclear protein 1	-2.058828
NM_001287437,NM_001287439,NM_001287440,NM_001287441,NM_001287442,NM_001287443,NM_024900,NM_199320,XM_005263232,XM_017008626,XM_017008627,X	JADE1	jade family PHD finger 1	-2.060403

M_024454217,XM_024454218,XM_024454219,XM_024454220,XM_024454221			
gene-RNU6-3P	RNU6-3P	RNA, U6 small nuclear 3, pseudogene	-2.060586
NM_001288748,NM_182904,NR_110031,XR_001747836,XR_001747837,XR_001747838,XR_001747839,XR_001747840	P4HA3	prolyl 4-hydroxylase subunit alpha 3	-2.060774
NM_013281,NM_198391,XM_005260682,XM_011529204,XM_011529205	FLRT3	fibronectin leucine rich transmembrane protein 3	-2.061344
NM_001146254,NM_001146255,NM_001370095,NM_024825,XM_006722902,XM_006722903,XM_011528308,XM_011528309,XM_011528310,XM_011528311,XM_011528313,XM_011528314,XM_011528316,XM_017027313,XM_024451726	PODNL1	podocan like 1	-2.062501
NM_001033553,NM_001033554,NM_001033555,NM_001243438,NM_001243439,NM_152904,XM_005256860,XM_011524075,XM_017025317,XM_017025318,XM_017025319,XM_017025320,XM_017025321,XM_017025322,XM_017025323,XM_017025324,XM_017025325,XR_001752682	SPECC1	sperm antigen with calponin homology and coiled-coil domains 1	-2.069383
NM_001320437,NM_001320441,NM_001350995,NM_001378208,NM_001378209,NM_001378210,NM_152586,NR_135249,NR_135250,NR_146997,NR_146998,NR_165442,NR_165443,XM_005269582,XM_011539368,XM_017015774,XM_017015775,XM_017015777,XM_017015782,XM_017015783,XM_024447832,XM_024447833,XM_024447834,XM_024447835,XM_024447836,XM_024447837,XM_024447838,XM	USP54	ubiquitin specific peptidase 54	-2.073680

_024447839,XM_024447840,XM_024447841,XM_024447842,XM_024447843,XM_024447844,XM_024447845,XM_024447846,XM_024447847,XM_024447848,XM_024447849,XM_024447850,XM_024447851,XM_024447852,XR_001747036,XR_001747037,XR_001747038,XR_001747039,XR_001747041,XR_001747042,XR_001747043,XR_001747044,XR_001747047,XR_001747048,XR_002956959,XR_002956960,XR_002956961,XR_002956962			
NR_138037	HCG20	HLA complex group 20	-2.074976
NM_174911,NR_156466,XM_017013107,XM_017013108	LRATD2	LRAT domain containing 2	-2.076281
NM_001286262,NM_152772,XM_005268767,XM_005268768,XM_011538129,XM_011538130,XM_011538131,XM_011538132,XM_017019127,XM_017019129,XM_017019130,XM_017019131,XM_017019132,XM_017019133	TCP11L2	t-complex 11 like 2	-2.080238
NM_001018053,NM_006212,XM_005273162,XM_024447654,XM_024447655,XM_024447656,XM_024447657	PFKFB2	6-phosphofructo-2-kinase/fructose-2,6-biphosphatase 2	-2.082130
NM_001114309,NM_004433,XM_005244942	ELF3	E74 like ETS transcription factor 3	-2.082778
NM_001142314,NM_001321519,NM_080876,NR_135688	DUSP19	dual specificity phosphatase 19	-2.083806
NM_001004019,NM_001165035,NM_001998,XM_006713026	FBLN2	fibulin 2	-2.085647
NM_001193482,NM_001193483,NM_001193484,NM_001193485,NM_001193488,NM_001371494,NM_001371495,NM_001371496,NM_001371497,NM_001371498,NM_001371499,NM_001371500,NM_004987	LIMS1	LIM zinc finger domain containing 1	-2.086167

XR_929077	LOC105375913	uncharacterized LOC105375913	-2.087410
NM_001142699,NM_001142700,NM_001142702,NM_001206769,NM_001300983,NM_001351274,NM_001351275,NM_001351276,NM_001364,NM_001377966,NM_001377967,NM_001377968,NM_001377970,NM_001377971,NM_001377972,NM_001377973,NM_001377974,NM_001377975,NM_001377976,NM_001377977,NM_001377978,NM_001377979,NM_001377980,NM_001377981,NM_001377982,NM_001377983,NR_165353,XM_005273810,XM_011544778,XM_011544780,XM_011544782,XM_017017254,XM_017017255,XM_017017256,XM_017017257,XM_017017258,XM_017017261,XM_017017262,XM_017017263,XM_017017264,XM_017017265,XM_017017267,XM_017017268,XM_017017269,XM_017017270,XM_017017271,XM_017017273,XM_017017276,XM_017017277,XM_017017279,XM_017017280,XM_017017281,XM_017017284,XM_017017285,XM_017017286,XM_017017287,XM_017017288,XM_017017289,XM_017017290,XM_024448378,XM_024448379	DLG2	discs large MAGUK scaffold protein 2	-2.087764
NM_000393,XM_011510573	COL5A2	collagen type V alpha 2 chain	-2.088943
NR_024569	LOC100130872	uncharacterized LOC100130872	-2.089535
NM_001123066,NM_001123067,NM_001203251,NM_001203252,NM_001377265,NM_001377266,NM_001377267,NM_001377268,NM_005910,NM_016834,NM_016835,NM_016841,NR_165166,XM_005257362,XM_005257365,XM_005257366,	MAPT	microtubule associated protein tau	-2.101268

XM_005257367,XM_005257368,XM_005257369,XM_005257370,XM_005257371			
NM_001008844,NM_001319034,NM_004415	DSP	desmoplakin	-2.106107
NM_001318918,NM_032575	GLIS2	GLIS family zinc finger 2	-2.107611
NM_001201427,NM_015345,XM_006715039,XM_006715040,XM_006715042,XM_006715043,XM_006715045,XM_006715046,XM_017010630	DAAM2	dishevelled associated activator of morphogenesis 2	-2.110467
NM_001884,XM_011543168,XM_017009051,XM_017009052,XM_017009053,XM_017009054	HAPLN1	hyaluronan and proteoglycan link protein 1	-2.111473
NM_000376,NM_001017535,NM_001017536,NM_001364085,NM_001374661,NM_001374662,XM_011538720,XM_024449178	VDR	vitamin D receptor	-2.112666
NM_024080,XM_011511810,XM_017004891,XM_024453132,XM_024453133,XM_024453134	TRPM8	transient receptor potential cation channel subfamily M member 8	-2.115707
NM_001010923,NM_001164685,NM_001164687,NM_001318531,XM_011535814,XM_011535816,XM_017010848,XM_017010849,XM_024446433,XM_024446434	THEMIS	thymocyte selection associated	-2.117576
NR_160425,NR_160426,NR_160427	BMS1P4-AGAP5	BMS1P4-AGAP5 readthough	-2.125681
NM_001256324,NM_001256325,NM_001256326,NM_001256327,NM_001256328,NM_001256329,NM_001256330,NM_001256331,NM_001256332,NM_001256333,NM_001256334,NM_001256359,NM_001256360,NM_001256361,NM_018896,NM_198376,NM_198377,NM_198378,NM_198379,NM_198380,NM_198382,NM_198383,NM_198384,NM_198385,NM_198386,NM_198387,	CACNA1G	calcium voltage-gated channel subunit alpha1 G	-2.128080

NM_198388,NM_198396,NR_046054,NR_046055,NR_046056,NR_046057,NR_046058,XM_006722160,XM_006722161			
NM_001329564,NM_032367,NR_138050	ZBED3	zinc finger BED-type containing 3	-2.130176
NM_001136494,NM_001136495,NM_032800,XM_017002599	C1orf198	chromosome 1 open reading frame 198	-2.134383
NM_001355016,NM_001355017,NM_002609,NR_149150	PDGFRB	platelet derived growth factor receptor beta	-2.136990
NM_001144950,NM_001195267	SSC5D	scavenger receptor cysteine rich family member with 5 domains	-2.137124
NM_014840	NUAK1	NUAK family kinase 1	-2.141097
XM_017023965,XM_017023966	LOC107984138	serine/threonine-protein kinase SMG1-like, transcript variant X1	-2.146690
NM_001042483,NM_012385	NUPR1	nuclear protein 1, transcriptional regulator	-2.149119
NM_001256105,NM_001377271,NM_001377272,NM_003392,XM_011534085,XM_011534086,XM_011534088,XM_011534089,XM_017007127,XM_017007128	WNT5A	Wnt family member 5A	-2.152336
NM_001381939,NM_001381940,NM_001381941,NM_001381942,NM_003247,NR_167744,NR_167745	THBS2	thrombospondin 2	-2.152920
NM_001034954,NM_001034955,NM_001034956,NM_001034957,NM_001290294,NM_001290295,NM_001290296,NM_001290297,NM_001290298,NM_001377197,NM_001377198,NM_001377199,NM_001377200,NM_001377201,NM_001377202,NM_001377203,NM_001377204,NM_001377205,NM_001377206,NM_001377207,NM_001377208,NM_001377209,NM_006434,NM_015385,NM_024991,XM_006717589,XM_006717593,XM_011539140,XM_011539150,XM_011539155,XM_0115	SORBS1	sorbin and SH3 domain containing 1	-2.155289

39167,XM_017015500,XM_017015501,XM_017015502,XM_017015503,XM_017015504,XM_017015505,XM_017015506,XM_017015507,XM_017015508,XM_017015509,XM_017015510,XM_017015511,XM_017015512,XM_017015513,XM_017015514,XM_017015515,XM_017015517,XM_017015523,XM_017015525,XM_017015530,XM_017015532,XM_017015533,XM_017015536,XM_017015537,XM_017015539,XM_017015540,XM_024447769,XM_024447770			
NM_001371116,NM_033393,XM_005263319,XM_011532389	FHDC1	FH2 domain containing 1	-2.156731
XR_002958414	LOC112268244	uncharacterized LOC112268244	-2.158762
NM_001198,NM_182907,XM_006715550,XM_011536062,XM_011536063,XM_011536064,XM_017011187	PRDM1	PR/SET domain 1	-2.159476
NM_001145536	C17orf107	chromosome 17 open reading frame 107	-2.161715
NM_001378183,NM_022068,XM_011525723,XM_011525724,XM_011525725,XM_011525726,XM_017025918,XR_001753259	PIEZO2	piezo type mechanosensitive ion channel component 2	-2.164081
NM_014951,NM_199450,XM_017015937	ZNF365	zinc finger protein 365	-2.165843
NM_173582,XM_011544953	PGM2L1	phosphoglucomutase 2 like 1	-2.169347
NM_001330322,NM_001330323,NM_002146,XM_005257277,XM_006721854,XM_011524708,XM_011524710,XM_011524719,XM_011524720,XM_011524721,XM_011524726,XM_017024560,XM_024450737	HOXB3	homeobox B3	-2.173464
XR_001751773,XR_001751776,XR_001751777,XR_002957755,XR_002957756,XR_002957757,XR_00295	LOC107984805	uncharacterized LOC107984805, transcript variant X3	-2.173941

7758,XR_002957759,XR_002957760,XR_002957761			
NM_181616	KRTAP20-2	keratin associated protein 20-2	-2.178838
NM_002015,XM_011535008,XM_011535010	FOXO1	forkhead box O1	-2.182438
NM_013324,NM_145071,XM_011533329	CISH	cytokine inducible SH2 containing protein	-2.189614
NM_001018100,NM_152451	MYZAP	myocardial zonula adherens protein	-2.190080
NM_001920,NM_133503,NM_133504,NM_133505,NM_133506,NM_133507	DCN	decorin	-2.197143
NM_001406	EFNB3	ephrin B3	-2.198336
NM_001198595,NM_006873	STON1	stonin 1	-2.205280
NM_005559	LAMA1	laminin subunit alpha 1	-2.210911
NM_005266,NM_181703,XM_005272951,XM_017001044	GJA5	gap junction protein alpha 5	-2.216321
NR_037627	LOC100288570	glycosylphosphatidylinositol anchor attachment protein 1 homolog (yeast) pseudogene	-2.217334
NM_007115	TNFAIP6	TNF alpha induced protein 6	-2.218295
XR_001748470,XR_001748471	LOC105369149	uncharacterized LOC105369149, transcript variant X1	-2.219045
NM_001199829,NM_032132,XM_011510054	HORMAD1	HORMA domain containing 1	-2.220132
NM_018557,XM_017004341,XM_017004342,XR_001738778	LRP1B	LDL receptor related protein 1B	-2.227646
NM_032289,XM_011537696,XM_017009976,XM_017009977	PSD2	pleckstrin and Sec7 domain containing 2	-2.230400
NM_019599	TAS2R1	taste 2 receptor member 1	-2.232948
NR_024607	MIR503HG	MIR503 host gene	-2.235301
NM_018899,NM_031883	PCDHAC2	protocadherin alpha subfamily C, 2	-2.236140
NM_001205288,NM_001371340,NR_038099,XM_017003103,XM_017003104,XM_017003105	LIMS4	LIM zinc finger domain containing 4	-2.245679

NM_001012662,NM_001012664,NM_001013251,NM_002394,NR_037193	SLC3A2	solute carrier family 3 member 2	-2.247673
NM_001190709,NM_001854,NM_080629,NM_080630,NR_134980,XM_017000334,XM_017000335,XM_017000336,XM_017000337	COL11A1	collagen type XI alpha 1 chain	-2.250030
NM_001277077,NM_001277078,NM_004272,XM_017010059	HOMER1	homer scaffold protein 1	-2.252413
XR_001753445	LOC107987258	uncharacterized LOC107987258	-2.258488
NM_001278919,NM_001278920,NM_030779,NM_173092,XM_011525308,XM_011525309,XM_011525310,XM_011525311,XM_011525312,XM_011525313,XM_017025175,XM_017025176,XM_017025177,XM_017025178,XM_017025179,XM_017025180,XR_934568	KCNH6	potassium voltage-gated channel subfamily H member 6	-2.259124
NM_001127891,NM_001302508,NM_001302509,NM_001302510,NM_004530	MMP2	matrix metallopeptidase 2	-2.260527
NM_001128933,NM_001128934,NM_001286754,NM_001286755,NM_133477	SYNPO2	synaptopodin 2	-2.260839
XR_001755529,XR_001755530,XR_938223	LOC105377199	uncharacterized LOC105377199, transcript variant X3	-2.277720
NM_001135919,NM_001347960,NM_181785,XM_005266361	SLC46A3	solute carrier family 46 member 3	-2.288436
NM_001285829,NM_001287424,NM_001287435,NM_004364	CEBPA	CCAAT enhancer binding protein alpha	-2.289026
NM_006043,XM_011546001,XM_011546002	HS3ST2	heparan sulfate-glucosamine 3-sulfotransferase 2	-2.292440
NM_033514,NR_027467	LIMS3	LIM zinc finger domain containing 3	-2.292807
NM_172069,XM_017003351,XM_017003352,XM_017003353,XM_024452695,XR_001738620,XR_001738621,XR_001738622,XR_426981,XR_939657	PLEKHH2	pleckstrin homology, MyTH4 and FERM domain containing H2	-2.297711

NM_002193	INHBB	inhibin subunit beta B	-2.299775
NM_005761,NR_037687,XM_006719186,XM_011537730,XM_011537731,XM_017018671,XM_017018672	PLXNC1	plexin C1	-2.305035
NM_022131,XM_017007022	CLSTN2	calsyntenin 2	-2.315063
NM_001378969,NM_001378970,NM_004980,NM_172198,XM_006710629,XM_006710631,XM_006710632,XM_011541425,XM_011541426,XM_011541427,XM_011541428,XM_017001244,XM_017001245	KCND3	potassium voltage-gated channel subfamily D member 3	-2.315785
NM_001309443,NM_001309444,NM_003118	SPARC	secreted protein acidic and cysteine rich	-2.339060
NM_001006932,NM_001318936,NM_001318937,NM_001318938,NM_021135,XM_006715549	RPS6KA2	ribosomal protein S6 kinase A2	-2.342817
NM_004975,XM_006723784,XM_011528799	KCNB1	potassium voltage-gated channel subfamily B member 1	-2.351643
NM_001313726,NM_001313727,NM_031418,XM_011520282,XM_017018118,XM_017018119	ANO3	anoctamin 3	-2.352090
NM_001365079,NM_014631	SH3PXD2A	SH3 and PX domains 2A	-2.357590
NR_036485	SBF2-AS1	SBF2 antisense RNA 1	-2.378054
NM_001114396,NM_001351060,NM_001351062,NM_001351063,NM_002262,NM_007334,NR_147038,NR_147039,NR_147040,XM_006719067,XM_011520650,XM_011520651,XM_017019285,XM_017019286,XM_017019287,XM_017019288,XM_017019289,XM_024448974,XR_001748696,XR_001748697	KLRD1	killer cell lectin like receptor D1	-2.385892
NM_001324112,NM_004820,XM_017014002	CYP7B1	cytochrome P450 family 7 subfamily B member 1	-2.388021
NM_003469	SCG2	secretogranin II	-2.391282

NM_001278613,NM_001278615,NM_002587,NM_032420,XM_005268452,XM_005268454,XM_005268455,XM_017009517,XM_017009518	PCDH1	protocadherin 1	-2.393431
NR_033937	LOC100132077	uncharacterized LOC100132077	-2.393498
NM_002114,XM_011514546,XM_011514547,XM_011514548,XM_011514549,XM_011514550,XM_011514551,XM_011514552,XM_011514553,XM_011514555,XM_017010800,XM_017010801,XM_017010802,XM_017010803,XM_017010804,XR_001743372	HIVEP1	HIVEP zinc finger 1	-2.400736
NR_125967,NR_125968	LINC01719	long intergenic non-protein coding RNA 1719	-2.401426
NM_001346590,NM_001346591,NM_001346592,NM_001346593,NM_001346594,NM_005542,NM_198336,NM_198337	INSIG1	insulin induced gene 1	-2.402348
NM_000090	COL3A1	collagen type III alpha 1 chain	-2.411601
NM_001142776,NM_024111,XM_024450045,XM_024450046,XM_024450047	CHAC1	ChaC glutathione specific gamma-glutamylcyclotransferase 1	-2.412216
NM_025130,XM_011540195,XR_001747209	HKDC1	hexokinase domain containing 1	-2.422935
NM_000093,NM_001278074,XM_017014266,XR_001746183	COL5A1	collagen type V alpha 1 chain	-2.429304
XR_938114	LOC105372976	uncharacterized LOC105372976	-2.440156
NM_020690	ANKHD1-EIF4EBP3	ANKHD1-EIF4EBP3 readthrough	-2.461436
NM_001007237,NM_001542,XM_005270794,XM_006710593,XM_011541315,XM_011541316	IGSF3	immunoglobulin superfamily member 3	-2.468742
NM_001134,NM_001354717	AFP	alpha fetoprotein	-2.476212
NM_020697	KCNS2	potassium voltage-gated channel modifier subfamily S member 2	-2.477556
NM_181726,XM_017008176	ANKRD37	ankyrin repeat domain 37	-2.478966

XR_001743969	LOC107986566	uncharacterized LOC107986566	-2.484104
NR_102710,NR_102711	XXYLT1-AS2	XXYLT1 antisense RNA 2	-2.507462
NM_001382000,NM_014695,NR_130142,NR_167766,XM_017025429,XM_017025430,XM_024451045,XM_024451046,XR_001752702,XR_001752703,XR_001752704	CCDC144A	coiled-coil domain containing 144A	-2.511413
NM_001305581,NM_032024,NR_131178	LRMDA	leucine rich melanocyte differentiation associated	-2.515547
NR_130726	LINC00243	long intergenic non-protein coding RNA 243	-2.522852
XR_001739239,XR_001739240	LOC102723825	uncharacterized LOC102723825, transcript variant X1	-2.523694
NM_005257	GATA6	GATA binding protein 6	-2.528968
NM_000827,NM_001114183,NM_001258019,NM_001258020,NM_001258021,NM_001258022,NM_001258023,NM_001364165,NM_001364166,NM_001364167,NR_047578,NR_157093,XM_017009392	GRIA1	glutamate ionotropic receptor AMPA type subunit 1	-2.542307
NR_147989	CZ1P-ASNS	CZ1P-ASNS readthrough	-2.544522
NM_001135599,NM_003238,NR_138148,NR_138149	TGFB2	transforming growth factor beta 2	-2.544980
NM_002240	KCNJ6	potassium inwardly rectifying channel subfamily J member 6	-2.575871
NR_002594	SLC7A5P2	solute carrier family 7 member 5 pseudogene 2	-2.577309
NM_001164104,NM_001164105,NM_001164106,NM_018995,XM_005261923,XM_011530696,XM_011530697,XM_011530698,XM_011530699,XM_011530700,XM_011530701,XM_011530702,XM_011530703,XM_011530704,XM_017028833,XM_017028834,XM_017028835,XM_017028836,XM_017028837,XR_002958704	MOV10L1	Mov10 like RISC complex RNA helicase 1	-2.580851

NM_198182,XM_005246159,XM_006711882,XM_006711884,XM_011510343,XM_017003900,XM_017003901,XR_001738726,XR_001738727,XR_001738728	GRHL1	grainyhead like transcription factor 1	-2.583527
NM_015719,XM_011528042,XM_017026849	COL5A3	collagen type V alpha 3 chain	-2.595586
NR_040058	RAD51-AS1	RAD51 antisense RNA 1	-2.598920
NM_014737,NM_170774,XM_005260895,XM_011529410,XM_011529411,XM_011529412,XM_017028149,XM_017028150,XM_017028151,XM_017028152,XM_017028153	RASSF2	Ras association domain family member 2	-2.607455
XM_011513341	LOC105374013	endogenous retrovirus group K member 5 Gag polyprotein	-2.623188
NM_001112800,NM_001112801,NM_001112802,NM_001252624,NM_001351483,NM_001351484,NM_001351485,NM_001351486,NM_001351487,NM_001351488,NM_001351489,NM_001351490,NM_001351491,NM_001351492,NM_001351493,NM_001351494,NM_001372263,NM_021097,XM_006712082,XM_006712083,XM_006712084,XM_006712085,XM_011533050,XM_011533054,XM_011533055,XM_011533056,XM_011533058,XM_017004745,XM_017004746,XM_017004749,XM_017004750,XM_017004751,XM_017004752,XM_017004753,XM_017004755,XM_017004764	SLC8A1	solute carrier family 8 member A1	-2.626330
NM_001350120,NM_001350121,NM_006379	SEMA3C	semaphorin 3C	-2.631132
NR_024008	WWC2-AS2	WWC2 antisense RNA 2	-2.631324
NM_001202439,XM_011520072,XM_011520073,XM_011520074,XM_011520075	NCR3LG1	natural killer cell cytotoxicity receptor 3 ligand 1	-2.653727

NR_003051	RMRP	RNA component of mitochondrial RNA processing endoribonuclease	-2.655337
XR_243478,XR_429779,XR_933760,XR_933761	LOC101928230	uncharacterized LOC101928230, transcript variant X4	-2.669775
NM_001135934,NM_001135935,NM_001135936,NM_001286665,NM_001286666,NM_001286667,NM_001330517,NM_006475,XM_005266232,XM_017020355,XM_017020356	POSTN	periostin	-2.702380
XR_001753371,XR_935292,XR_935293	LOC105372026	uncharacterized LOC105372026, transcript variant X2	-2.716361
NR_110681	LOC101927604	uncharacterized LOC101927604	-2.722208
NM_001142569,NM_001367289,NM_001367290,NM_018265,XM_011509754,XM_011509755,XM_011509756	INAVA	innate immunity activator	-2.726625
NM_000253,NM_001300785	MTTP	microsomal triglyceride transfer protein	-2.737760
NM_001287491,NM_001366022,XM_011532682,XM_011532683,XM_011532684,XM_011532685,XM_011532686,XM_011532687,XM_011532688,XM_011532690,XM_017003566,XM_024452745,XM_024452746,XM_024452747	TET3	tet methylcytosine dioxygenase 3	-2.751050
NR_105059,NR_105060	CARMN	cardiac mesoderm enhancer-associated non-coding RNA	-2.755294
gene-ANKRD30BP1	ANKRD30BP1	ankyrin repeat domain 30B pseudogene 1	-2.765422
XR_001753953,XR_001753954	LOC107985305	uncharacterized LOC107985305, transcript variant X1	-2.776124
XR_001754477	LOC107985414	uncharacterized LOC107985414	-2.780701
gene-RPL7AP16	RPL7AP16	ribosomal protein L7a pseudogene 16	-2.787704
NM_152754,XM_011515960,XM_011515961,XM_017011873	SEMA3D	semaphorin 3D	-2.796352
XR_001739683	LOC107985942	uncharacterized LOC107985942	-2.838826

NM_001303110,NM_001845,XM_011521048	COL4A1	collagen type IV alpha 1 chain	-2.844856
NR_135738	GTF2IP23	general transcription factor IIi pseudogene 23	-2.883453
NR_132969,NR_132970,NR_132971	LOC145694	uncharacterized LOC145694	-2.900540
NR_164111	LMO7DN	LMO7 downstream neighbor	-2.909886
NM_001317184,NM_001317185,NM_001317186,NM_004360	CDH1	cadherin 1	-2.911172
NM_001029851,NM_001029852,NM_001029853,NM_001029854,NM_001349748,NM_001349749,NM_001349750,NM_001349751,NM_001349752,NM_001349753,NM_001376062,NM_001376063,NM_001376064,NM_001376065,NM_001376066,NM_001376067,NM_001376068,NM_001376069,NM_001376070,NM_001376071,NM_001376072,NM_001376073,NM_001376074,NM_001376075,NM_003719,XM_006714726,XM_011543699,XM_011543700,XM_011543704	PDE8B	phosphodiesterase 8B	-2.944585
NR_002836	PGM5P2	phosphoglucomutase 5 pseudogene 2	-3.037253
gene-XPOTP1	XPOTP1	exportin for tRNA pseudogene 1	-3.107160
NM_001371457,NM_002410,XM_005263669,XM_005263670,XM_006712534,XM_011511199,XM_011511200,XM_011511201,XM_011511202,XM_011511204,XM_017004147,XM_017004148,XM_017004149	MGAT5	alpha-1,6-mannosylglycoprotein 6-beta-N-acetylglucosaminyltransferase	-3.107495
NM_001017961,NM_001320302,NR_135199,NR_163271,XM_017000417	FAM78B	family with sequence similarity 78 member B	-3.122001
NM_001172651,NM_003451	ZNF177	zinc finger protein 177	-3.177059
NR_027142,NR_027143	LOC440895	two pore channel 3 pseudogene	-3.235603

NR_003334	SNORD116-20	small nucleolar RNA, C/D box 116-20	-3.318427
NM_018903,NM_031864	PCDHA12	protocadherin alpha 12	-3.325564
NR_103839	FRY-AS1	FRY antisense RNA 1	-3.480826
NM_001300828,NM_019035,XM_006714239,XM_017008311	PCDH18	protocadherin 18	-3.481006
NM_001347887,NM_001347888,NM_022140,NR_144931,XM_011543531,XM_011543532,XM_011543533,XM_017009689,XR_001742173	EPB41L4A	erythrocyte membrane protein band 4.1 like 4A	-3.491466
NR_135198	LOC729652	uncharacterized LOC729652	-3.625887
NM_001316676,NM_001316677,NM_001323354,NM_001323355,NM_001323356,NM_001323357,NM_006504,NM_130435,XM_005252691,XM_011539994,XM_011539995,XM_011539996,XM_011539998,XM_017016467,XM_017016468,XM_017016469,XM_024448092,XM_024448093,XR_002956995,XR_002956996	PTPRE	protein tyrosine phosphatase receptor type E	-3.636282
NM_005242,XM_017009223	F2RL1	F2R like trypsin receptor 1	-3.647768
XR_001753444	LOC107985147	uncharacterized LOC107985147	-3.780282
gene-LOC389473	LOC389473	chromosome 5 open reading frame 13 pseudogene	-3.850993
NM_001304392,NM_002996	CX3CL1	C-X3-C motif chemokine ligand 1	-3.985463
NM_002281	KRT81	keratin 81	-7.882391
